# A co-culture genome-wide RNAi screen with mammary epithelial cells reveals transmembrane signals required for growth and differentiation

**DOI:** 10.1186/s13058-014-0510-y

**Published:** 2015-01-09

**Authors:** Angela Burleigh, Steven McKinney, Jazmine Brimhall, Damian Yap, Peter Eirew, Steven Poon, Viola Ng, Adrian Wan, Leah Prentice, Lois Annab, J Carl Barrett, Carlos Caldas, Connie Eaves, Samuel Aparicio

**Affiliations:** 1 Department of Pathology and Laboratory Medicine, University of British Columbia, and BC Cancer Agency, 675 West 10th Avenue, Vancouver, BC V5Z 1L3 Canada; 2 Centre for Translational and Applied Genomics, BC Cancer Agency, 600 West 10th Avenue, Vancouver, BC V5Z 4E6 Canada; 3 Chromatin and Gene Expression Section, Research Triangle Park, Durham, NC 27709 USA; 4 Laboratory of Molecular Carcinogenesis, National Institute of Environmental Health Sciences, National Institutes of Health, Research Triangle Park, Durham, NC 27709 USA; 5 Cancer Research UK Cambridge Research Institute and Department of Oncology, University of Cambridge, Li Ka Shin Centre, Cambridge, CB2 0RE UK; 6 Terry Fox Laboratory, BC Cancer Agency, Vancouver, BC V5Z 1L3 Canada

## Abstract

**Introduction:**

The extracellular signals regulating mammary epithelial cell growth are of relevance to understanding the pathophysiology of mammary epithelia, yet they remain poorly characterized. In this study, we applied an unbiased approach to understanding the functional role of signalling molecules in several models of normal physiological growth and translated these results to the biological understanding of breast cancer subtypes.

**Methods:**

We developed and utilized a cytogenetically normal clonal line of *hTERT* immortalized human mammary epithelial cells in a fibroblast-enhanced co-culture assay to conduct a genome-wide small interfering RNA (siRNA) screen for evaluation of the functional effect of silencing each gene. Our selected endpoint was inhibition of growth. In rigorous postscreen validation processes, including quantitative RT-PCR, to ensure on-target silencing, deconvolution of pooled siRNAs and independent confirmation of effects with lentiviral short-hairpin RNA constructs, we identified a subset of genes required for mammary epithelial cell growth. Using three-dimensional Matrigel growth and differentiation assays and primary human mammary epithelial cell colony assays, we confirmed that these growth effects were not limited to the 184-*hTERT* cell line. We utilized the METABRIC dataset of 1,998 breast cancer patients to evaluate both the differential expression of these genes across breast cancer subtypes and their prognostic significance.

**Results:**

We identified 47 genes that are critically important for fibroblast-enhanced mammary epithelial cell growth. This group was enriched for several axonal guidance molecules and G protein–coupled receptors, as well as for the endothelin receptor *PROCR*. The majority of genes (43 of 47) identified in two dimensions were also required for three-dimensional growth, with *HSD17B2*, *SNN* and *PROCR* showing greater than tenfold reductions in acinar formation. Several genes, including *PROCR* and the neuronal pathfinding molecules *EFNA4* and *NTN1*, were also required for proper differentiation and polarization in three-dimensional cultures. The 47 genes identified showed a significant nonrandom enrichment for differential expression among 10 molecular subtypes of breast cancer sampled from 1,998 patients. *CD79A*, *SERPINH1*, *KCNJ5* and *TMEM14C* exhibited breast cancer subtype–independent overall survival differences.

**Conclusion:**

Diverse transmembrane signals are required for mammary epithelial cell growth in two-dimensional and three-dimensional conditions. Strikingly, we define novel roles for axonal pathfinding receptors and ligands and the endothelin receptor in both growth and differentiation.

**Electronic supplementary material:**

The online version of this article (doi:10.1186/s13058-014-0510-y) contains supplementary material, which is available to authorized users.

## Introduction

The identification of distinct cell types that appear to be hierarchically organized in the mammary epithelial glands of healthy women is now well established [1]. This hierarchy is defined largely by two prospectively separable subsets of cells that generate colonies containing only one or both lineages (myoepithelial and/or luminal) of cells that make up the bulk of the normal mammary gland structure. The bipotent, clonogenic, progenitor-enriched basal cell fraction also contains putative human mammary stem cells identified in xenotransplantation assays [2,3]. The ability of human mammary cells to be propagated both *in vitro* and *in vivo* at limited densities is known to be markedly enhanced by the presence of fibroblast ‘feeders’ [2,4,5]. These and many other studies have shown that fibroblast interactions are important to the growth of mammary epithelial cells [6-12]. However, a comprehensive characterization of the mechanisms by which fibroblasts regulate the growth and functional organization of normal mammary epithelial cells has been lacking.

Genome-wide RNA interference (RNAi, small interfering RNA (siRNA)) screens offer an attractive strategy by which to investigate such questions. They have previously been used with success to identify mediators of Ras oncogene-induced senescence, suppressors of p16 gene expression, genes that regulate cell migration and cell survival genes in mammary cells [13-16]. This type of investigation is nevertheless dependent on a source of cells that can be obtained in large numbers and readily transfected. Because primary normal mammary epithelial cells, even those derived from human mammoplasties, do not satisfy either of these requirements, we sought an alternative in a clonal diploid isolate of *hTERT*-immortalized cells [17] that we found remains dependent on fibroblast stimulation for its rapid growth when cultured at low density. By combining automated imaging with siRNA screening of these cells, we identified 43 signal-transducing receptors and secreted factors with functionally validated roles in mediating the *in vitro* growth of primary normal human mammary epithelial cells.

## Methods

### Cell lines

Passage 6 184-*hTERT* polyclonal infection pool mammary epithelial cells (obtained from [18]) were contributed to the study by CB and LA. As described previously [18], these pools were generated from anonymised primary mammary epithelial sample 184 (see [18]) and not subject to specific institutional review board approval. We generated the monoclonal cell lines (184-*hTERT*-L9 or 184-*hTERT*-E11) and used the 184-*hTERT*-L9 cell line to generate subsequent polyclonal cell lines (stably infected with lentiviruses or NucLight Red (Essen BioScience, Ann Arbor, MI, USA), for example). The experiments were conducted under University of British Columbia Research Ethics Board protocols H06-0289, H06-0210 and B13-0126.

### Cell culture

Passage 6 184-*hTERT* cells [18] were cloned in 96-well plates and subcultured in serum-free mammary epithelial cell basal media (MEBM; Lonza, Walkersville, MD, USA) supplemented with the mammary epithelial cell growth media in the SingleQuots kit (Lonza), 5 μg/ml transferrin (Sigma-Aldrich, St Louis, MO, USA) and 10^−5^ M isoproterenol (Sigma-Aldrich), referred to as *mammary epithelial cell growth medium* (MEGM).

### Immunofluorescence

Multicolour fluorescence *in situ* hybridization (FISH) was performed as previously described [19]. Immunofluorescence cell staining in three-dimensional Matrigel cultures was performed as previously described [20] with primary antibodies to GM130 (BD Biosciences, San Jose, CA, USA), CD49f and MUC1 (STEMCELL Technologies, Vancouver, BC, Canada), as well as Alexa Fluor 680–conjugated secondary antibodies (Invitrogen, Carlsbad, CA, USA). Cells were counterstained with Oregon Green 488 or Alexa Fluor 546 phalloidin (Invitrogen) and DRAQ5 nuclear staining prior to imaging on a confocal laser scanning microscope (Nikon Instruments, Melville, NY, USA). For calcein acetoxymethyl ester (calcein AM) and ethidium homodimer 1, 21-day Matrigel cultures were stained unfixed for 20 minutes and counterstained with Hoeschst 33342 (Invitrogen). Immunofluorescence staining of cells in three-dimensional Matrigel cultures cultured for 3 weeks was performed with primary antibodies to E-cadherin (E-cad; Calbiochem, San Diego, CA, USA), GM130 (BD Biosciences), CD49f (STEMCELL Technologies) and Alexa Fluor 680–conjugated secondary antibodies (Invitrogen) and imaged on a Nikon confocal laser scanning microscope. Colonies were counted at five discrete, randomly chosen positions per well using a Nikon confocal laser scanning microscope. Only discrete, well-separated structures were counted. In the cases where two colonies touched or merged, both colonies were ignored for counting purposes. For caspase 3 staining, three-dimensional Matrigel cultures were formalin-fixed and paraffin-embedded, and sections were stained with caspase 3 antibody (Cell Signaling Technology, Danvers, MA, USA). To quantify the proportion of structures with wild-type epithelial organization and polarization, RNAi-treated samples were scored and compared to wild-type localization (see Additional file 1: Figure S7 and Additional file 2: Figure S8A) for examples of each category. For CD49f, the presence of a single basement membrane type of immunoreactive structure was considered the wild type. For GM130 immunoreactivity, wild-type polarization was deemed to be signal-localized between the edge of the colony and an unstained lumen. Lack of polarization would be reflected in the indistinguishable staining patterns between the outer and inner cell layers.

### Genome-wide siRNA screen protocol

Black-walled clear-bottom 96-well plates (Greiner Bio-One, Monroe, NC, USA) were seeded with 3,000 freshly irradiated NIH 3T3 cells per well in Dulbecco’s modified Eagle’s medium with 5% foetal bovine serum. After 24 hours, the medium was aspirated at a low flow rate with a multichannel vacuum aspirator and replaced with 550 184-*hTERT*-L9 cells per well in MEGM without bovine pituitary extract (BPE) added. After an additional 24 hours, Lipofectamine 2000 reagent–siRNA complexes were generated.

siRNAs were purchased from the siGENOME library (Dharmacon, Lafayette, CO, USA) as deconvolved sets of four individual siRNAs and resuspended at 10 μM in 1× siRNA buffer (Dharmacon) as described elsewhere [21]. Lipofectamine 2000 transfection reagent (Invitrogen) was diluted in MEBM and incubated for 5 minutes prior to mixture with an equal volume of prediluted siRNA in MEBM. Complexes were allowed to form for 20 minutes before they were added directly to the cells at a final concentration of 30 nM siRNA and 0.3 μl of transfection reagent per well. The control wells were static in position and were composed of Lipofectamine alone, siControl-3 and a siRNA pool targeting PLK1. The entire 96-well plates of these controls were staggered throughout the duration of this screen to allow for statistical correction of plate positional effects. After an additional 4 days of growth, cells were fixed with 4% paraformaldehyde and stained with 1 μg/ml 4′,6-diamidino-2-phenylindole (DAPI) prior to being imaged on an IN Cell Analyzer (GE Healthcare Bio-Sciences, Pittsburgh, PA, USA) using the 10× lens objective (numerical aperture, 0.45) with charge-coupled device pixel binning. Twenty-one fields per well were collected using HQ360/40 excitation and HQ535/50 emission filters with a multi-bandpass dichroic filter (Q505lp; Chroma Technology, Bellows Falls, VT, USA). For each image field, a single focal plane was captured using a hardware (laser/photodetector) autofocusing algorithm, which estimated the surface area on which the cells were lying. An image segmentation and postprocessing software workflow was developed using CellProfiler [21]. Cell counts per well produced in the Cell Profiler image analysis were further processed using quantile normalization to correct for data distributional differences induced by factors such as time-dependent stain degradation and other plate-handling artefacts. Quantile-normalized data were then analysed using statistical linear mixed effects regression models to compare cell counts under the siRNA condition with counts under the control condition, adjusted for technical artefacts of well position and plate effects, thereby yielding plate-normalized growth effect estimates for each siRNA. Well position effects were assessed by screening additional plates containing the same control condition in all wells. Plate effects were assessed by using multiple plates for each condition, thereby allowing for adjustment of plate-to-plate variability in the statistical model. The model-estimated cell count under the siRNA condition was then divided by the model-estimated cell count under the control condition to produce an overall measure of relative effect. Measured effects were ranked from smallest to largest, and the latter (those reducing cell growth by 75% or more) were selected for further study. Enrichment network analysis was performed as previously described [22] using the Reactome Functional Interactome plugin in Cytoscape v2.8.1 [23,24].

### Lentiviral transduction procedure

184-*hTERT*-L9 cells were transduced at an estimated multiplicity of infection (MOI) of 5:1 with 8 μg/ml polybrene. After 18 hours at 37°C, cells were washed, and, after 24 hours in MEGM, they were selected with 2 μg/ml puromycin in MEGM (replaced every 24 to 48 hours). Dissociated primary human mammary epithelial cells were infected with an estimated MOI of 10. Transduction was conducted in suspension with 8 μg/ml polybrene at a density of 5 × 10^5^ cells in 100 μl of serum-free 7 medium (DMEM/F12 (STEMCell Technologies) supplemented with 5% FBS, 2 mM glutamine (Gibco), 0.1% w/v BSA, 10 ng/ml EGF (Sigma), 10 ng/ml cholera toxin (Sigma), 1 μg/ml insulin (Sigma), 0.5 μg/ml hydrocortisone (Sigma)) with 50 μg/ml GA-1000 (Lonza). After 18 hours at 37°C, cells were washed and counted and equal numbers plated into two-dimensional colony-forming cell assays.

### Primary mammary tissue

Discarded tissue was collected from premenopausal women (ages 19 to 40 years) who provided informed consent as approved by the University of British Columbia Research Ethics Board, as previously described [2]. Suspensions selectively enriched in bipotent progenitor cells were obtained by fluorescence-activated cell sorting (FACS) of cells positively costained with an allophycocyanin-conjugated rat antibody to human CD49f (eBioscience, San Diego, CA, USA) and a phycoerythrin-conjugated mouse antibody to human epithelial cell adhesion molecule (EpCAM; eBioscience) [2]. Hematopoietic stem cells and endothelial cells were eliminated using antibodies to human CD45 and human CD31 (eBioscience), respectively.

### Proliferation measurement by 5-ethynyl-2′-deoxyuridine incorporation

To determine the proliferation rate, 184-*hTERT*-L9 cells stably infected with NucLight Red were seeded into wells of 24-well plates (16,750 cells per well). Twenty-four hours after plating, the cells were transfected with siRNAs (30 nM concentration) and cultured in the IncuCyte ZOOM incubator (Essen BioScience). Twenty hours after transfection, the siRNA-containing medium was removed and replaced with standard 184-*hTERT* culture medium. Sixty-eight hours after transfection, 5-ethynyl-2′-deoxyuridine (EdU) was added to each well to a final concentration 40 μM. After 1 hour, wells were washed with phosphate-buffered saline, then the cells were harvested with a trypsin/ethylenediaminetetraacetic acid mixture. To ensure sufficient cell numbers for FACS, 1 × 10^5^ nonfluorescent 184-*hTERT* cells were added to the cells harvested from each well. EdU incorporation was then detected by staining with the Click-iT EdU Alexa Fluor 488 Flow Cytometry Assay Kit (Life Technologies, Grand Island, NY, USA) according to the manufacturer’s instructions, followed by FACS acquisition on a BD FACSAria III cell sorter (BD Biosciences). DAPI was used to detect DNA content and to gate out cell fragments. The percentage of test cells that incorporated EdU was determined by gating on the NucLight Red–positive cell population and applying an EdU-positive gate set with reference to negative control (carrier) cells processed with the Click-iT EdU assay but not previously exposed to an EdU pulse.

### Determination of proliferation and apoptosis by live cell imaging

Where appropriate, 184-*hTERT*-L9 cells were stably infected with NucLight Red, which marks the nuclei red to distinguish them from unlabelled irradiated feeders used the in co-culture experiments. To control for feeder effects, we conducted the experiment using both feeder-free and feeder-containing conditions, with BPE medium supplement (see details in methods) used for the nonfeeder condition. For the feeder-present condition, 3,000 irradiated feeders per well were plated in 96-well plates 24 hours prior to plating 550 184-*hTERT*s stably expressing NucLight Red. Transfection of 30 nM siRNA (complexing as described in methods) was performed 24 hours later, and the complexes were washed out after 24 hours, with the addition of CellPlayer Caspase-3/7 reagent (Essen BioScience), which labels apoptotic cells green, detecting caspases 3 and 7. The plate was then imaged using an IncuCyte ZOOM live cell microscope (Essen BioScience), and images were taken every 4 hours for an additional 84 hours. The data were analysed as follows. Triplicate red cell objects (representing *hTERT* with NucLight Red) and green objects (apoptotic cells with activated caspase 3, as the percentage of red objects) were counted (counts per square millimetre) using the GraphPad Prism statistical software suite (GraphPad Software, La Jolla, CA, USA), and the respective areas under the curve (AUCs) for serial measurements were calculated. Where comparisons were made, the AUC values were subjected to a one-way analysis of variance test to compare the mean of each siRNA to the control condition, in which only Lipofectamine 2000 transfection reagent was used to determine significance.

### Gene association analyses

Expression and patient outcome data for 1,996 breast cancer patients were obtained from the Molecular Taxonomy of Breast Cancer International Consortium (METABRIC) study (European Genotype-phenome Archive study accession number: EGAS00000000083) [25]. Relative hazard estimates for cases with high versus low gene expression were obtained using a Cox proportional hazards model with the binarized expression variable and stratified by integrative cluster (IntClust) breast cancer subtype groups to mitigate for the nonproportional hazards exhibited between various IntClust groups. Expression variables were binarized at the 15%, 25%, 50% and 75% quantiles, and Akaike information criteria (AIC) were calculated using a Cox model containing the binarized expression variable. The binarization cut point was chosen as the quantile yielding the minimum AIC value. In the case that the minimum and maximum AIC values differed by less than 3.0, the median expression level was used as the cut point to ensure adequate case counts within the breast cancer subtype groups. To identify potential instances of interaction between expression level and breast cancer subtype (high versus low expression showing improved survival in one subgroup and poorer survival in another subgroup), a Cox model with binarized expression, IntClust subgroups and their interaction terms was fitted and compared to a Cox model containing IntClust subgroups only, yielding an omnibus test of survival difference due to the biomarker. P-values across all 46 fitted models were adjusted for multiple comparisons using the method of Benjamini and Hochberg [26], and significant findings after adjustment for multiple comparisons were identified. To guard against the issue of changing hazards over time and nonproportional hazards between IntClust subgroups, each binarized biomarker was tested using the G-rho rank test procedure stratified by IntClust subgroups and setting ρ = 1 to place heavier weight on earlier time point observations [27]. P-values from all 46 G-rho tests were adjusted for multiple comparisons, and genes with low false discovery rates were identified.

For analysis of transcripts in flow-sorted mammary epithelial cell lineages, we made use of the NIH and Canadian Roadmap Epigenomics mammary epithelial cell RNA-seq libraries. The consortium data generation protocols and data can be accessed online [28,29]. The flow sorting of mammary epithelial subsets was performed using CD10, MUC1 and CD73 as described previously [30].

### Expression data

Expression data processing and normalization are described in the supplementary materials of the METABRIC study [25]. Expression data were additionally centred and scaled within the study data centre as follows:

$$ {r}_{ijk}=\frac{e_{ijk}- me{d}_{ik}}{ig{s}_{jk}} $$

where *e*_*ijk*_ is the normalized expression value for subject *i*, gene target *j*, in data centre *k*; *med*_*ik*_ is the median expression value for gene target *j* in data centre *k*; and *igs*_*jk*_ is the interquartile spread for gene target *j* in data centre *k*. (Interquartile spread is the difference between the 75th-percentile value and the 25th-percentile value, a robust measure of standard deviation.) Heat maps of this robustly centred and scaled data were generated with complete linkage clustering of gene targets indicated on the vertical axis. To yield comparable colour schemes across all genes in the heat map, values less than −3.0 were set to −3.0 and values greater than 3.0 were set to 3.0. Expression value differences among breast cancer subtype groups (IntClust and PAM50) were assessed using the Kruskal-Wallis test. Beanplots [31] of expression values within breast cancer subtype groups for each of the 47 final identified gene targets were ordered in Kruskal-Wallis test *P*-value order.

## Results

### Isolation and characterization of a cytogenetically normal clone of 184-*hTERT* cells

**Figure 1:**
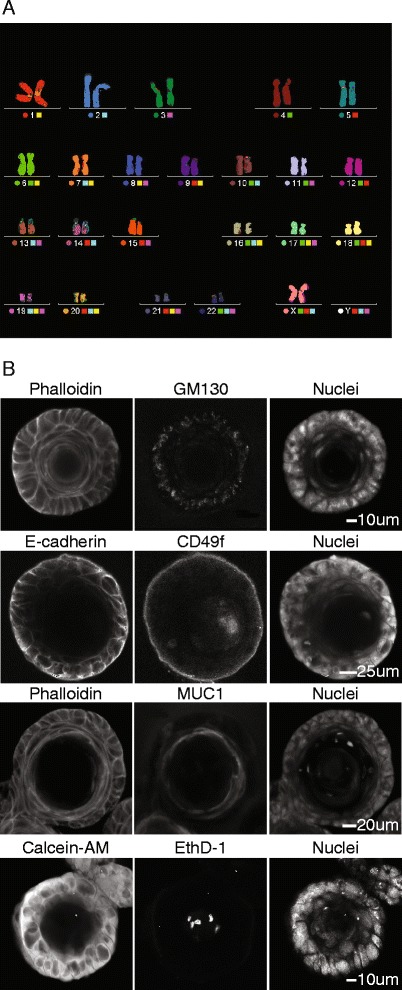
**184-** ***hTERT*** **-L9 cells have a normal karyotype and form acini in three dimensions. (A)** 184-*hTERT*-L9 cells were arrested in metaphase using colcemid prior to multicolour fluorescence *in situ* hybridization analysis. Metaphase spreads had a normal diploid chromosome complement devoid of numerical or structural changes (*n* = 7). **(B)** Cells cultured in three-dimensional Matrigel were fixed after 21 days and probed with antibodies targeting GM130 (apical polarity), CD49f (basal polarity) and MUC1 (luminal marker). Either Oregon Green–labelled phalloidin or an antibody targeting E-cadherin was used as a counterstain, with DRAQ5 nuclear stain. For viability assessment, unfixed structures were incubated for 20 minutes with calcein AM, ethidium homodimer 1 (EthD-1) and Hoechst 33452 immediately prior to being imaged on a Nikon confocal laser scanning microscope.

We sought a cell line model of primary human mammary epithelial cells that would display fibroblast-enhanced growth at low cell density and would also be readily amenable to high-throughput, genome-wide genetic manipulation. Preliminary data indicated that this was a retained feature of 184-*hTERT* cells. However, those widely available are karyotypically abnormal and may be chromosomally unstable [32]. Therefore, we obtained an early passage of the original 184 primary cell pool transduced with *hTERT* [17]. After cloning approximately 80 independent lines, we isolated a clone (184-*hTERT*-L9) in which all cells showed a stable normal (46, XX) karyotype, a copy number diploid genome (Figure 1A and Additional file 3: Figure S1D) and a basal phenotype (K5+, E-cad+, MUC1− and oestrogen receptor α–negative (ERα−)) (Additional file 3: Figures S1B and S1C) with expression of epidermal growth factor receptor and E-cad (Additional file 3: Figure S1B), but not of keratin 18, human epidermal growth factor receptor 2 (HER2) or ERα (Additional file 3: Figures S1B and S1C). 184-*hTERT*-L9 cells also express wild-type p53 (Additional file 3: Figure S1B), but they show silenced p16 expression (Additional file 3: Figure S1C) and have a single integration site for *hTERT* (Additional file 3: Figure S1A).

**Figure 2:**
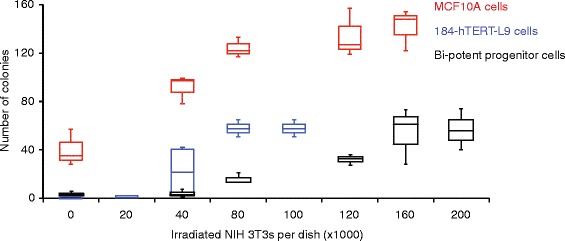
**Fibroblasts influence mammary epithelial cell growth.** Colony-forming assays were performed with an increasing density of irradiated NIH 3T3 cells plated alongside a set density of prospectively fractionated human bipotent mammary progenitor cells (black; *n* = 6 patient samples), 184-*hTERT*-L9 cells (blue; *n* = 4) and MCF10A cells (red; n = 4). The whisker ends on the box plots denote the lowest and highest data within 1.5× the interquartile range of the lower and upper quartiles, respectively.

To quantify fibroblast-dependent growth [33-35], we compared 184-*hTERT*-L9 cells with primary basal progenitor cell-enriched fractions (CD49f^high^EpCam^−^) sorted from dissociated reduction mammoplasty tissue. When plated in a colony-forming cell assay in co-culture with an increasing density of irradiated fibroblasts, a dose-dependent growth response was observed (Figure 2) for both 184-*hTERT*-L9 and primary mammary epithelial cells at low plating densities. This behaviour is distinct from many immortalized mammary epithelial cells lines, including MCF10A (Figure 2), in which colony formation at low epithelial cell density occurs with high efficiency independent of a fibroblast feeder layer (growth at 0 fibroblasts).

We next compared the three-dimensional growth characteristics of 184-*hTERT*-L9 with flow-sorted primary mammary epithelial cells and with MCF10A cells [36]. When placed into three-dimensional Matrigel culture, 184-*hTERT*-L9 cells formed spherical, multilayered acini with apicobasal polarity, as determined by staining with antibodies raised against apical marker GM130 and basal marker CD49f (Figure 1B). The immunoreactivity of inner cells with antibodies to the luminal marker protein MUC1 suggests that differentiation is multilineage. A key element of three-dimensional mammary differentiation is the death of cells forming the lumen of spherical acini. This was observed using calcein AM and ethidium homodimer 1 staining of unfixed three-dimensional cultures (Figure 1B). As with primary bipotent mammary epithelial progenitors [3], 184-*hTERT*-L9 acini exhibit squamous differentiation of the inner cells in three-dimensional Matrigel cultures, which is appreciable by the morphology of the inner cells stained with phalloidin (Figure 1B). The similarities and differences between 184-*hTERT*-L9 and MCF10A cells are shown in Additional file 4: Table S1.

### Defining the genes required for mammary epithelial cell growth under co-culture conditions

**Figure 3:**
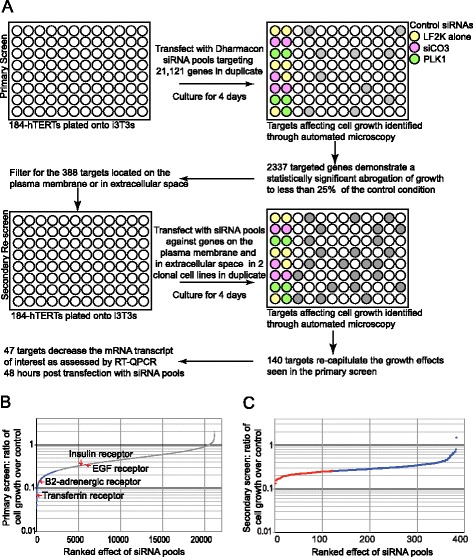
**High-throughput screen for genes regulating mammary epithelial cell growth. (A)** Summary of screen and follow-up assays leading to the identification of 47 candidate target genes. **(B)** Ranked distribution of growth effects for small interfering RNA (siRNA) pools utilized in the siRNA screen, normalized to the Lipofectamine 2000 (LF2K) reagent-alone control condition. Pooled siRNAs with a statistically significant effect that reduced growth to 25% or less of the control condition are highlighted in blue. **(C)** Ranked distribution of growth effects for siRNA pools utilized in the secondary screen targeting genes on the plasma membrane and in the extracellular space. Pooled siRNAs with a statistically significant effect in 184-*hTERT*-L9 and/or 184-*hTERT*-E11 cells that reduced growth to 25% or less of the control condition are highlighted in red. EGF, Epidermal growth factor; RT-QPCR, Quantitative RT-PCR.

To identify genes involved in the regulation of mammary epithelial cell growth, we used the 184-*hTERT*-L9 cells in a co-culture (fibroblasts and epithelial cells, defined medium without serum) genome-wide siRNA screen using 21,121 pools of siRNAs designed to the human genome (Dharmacon). The primary screen was used for initial hit identification, and multiple secondary screens focused on transmembrane and extracellular protein encoding genes were used to reconfirm and explore the genes implicated (Figure 3A). For the primary and secondary screening assays (Figure 3A), a layer of freshly irradiated murine NIH 3T3 cells was left to adhere for 24 hours prior to addition of a low density of 184-*hTERT*-L9 cells. After an additional 24 hours, these co-cultures were robotically transfected with the siRNA pools. Although two cell types were present in the assay, two features of the assay were used to bias the primary screen RNAi responses to the epithelial cells. First, the transfection conditions used for the epithelial cells were not capable of efficiently inducing RNAi transcript/protein knockdown in irradiated fibroblast cells (Additional file 5: Figures S3B and S3C), even with species-specific siRNAs (murine *β-actin* for NIH 3T3 cells, human *GAPDH* for IMR-90). The reduced capacity of irradiated versus nonirradiated fibroblasts to mediate RNAi was further demonstrated (Additional file 5: Figures S3A and S3D) using universally cell-lethal positive control siRNA (siTOX; Dharmacon) and siRNA to *PLK*, showing that irradiation abrogates the functional effects of these positive controls (Additional file 5: Figure S3D), although irradiated fibroblasts are still capable of executing cell death programs. Second, the siRNA library used is human-specific, whereas the fibroblasts are mouse-derived, so any siRNAs entering the cell would be of reduced effectiveness. It is important to note that, in order to confirm that growth effects were related to gene activity in the epithelial cells, all subsequent secondary screen revalidation experiments were carried out either in feeder-free conditions or with transfection into epithelial cells undertaken before placement onto a feeder layer. To quantify cell growth, we counted the number of nuclei per well 4 days after transfection with automated image recognition in duplicate assay plates [21]. In the co-culture conditions we used, changes in total nuclear count reflected the number of epithelial cells present, irrespective of the fixed number of nondividing feeder cells present per well (Additional file 6: Figure S2A).

**Figure 4:**
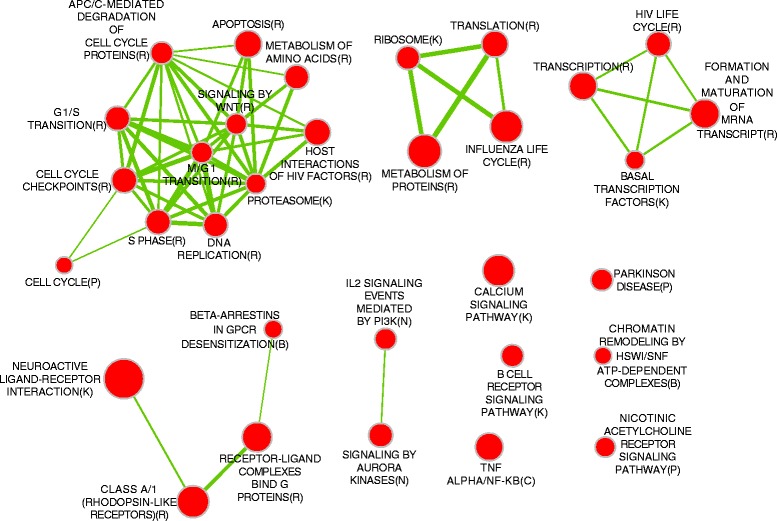
**Network analysis of genes important in epithelial cell growth.** Enrichment map of the top-ranking clusters of genes within the primary screen data that reduced growth to 25% or less of the control condition, as determined with the Reactome Functional Interactome plugin in Cytoscape v2.8.1. APC/C, Anaphase-promoting complex; GPCR, G protein–coupled receptor; IL2, Interleukin 2; NF-KB, Nuclear factor κB; PI3K, Phosphatidylinositol 3-kinase; TNF, Tumour necrosis factor.

The primary screen (two replicates per condition) resulted in the identification of 2,337 genes (of 21,121 (11.1%)) in which knockdown led to a statistically significant decrease (adjusted *P*-value <0.05 by Benjamini-Hochberg analysis) in cell growth to less than 25% of the control condition (Figure 3B and Additional file 7: Table S2A). We noted that several receptors for required components of the defined growth media were ranked in the top 50% (Figure 3B) of growth effects (insulin, epidermal growth factor (EGF), isoproterenol and transferrin). Enrichment map analysis using the Reactome Functional Interactome plugin in Cytoscape v2.8.1 [23,24] of the genes ranked by primary screen growth inhibition showed four statistically significant linked networks (Figure 4) and several singletons. As expected, the largest network implicated cell cycle functions; however, we also noticed a multinode network and several singleton nodes representing G protein–coupled receptor (GPCR)-mediated transmembrane signalling.

We therefore focused on further analysis of genes with subcellular location annotations [37] (Additional file 7: Table S2B) that indicated the presence of transmembrane, extracellular, and secreted proteins (388 genes with greater than 75% primary growth inhibition). To control for clonal cell line effects, we performed a secondary screen with these 388 genes (using a co-culture assay as primary screen) with both 184-*hTERT*-L9 and 184-*hTERT*-E11, a sister clonal cell line to 184-*hTERT*-L9 with identical growth characteristics, but with a different *hTERT* integration site. Of these 388 genes, 140 (36% of those retested) were considered reproducible in that they produced the same magnitude of growth reduction upon reassessment in the secondary screen (Figure 3C) in one or both clonal cell lines. We determined which of the 140 screen-reproducible siRNA pools were likely acting on target initially by quantitative RT-PCR (qRT-PCR) to determine which siRNA pools resulted in significant target transcript knockdown 48 hours after transfection into 184-*hTERT*-L9 cells. For this purpose, cells were grown in BPE as opposed to co-culture with irradiated feeder cells to reduce interfering mRNA signals. We found that 47 (33.6%) of 140 of the siRNA pools achieved statistically significant mRNA silencing (adjusted *P* < 0.05 by Benjamini-Hochberg analysis) (Additional file 7: Table S2C), and these were designated as the target genes for further study.

Additional validation was conducted as follows. (1) We deconvolved each siRNA pool to individual siRNAs and noted that in 20 (42.5%) of 47 of the siRNA sets, at least three of the individual siRNAs produced a statistically significant decrease (adjusted *P* < 0.05 by Benjamini-Hochberg multiple comparisons method) (Additional file 7: Table S2C) in growth within the two-dimensional co-culture assays. (2) We tested growth inhibition using independently designed lentiviral short-hairpin RNA (shRNA) constructs for each of the 47 target genes. One to three shRNA constructs from the GIPZ human lentiviral shRNA library (Dharmacon) were selected for analysis [38], and shRNAs were transduced in BPE-supplemented medium to maximize the potential of rescuing growth and formation of stable clones. Stably growing clones could not be isolated from 29 shRNAs covering 24 genes, suggesting the inability of these cells to grow upon gene silencing of these genes (verified by qRT-PCR in short-term culture; see Additional file 7: Table S2D), even with BPE present in the medium. Even among the stable clones derived, fibroblast-dependent growth was reduced. In total, 34 (72.3%) of 47 of the transmembrane and/or extracellular space protein encoding transcripts appeared to be required for epithelial cell growth, as determined by siRNA pool deconvolution and/or by knockdown with a second gene RNAi method (shRNA) (Additional file 6: Figure S2B and Additional file 7: Table S2C).

The increase and decrease in cell numbers observed in the primary and secondary screens likely resulted from a mix of proliferation (cell division) and apoptosis. To determine the relationship and relative contribution of these two factors, we quantified caspase 3/7 activity and proliferation simultaneously by using high-content live cell imaging (see IncuCyte ZOOM description in the Methods section). We observed that there was an inverse correlation (Additional file 8: Figure S4B (rank correlation −0.954) and Additional file 9: Figure S5 (rank correlation, −0.959)) between caspase activity and the rate of cell increase (determined as AUC over time). We verified that cell proliferation measured by high-content live cell imaging as AUC over time was positively correlated with the fraction of cells in S-phase, which we determined by EdU incorporation (Additional file 8: Figure S4A (rank correlation, 0.735)). The relationship between caspase activation and proliferation was similar, regardless of whether the epithelial cells were grown in feeder-free conditions (Additional file 8: Figure S4B) or with a feeder monolayer (Additional file 9: Figure S5) (feeder to no-feeder rank correlation, 0.892; *P* < 1 × 10^−5^ in randomization test). Taken together, these data show that the genes of greatest effect on cell growth tend to affect both proliferation (cell cycle) and caspase activation (apoptosis). Some exceptions were noted, such as the EDG (*LPAR3*) receptor, where the effect on proliferation was higher in rank than the degree of apoptosis.

Functional annotation clustering was performed using the DAVID Bioinformatics Database, with enrichment based upon the Gene Ontology biological process for the 47 genes required for 184-*hTERT*-L9 growth [39]. Enrichment scores were generated based upon the functional classification of these genes and denote the relatedness of a seemingly heterogeneous group of genes (Additional file 7: Table S2F). Notably, a number of guidance factors required for neuronal development are necessary for fibroblast-driven mammary epithelial cell growth [40,41]. Also enriched are clusters of genes involved in axon guidance, signal transduction through protein kinase cascades, intracellular ion homeostasis, GPCR signalling and cell migration (Additional file 10: Figure S6B). Additional file 10: Figure S6A displays the axon guidance pathway in which *SEMA3C*, and part of its receptor complex, *PLXNA2*, *ROBO3*, *EFNA4*, *NTN1* and *NTN2L* from our target gene list, are all highlighted. Axon guidance molecules are increasingly recognized to play a role in mammary gland development and breast tumourigenesis [42], and our data significantly strengthen this association. We considered the possibility that some of the 47 genes identified may have differential expression in the luminal and basal developmental lineages, and therefore we inspected flow-sorted RNA-seq libraries of mammary lumen and myoepithelium (three independent pairs from the RNA-seq library; see Additional file 11: Table S3 and discussion of NIH Canadian Roadmap Epigenomics mammary epithelial cell RNA-seq libraries in the Methods section). However, no statistically significant differences were observed (Holm-Bonferroni-adjusted *P*-values >0.05).

### Distinct requirements for transmembrane and/or extracellular genes in three-dimensional epithelial cell growth and differentiation

**Figure 5:**
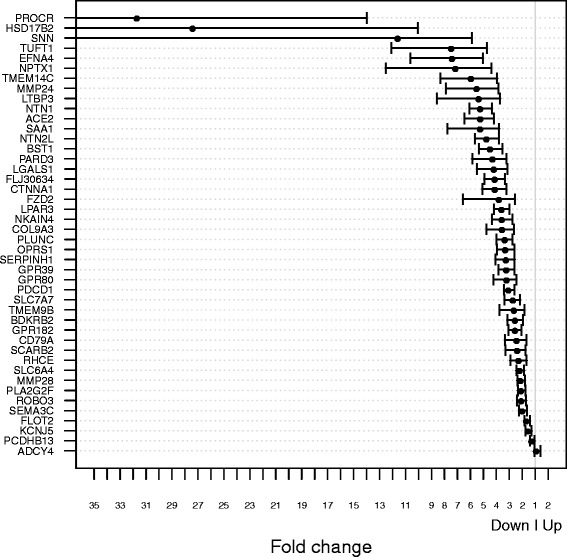
**Genes that modulate two-dimensional co-culture growth are also important in anchorage-independent three-dimensional growth.** Three-dimensional Matrigel culture assays were performed with 184-*hTERT*-L9 cells after they were transfected with small interfering RNA (siRNA) pools targeting the 47 genes required for mammary epithelial cell growth. The acini (with an acinus defined as a ball of 50 cells or more) formed per well were counted 8 days postplating, and surviving fraction estimates were generated in comparison to both transfection reagent-only and nonsilencing siRNA controls. Acini count fold changes are plotted as a rank relative to the control mean. Fold changes were calculated as mean number of acini when the gene was silenced in three-dimensional cultures divided by the mean number of acini when siRNA and transfection reagent controls were used in three-dimensional cultures. Error bars represent 95% confidence intervals (*n* = 4).

The response of mammary epithelial cells to mitogens and cellular signalling can differ dramatically when cells are grown on solid supports or feeder layers, as opposed to within a three-dimensional context embedded in extracellular matrix (Matrigel) [43,44]. Using short- and long-term cultures (with defined media and no BPE supplements) in Matrigel assays, we sought to determine which of the 47 gene transcripts identified as modulating epithelial cell growth in the co-culture assay were also required for three-dimensional growth and differentiation. First, using acinar formation in short-term culture as a growth measure (that is, counting three-dimensional structures formed after plating cells at low density, as in a two-dimensional colony-forming assay), we determined the requirement for each gene using siRNA knockdown. As siRNA transfection complexes cannot effectively penetrate Matrigel, we transfected cells in culture for 48 hours prior to harvesting and replating them in Matrigel [45]. We determined that siRNA silencing was effective for up to 10 days in Matrigel cultures of 184-*hTERT*-L9. The number of acini compared to a nontargeting siRNA control was enumerated after 8 days in culture (Figure 5). All but four genes (*ADCY4*, *PCDHB13*, *KCNJ5* and *FLOT2*) showed significant (*P* < 0.001 with Dunnett’s adjustment) reductions of growth in three dimensions, and three genes (*HSD17B2*, *PROCR* and *SNN*) showed more than tenfold reductions in the number of acini. We noted that, despite having the greatest effects on three-dimensional growth, *PROCR* (31.6-fold decrease in three dimensions; 95% confidence interval (CI), 14.0 to 163.8) and HSD17B2, a transmembrane-linked enzyme required for oestrogen and testosterone steroid biosynthesis (27.3-fold decrease in three dimensions; 95% CI, 10.0 to 163.9) were consistently in the third or fourth quartile of effects on two-dimensional growth in both the primary and secondary two-dimensional screens in comparison to the final 47 target genes. In all genes, the magnitude of growth modulation in two dimensions did not correlate with the magnitude of three-dimensional growth change (Additional file 7: Table S2C).

The requirements for three-dimensional growth are distinct from monolayer cultures, and the process of acinar formation can be disrupted for many reasons. In the second approach, we sought to determine if three-dimensional effects were limited solely to growth by looking at acinar formation after 21 days when polarization and lumen formation have occurred. For a 21-day culture, long-term RNAi is required, and we therefore examined shRNA stable lines in which growth could be rescued (for initial clone generation) by BPE media supplements. Among the three genes showing the greatest effects on acinar formation, *SNN*, *HSD17B2* and *PROCR* (Additional file 7: Table S2C), the shRNAs inhibited growth to a degree precluding derivation of stable clones for all but *PROCR*. The *PROCR* stable clone exhibited a 14.1-fold (99% CI, 7.16 to 28.57; RQ Manager software; Applied Biosystems, Foster City, CA, USA) compared to nontargeting controls (Additional file 7: Table S2D). When placed into a quantitative two-dimensional co-culture assay, growth was decreased 3.73-fold (95% CI, 3.38 to 4.16; Student’s *t*-test) in comparison to a stable cell line generated with a nontargeting shRNA construct.

**Figure 6:**
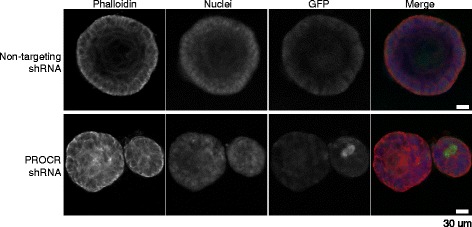
***PROCR*** **expression is necessary for cell organization and luminal clearance in three-dimensional culture.** 184-*hTERT*-L9 cell lines with stable integration of pGIPZ short-hairpin RNA (shRNA) lentiviral constructs against *PROCR* or a nontargeting control were seeded into three-dimensional Matrigel culture and fixed after 21 days of growth. Staining was performed with Alexa Fluor 546–conjugated phalloidin and DRAQ5 nuclear stain prior to imaging on a Nikon confocal laser scanning microscope. GFP, Green fluorescent protein.

After plating control and *PROCR*-knockdown clones in Matrigel, the number of acinar structures was decreased compared to the control nontargeting shRNA. Those acini that formed did so abnormally. These acini lacked the normal multilayered epithelial organization and did not form a hollow lumen, as seen in structures formed with the nontargeting shRNA (Figure 6), indicating a role for *PROCR* in mammary epithelial differentiation, organization and growth. To further explore the effects on three-dimensional differentiation and epithelial organization, we quantified the degree of polarization and lumen formation for *PROCR*, *EFNA4*, *LGALS1* and *NTN1*. Knockdown of all four genes resulted in increased disruption of GM130 localization (70% to −88% nonpolarized structures compared to 14% for nontargeting siRNA) (Additional file 1: Figure S7 and Additional file 2: Figures S8A and S8B); however, only *LGALS1* knockdown showed significant disruption of lumen formation (50% nonhollow compared to 8% to 17% for other genes and nontargeting siRNA) (Additional file 1: Figure S7 and Additional file 2: Figures S8A and S8B). *PROCR* was the only gene for which knockdown resulted in significant disruption of CD49f basal localization (100% structures disrupted) (Additional file 1: Figure S7 and Additional file 2: Figures S8A and S8B). Taken together, these results suggest that several of the genes identified may have roles in differentiation and development of multilayered mammary epithelium.

**Figure 7:**
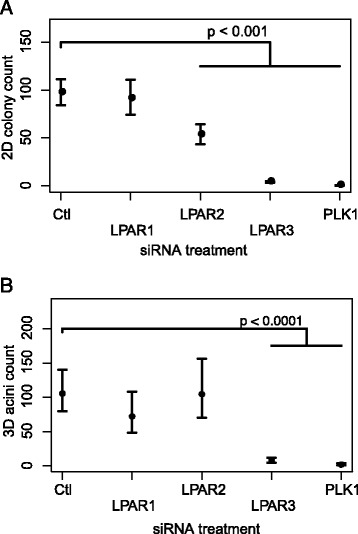
***LPAR3*** **, but not** ***LPAR1*** **or** ***LPAR2*** **, is necessary for two-dimensional and three-dimensional mammary growth.** Equal numbers of 184-*hTERT*-L9 cells transfected with small interfering RNA (siRNA) pools against three lysophosphatidic acid (LPA) receptors (*LPAR1*, *LPAR2* and *LPAR3*), transfection reagent alone and PLK1 (as a positive control) were plated in two-dimensional colony-forming assays (*n* = 3) **(A)** and three-dimensional Matrigel cultures (*n* = 4) **(B)**. Cells were counted after 8 days in culture. Error bars represent 95% confidence intervals.

Strikingly, both the two-dimensional and three-dimensional assays showed that several GPCRs appeared to be required for epithelial cell growth, including *LPAR3*, one of three major receptors for lysophosphatidic acid (LPA). In transgenic mouse models, overexpression of each of these individual LPA receptors (*LPAR1*, *LPAR2* and *LPAR3*) under the control of the mouse mammary tumour virus long terminal repeat (MMTV-LTR) promoter led to the formation of late-onset mammary carcinomas [46]. However, our screen of nontransformed human epithelium did not initially identify a role for either *LPAR1* or *LPAR2* in mammary epithelial cell growth. To verify this, we used siRNA pools to silence *LPAR1*, *LPAR2* and *LPAR3* expression in 184-*hTERT*-L9 cells prior to plating the cells in colony-forming assays. In keeping with the primary siRNA screen results, *LPAR3* was required for mammary epithelial cell growth, with moderate growth effects in the colony-forming assay identified when *LPAR2* was silenced (Figure 7A). Additionally, in three-dimensional culture conditions, silencing of *LPAR1* or *LPAR2* did not affect acinar formation, whereas we observed a complete abrogation of three-dimensional growth when *LPAR3* was silenced in 184-*hTERT* cells (Figure 7B).

### Requirement of screen-identified transmembrane and/or secreted genes for primary mammary progenitor cell growth

**Figure 8:**
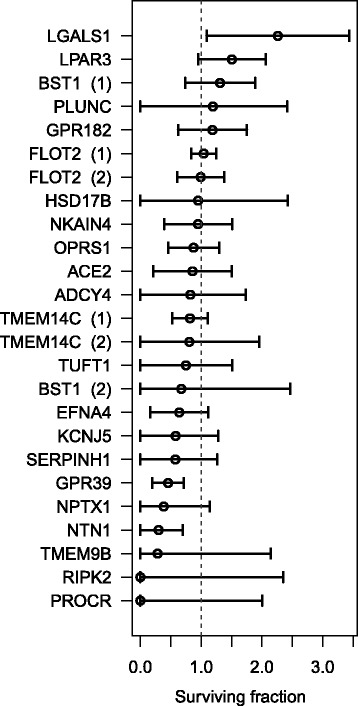
**Genes modulating 184-** ***hTERT*** **-L9 co-culture growth also affect primary human mammary epithelial cell growth.** Single-cell suspensions of dissociated human primary mammary cells were infected with individual short-hairpin RNA (shRNA) constructs targeting our screen-identified gene set prior to being plated in colony-forming assays. The fraction of surviving colonies after silencing of the target genes relative to a nontargeting shRNA control is plotted. Error bars represent 95% confidence intervals (*n* = 3 patient samples).

To address the question whether the growth dependency identified in the cell lines is mirrored in primary mammary epithelial cells, we tested 29 of the 47 target genes described above for their ability to diminish the *in vitro* growth of human primary mammary progenitor cells. The strategy employed was to transduce primary epithelium–enriched, but otherwise unsorted, cells obtained from dissociated reduction mammoplasties (see the Methods section for details) and to assay the luminal and basal cell epithelial lineages by scoring colony types in colony-forming assays [10,47-50]. All of the shRNA constructs tested produced statistically significant silencing of the mRNA transcript of interest in epithelial cells (Additional file 7: Table S2D). Transduction efficiency in unsorted primary tissue, as gauged by green fluorescent protein (GFP) expression from the pGIPZ lentiviral vector backbone, was 28.3% (95% CI, 20.2% to 36.1%). Three patient samples were evaluated in technical duplicates for each target gene to compare the effect of the target shRNA to that of a nonsilencing control shRNA. The total number of colonies produced from both luminal and myoepithelial progenitor cells was used to calculate the fraction of surviving colonies (see the Methods section for details) after silencing of the target genes (Figure 8). The high patient-to-patient variability in primary epithelial cell growth is a feature that constrained the quantitative assessment of primary cell growth effects; for example, *LPAR3* did not show a statistically significant growth difference. Nevertheless, the data showed a range of quantitative effects on colony formation of cells derived from primary epithelium, with more than two-thirds of the genes exhibiting a median decrease in colony formation and two genes showing a statistically significant decrease in surviving fraction (*GPR39* and *NTN1*; 2.19-fold and 3.33-fold decreases, respectively).

### Expression of screen-identified genes among breast cancer subtypes

**Figure 9:**
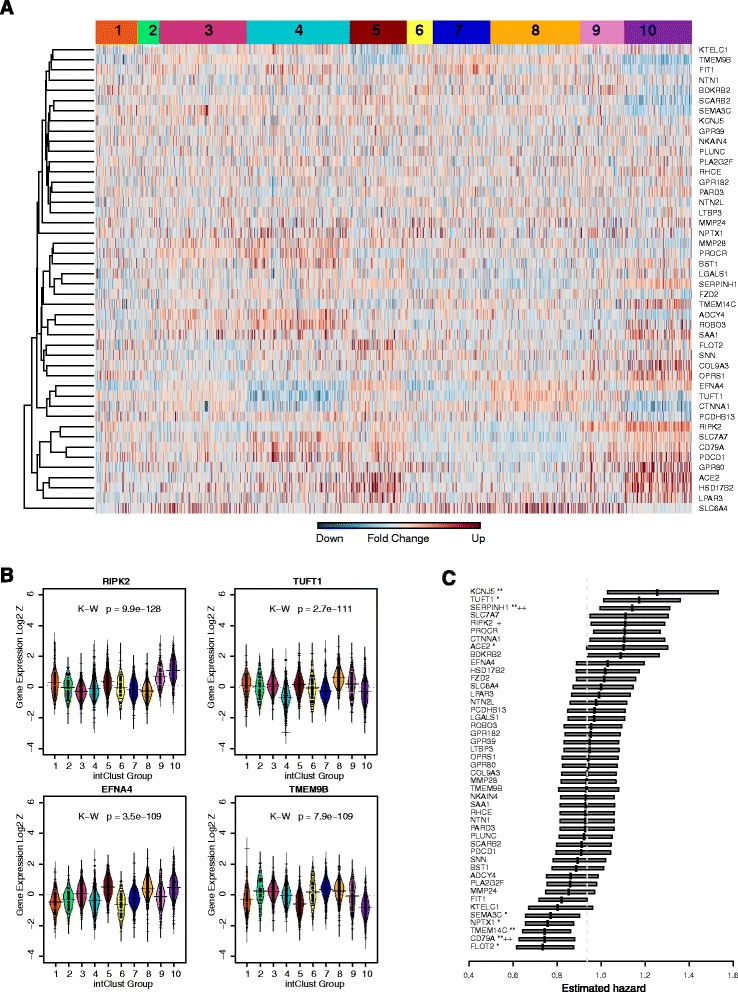
**Genes identified though screening are differentially expressed across breast cancer subtypes. (A)** Expression levels of the 47 genes found to be required for mammary epithelial cell growth are displayed on a heat map representing 1,998 breast cancer patients within the METABRIC dataset. Gene expression was determined on the Illumina HumanHT-12 Expression BeadChip array (Illumina, San Diego, CA, USA). Cases are clustered according to ten biological primary breast cancer subgroups described in Additional file 2: Figure S8 (also see [25]). **(B)** Beanplots depicting target gene expression in the ten biological primary breast cancer subgroups for *RIPK2*, *TUFT1*, *EFNA4* and *TMEM9B*. The individual observations are shown as small horizontal lines in a one-dimensional scatterplot with the estimated density of the distributions shown in colour and the average indicated by the long horizontal line. **(C)** Rank-ordered plot of hazard estimates and unadjusted 95% confidence intervals for high gene expression relative to low gene expression demonstrates differences in survival based upon the expression of the target genes. Gene targets are marked with identifiers for binarized expression variables showing adjusted *P*-values <0.05 (false discovery rate (FDR), 0.05) (denoted by ** or ++) and adjusted *P*-values <0.1 (FDR, 0.1) (denoted by * or +). Single asterisk indicates a significant finding after adjustment for multiple comparisons using the G-rho model test (ρ = 1). Single plus sign indicates a significant finding after adjustment for multiple comparisons using an omnibus test from a Cox model with biomarker and IntClust subtype and their interaction terms. K-W, Kruskal-Wallis test.

Genes regulating growth and differentiation are important components of the cell type landscape on which malignant transformation occurs in human cancers. One indication of this is the cosegregation of gene expression with biological subtypes of cancer. To address this question for the genes identified in relation to epithelial cell growth, we examined the distribution of transcript expression by subtype across 1,998 breast cancer patients [25] for whom mRNA expression has been measured, alongside other features of the genome and with clinical outcomes. Within the last year, the METABRIC study and related genomic landscapes have redefined the number of biologically distinct primary breast cancer subtypes [22,25,51-55]. Using these data and 10 recently defined biological primary breast cancer subgroups, we examined the expression levels of the 47 screen-identified genes (Figure 9A and B, Additional file 7: Table S2C and Additional file 12: Figure S9). The relationship to the five PAM50 breast cancer subgroups was also calculated (Additional file 13: Figure S10).

We examined each gene individually for subtype-specific expression. Significant differences in expression distribution were seen for 40 of the 47 genes across the 10 METABRIC datasets (Figure 9B and Additional file 12: Figure S9) (Kruskal-Wallis-adjusted *P*-value <0.05, Benjamini-Hochberg multiple-comparisons method) and for 39 of the 47 genes in the PAM50 groups (Additional file 13: Figure S10) [25]. Several genes (for example, *RIPK2*, *EFNA4* and *TMEM9B*) (Figure 9B, Additional file 12: Figure S9 and Additional file 13: Figure S10) showed differential expression in subtypes associated with high proliferation, such as IntClust 5 (predominantly HER2/ERBB2-amplified cancers) and IntClust 10 (predominantly basal expression type cancers). However, other breast cancer groups (IntClust 4, 6, 7 and 8, predominantly ER+ subtypes) (Figure 9A and B, Additional file 7: Table S2B) also showed significant enrichment for over- and underexpression of several genes. We noted that 21 of 47 genes required for 184-*hTERT* cells lie within chromosomal hotspots for copy number amplification (Additional file 7: Table S2C). All 47 genes have previously identified mutations in human tumours, and 18 of these genes have known mutations in breast tumour tissues (Additional file 7: Table S2C) [56,57].

To test the group as a whole, we also performed a randomization simulation study to assess the strength of association demonstrated by this set of 47 genes within the 10 IntClust subtypes. In 10,000 simulation runs, selecting 47 genes at random from among the 4,103 transmembrane/extracellular gene set, we found that 211 (0.21%) of the 10,000 of the random sets showed 40 or more genes to be significantly associated with the 10 METABRIC groups (null hypothesis simulation Kruskal-Wallis *P* = 0.021). For the PAM50 subtypes, 637 (0.637%) of 10,000 of the random sets of 47 genes showed 39 or more genes with an adjusted *P*-value <0.05 (null hypothesis simulation Kruskal-Wallis *P* = 0.064). Thus, the identified set of 47 genes is unlikely to be simply a randomly assembled set, suggesting that this gene set is enriched with regard to association with breast cancer subtype.

Finally, we asked whether expression differences in any of the 47 genes exhibit independent disease outcome associations (overall survival) in the 1,998-patient dataset. This was tested using two methods of multivariable analysis to determine if the prognostic significance of the gene expression was independent of that already carried by the breast cancer subtype (see the Methods section for details). The ranked proportional hazards with respect to overall survival (Figure 9C) for each gene showed a range of effect sizes. Two gene targets showed significant differences in survival within IntClust breast cancer subtype groups as assessed by Kaplan-Meier G-rho-stratified analysis and by Cox proportional hazards analysis, after adjustment for multiple comparisons using the method of Benjamini and Hochberg [26]. Elevated *CD79A* expression showed improved survival for IntClusts 8 and 10 (Additional file 14: Figure S11). Elevated *SERPINH1* expression showed poorer survival for IntClusts 6, 9 and 10. An additional two gene targets showed significant differences as assessed by the Kaplan-Meier G-rho-stratified analysis, after adjustment for multiple comparisons. Elevated *KCNJ5* expression showed poorer survival for IntClusts 4 and 8. Elevated *TMEM14C* expression showed improved survival for IntClust 4.

## Discussion

The extracellular factors and transmembrane signals that regulate mammary epithelial growth and differentiation remain poorly understood. In part, this is due to a lack of methods for systematic, genome-wide, genetic and functional interrogation of genes in relation to mammary epithelial growth and differentiation. Although genome-wide functional screens using RNAi methods have proven successful in many similar instances (for example, see [58,59]), these have mostly been undertaken with transformed, somatically mutated epithelial cell types, where key extracellular interactions that modulate growth cannot be recapitulated. Important features of the endogenous milieu, such as growth stimulation by fibroblast stroma have been undersampled as a consequence. To overcome this, we isolated and characterized a cloned, diploid, nontransformed mammary epithelial cell line, 184-*hTERT*-L9, which retains both fibroblast growth dependence and the capacity to differentiate in three-dimensional growth conditions. We used this cell line in a genome-wide RNAi screen to identify, in a systematic manner, genes required for mammary progenitor cell growth and differentiation.

The 184-*hTERT*-L9 clone described here is derived from primary mammary epithelium immortalized by *hTERT* transfection after limited initial passages. The clonal line retains important properties, such as fibroblast-dependent growth and the ability to differentiate in three-dimensional cultures, and is diploid and nontransformed.

Fibroblasts are known to possess an instructive role in regulating mammary epithelial cells in normal development and oncogenesis [6,60,61]. More specifically, the *in vitro* growth of bipotent progenitor cells is reliant upon the presence of fibroblasts as feeder cells. The 184-*hTERT*-L9 cells mimic the growth of bipotent progenitor cells when plated with increasing densities of irradiated NIH 3T3 feeder cells in a well-defined colony-forming assay. This is not observed in epithelial cell lines with genomic aberrations and/or additional adaptation events, such as MCF10A cells (Figure 2) and transformed epithelial cells derived from malignancies. Thus, 184-*hTERT*-L9 cells provided a genomically characterized model system, also amenable to RNAi transfection and image-based, high-content screening, whereby we could replicate *in vitro* the fibroblast-dependent growth environment of mammary progenitor cells in order to investigate the signalling pathways involved in regulating mammary epithelial and progenitor cell growth.

Among the 21,121 siRNA pools tested in the primary screen, 2,337 demonstrated statistically significant abrogation of growth to less than 25% of the control condition. This was a stringent selection criterion, given that knockdown of receptors for two of the four defined medium components (insulin, EGF, transferrin and isoproterenol) did not achieve this level of growth inhibition. Surprisingly, GPCR and associated signalling proteins were also found amongst this list, suggesting an underappreciated role of this class of receptors within mammary gland biology.

To identify novel external regulators and signal transducers, we focused our in-depth analysis on cell surface and secreted genes. After rescreening including an independently cloned sister cell to assess reproducibility, and after qPCR assessment for on-target siRNA activity, we identified 47 transmembrane genes for follow-up examination by deconvolution and by assaying growth using independently targeted shRNA constructs (summarized in Table 1). Moreover, we quantified the relative influence of proliferation and apoptosis for each gene, which indicated a general inverse correlation between these two functions.

**Table 1:** **Relative effects of the 47 target genes in two-dimensional and three-dimensional cultures** ^**a**^

**Gene name**	**RefSeq ID**	**Relative rank, primary screen (** ***N*** **= 47)**	**Median relative rank, secondary screen (** ***N*** **= 47)**	**Relative rank, three-dimensional growth assay (** ***N*** **= 44)**	**Three-dimensional acinar formation, fold decrease (siRNA)**	**shRNA knockdown effect on clone**
*ACE2*	NM_021804	22	30	11	5.169	Lethal
*ADCY4*	NM_139427	44	17	44	0.878	Lethal
*BDKRB2*	NM_000623	28	44	31	2.484	Growth postselection
*BST1*	NM_004334	7	6	14	4.406	Lethal
*CD79A*	NM_001783	13	16	33	2.366	Growth postselection
*COL9A3*	NM_001853	16	4	22	3.489	Lethal
*CTNNA1*	NM_001903	37	29	18	4.047	Lethal
*EFNAA*	NM_005227	38	37	5	7.338	Growth postselection
*FIT1*	NM_203402	4	22	No data	No data	Growth postselection
*FL_J30634*	NM_153014	15	30	17	4.055	Growth postselection
*FLOT2*	NM_004475	23	31	41	1.618	Lethal
*FZD2*	NM_001466	6	25	19	3.7126	Lethal
*GPR182*	NM_007264	9	20	32	2.483	Growth postselection
*GPR39*	NM_001508	10	22	26	3.159	Growth postselection
*GPR80*	NM_080818	32	14	27	3.137	Lethal
*HSD17B2*	NM_002153	27	38	2	27.328	Lethal
*KCNJ5*	NM_000890	3	35	42	1.494	Growth postselection
*KTELC1*	NM_020231	8	17	No data	No data	Growth postselection
*LGALS1*	NM_002305	36	7	16	4.120	Growth postselection
*LPAR3*	NM_012152	1	5	20	3.530	Growth postselection
*LTBP3*	NM_021070	39	28	9	5.291	Growth postselection
*MMP24*	NM_00690	40	5	8	5.447	Growth postselection
*MMP28*	NM_024302	18	25	37	2.075	Lethal
*NKAIN4*	NM_152864	20	9	21	3.505	Lethal
*NPTX1*	NM_002522	45	18	6	7.082	Lethal
*NTN1*	NM_004822	47	22	10	5.175	Growth postselection
*NTN2L*	NM_006181	5	30	13	4.691	Growth postselection
*OPRS1*	NM_005866	29	26	24	3.243	Lethal
*PARD3*	NM_019619	30	31	15	4.221	Lethal
*PCDHB13*	NM_018933	46	27	43	1.214	Growth postselection
*PDCD1*	NM_00518	21	36	28	2.997	Growth postselection
*PLA2G2F*	NM_022819	19	22	38	2.028	Lethal
*PLUNC*	NM_016583	31	30	23	3.280	Growth postselection
*PROCR*	NM_006404	35	36	1	31.637	Growth postselection
*RHCE*	NM_020485	42	31	35	2.209	Lethal
*RIPK2*	NM_003821	41	23	No data	No data	Lethal
*ROBO3*	NM_022370	11	35	39	2.012	Growth postselection
*SAA1*	NM_000331	14	21	12	5.167	Lethal
*SCARB2*	NM_005506	34	9	34	2.317	Lethal
*SEMA3C*	NM_006379	12	25	40	1.936	Growth postselection
*SERPINH1*	NM_001235	26	32	25	3.187	Lethal
*SLC6A4*	NM_001045	17	29	36	2.142	Growth postselection
*SNN*	NM_003498	24	30	3	11.523	Lethal
*TMEM14C*	NM_016462	33	32	7	5.884	Lethal
*TMEM9B*	NM_020644	43	23	30	2.556	Lethal
*TUFT1*	NM_020127	25	20	4	7.399	Lethal
		/47	/47	/44		

^a^The relative rank of target genes in the primary small interfering RNA (siRNA) screen, the median relative rank in the secondary siRNA screen and the relative rank in the three-dimensional acinar growth assay are displayed. The fold decrease in structure formation for each target gene is displayed for each siRNA relative to the control condition. The effect of infection with an independent lentiviral short-hairpin (shRNA) clone against each target gene is shown after selection with puromycin (resistance marker within lentiviral construct) as a binary effect (lethal or growth postselection).

Although these 47 genes have diverse functions, they are strikingly enriched for both GPCRs (*LPAR3*, *FZD2*, *ADMR*, *BDKRB2*, *GPR39*, *GPR80* and *GPR182*) (noted in the primary screen) and axonal guidance molecules (*SEMA3C*, *PLXNA2*, *ROBO3*, *EFNA4*, *NTN1* and *NTN2L*). For many of these genes, we provide the first description of a role in growth regulation or mammary biology.

To better understand the roles of these genes in growth and differentiation (reviewed in [62]), we assessed the requirement of the 47 validated target genes for growth in three-dimensional culture. The silencing of all but four of the genes (*ADCY4*, *PCDHB13*, *KCNJ5* and *FLOT2*) decreased three-dimensional acinar formation to a level comparable to that seen with PLK1 silencing (which is essential for mitosis). Intriguingly, we have shown that *LPAR3* is required for two-dimensional and three-dimensional mammary growth. Given the established importance of *LPAR1*, *LPAR2* and *LPAR3* in mammary tumourigenesis, we wanted to confirm that *LPAR1* and *LPAR2* are indeed irrelevant for growth of normal epithelium (as determined in the primary genetic screen). Colony formation in two-dimensional assays and three-dimensional acinar formation within Matrigel still occur upon silencing of *LPAR1* and *LPAR2*, suggesting that they are not required for the growth of normal, nontransformed epithelial cells. *LPAR3* has a greater binding affinity for 2-acyl-LPA with unsaturated fatty acids, whereas *LPAR1* and *LPAR2* are more responsive to saturated acyl chains [63]. With responsiveness to a similar ligand, it is possible that compensatory redundancy exists between *LPAR1* and *LPAR2*.

Some of the genes identified as regulating growth in two dimensions also affected differentiation when epithelial cells were grown in three-dimensions, with *SNN*, *HSD17B2* and *PROCR* showing greater than tenfold reduction in acinar formation. The requirement for long-term cultures (21 days) and the lethality of the shRNAs for *SNN* and *HSD17B2* precluded analysis of these two genes; however, reduction of *PROCR* in long-term cultures was associated with absence of lumen formation and disorganized epithelial growth. We quantified the relative effects on epithelial organization and lumen formation and observed that disordered differentiation was also present for two axonal pathfinding associated genes, *EFNA4* and *NTN1*, and for *LGALS1* (Additional file 1: Figure S7, Figure 8), with differential effects on polarization and epithelial organization. *PROCR* has been implicated as a receptor for protease-cleaved substrates in breast cancer migration [64] and as a marker of colony-forming cells in malignant cell lines [65]. Here we show for the first time, to our knowledge, a role in growth and differentiation of primary breast epithelium. Loss of *NTN1* causes disorganization in the terminal end buds, an effect that is proposed to occur through the loss of cellular adhesions [66]. It has also been shown that implantation of *NTN1*-secreting pellets into mammary glands during pregnancy increases the number of alveolar structures that develop [67]. In the present study, we show a role for *NTN1* in both luminal and bipotent progenitor cell growth in that it was required for colony formation in our *in vitro* assays.

Finally, genes required for growth and differentiation are often implicated in tumourigenesis. In this study, we identified a subset of genes that have not previously been implicated in mammary gland growth or development. We sought to determine if expression of these genes correlated with any of the breast cancer subtypes. Significant, nonrandom differences in expression distribution across the 10 METABRIC datasets was seen for 40 of the 47 genes, with several genes (for example, *RIPK2*, *EFNA4* and *TMEM9B*) differentially expressed in breast cancer subtypes with high proliferation. Furthermore, we were able to demonstrate independent prognostic significance for *CD79A* (with elevated expression improving survival in two of the ten METABRIC subtypes) and *SERPINH1* (with elevated expression decreasing survival in three of the ten METABRIC subtypes). The possible roles of these genes in the tumour subtypes studied requires future functional studies in representative models; however, it is notable that all of the genes studied here are accessible by virtue of solubility or membrane location, making them a practical choice for intervention.

## Conclusions

This work shows for the first time, to our knowledge, the diversity of transmembrane signals and/or proteins required for the growth of nontransformed mammary epithelial cells in the physiological state where extracellular signals from fibroblasts are required. In doing so, we demonstrate the functional requirement of several transmembrane and extracellular proteins in normal mammary growth in multiple well-accepted models of *in vitro* mammary physiology. Taken further, these proteins were differentially associated with breast cancer subtypes, which were examined in 1,998 patients, indicating that these proteins may be associated with the biology of breast cancer subtypes. This is of particular note, as the location of these proteins makes them amenable to therapeutic intervention.

## Additional files

Additional file 1: Figure S7. Target gene expression is necessary for normal acinar formation in three-dimensional culture. 184-*hTERT*-L9 cell lines with stable integration of pGIPZ shRNA lentiviral constructs against EFNA4, LGALS1, NTN1, PROCR or a nontargeting control were seeded into three-dimensional Matrigel culture and fixed after 21 days of growth. Staining was performed with Alexa Fluor 546–conjugated phalloidin and DRAQ5 nuclear stain prior to imaging on a Nikon confocal laser scanning microscope. For E-cadherin, the percentage of structures that were hollow versus filled was quantified for each condition, with LGALS1 showing a significant increase in the percentage of filled structures relative to the control. For CD49f (marker of basal polarity), the percentage of structures that were polarized versus nonpolarized was quantified, with PROCR showing a complete reversal of polarization relative to the control and other siRNAs. For GM130 (a marker of apical polarity), the percentage of structures that were polarized versus nonpolarized was quantified, with all of the siRNAs tested showing a decrease in polarized structures relative to the nontargeting siRNA control. Examples of the patterns scored in each case are shown in the upper panel series below the table (white indicates higher fluorescence intensity). The middle panel represents the same images with the greyscale inverted to better reveal the antibody pattern (dark indicates higher fluorescence intensity). The presence of apoptotic cells in the siNTN1 structures is shown in the bottom panel (dark reddish-brown indicates caspase by immunohistochemistry).
Additional file 2: Figure S8. Epithelial organization is disrupted in three-dimensional culture with silencing of target genes. **(A)** 184-*hTERT*-L9 cell lines with stable integration of pGIPZ shRNA lentiviral constructs against EFNA4, LGALS1, NTN1, PROCR or a nontargeting control were seeded into three-dimensional Matrigel culture and fixed after 21 days of growth. Staining was performed with Alexa Fluor 546–conjugated phalloidin and DRAQ5 nuclear stain prior to imaging on a Nikon confocal laser scanning microscope. Magnified views of representative structures for each condition are presented for CD49f, E-cadherin and GM130 staining. **(B)** The same images shown in **(A)** are depicted with greyscale inverted for visual clarity.
Additional file 3: Figure S1. 184-*hTERT*-L9 cells are a cytogenetically normal cloned human mammary epithelial cell line. **(A)** Southern blots of HindIII- and BglII-digested DNA with a cDNA probe targeting the neomycin resistance gene present on a lentiviral construct used to immortalize the cells showing one band per digestion, suggesting a single integration site and a single population of cells after cloning in three representative cell lines (184-*hTERT*-L9, 184-*hTERT*-L5 and 184-*hTERT*-L2). **(B)** Immunohistochemistry of formalin-fixed, paraffin-embedded 184-*hTERT*-L9 cell blocks shows ubiquitous expression of keratin 5/6, epidermal growth factor receptor, the epithelial cell marker E-cadherin and wild-type p53. There is no detectable expression of oestrogen receptor α (ERα) or human epidermal growth factor receptor 2 (HER2). **(C)** Western blots of clonal 184-*hTERT*-L9, 184-*hTERT*-L5, 184-*hTERT*-L9 and primary unsorted mammary epithelial cells (HMECs) with antibodies raised against β-actin, keratin 18, keratin 5, p63 and p16. **(D)** Array-based comparative genomic hybridization was performed using the Affymetrix GeneChip SNP 6.0 array (Affymetrix, Santa Clara, CA, USA). Log_2_ ratios of signal intensity for 184-*hTERT* cell lines compared to normal human female reference DNA are plotted in relation to chromosomal position.
Additional file 4: Table S1. Contrasting properties of the 184-*hTERT*-L9 [20] and MCF10A [17,68,69] cell lines.
Additional file 5: Figure S3. Irradiated fibroblasts are not susceptible to siRNA-mediated RNA interference. **(A)** Irradiated murine NIH 3T3 cells do not efficiently mediate RNAi with a universal cell-lethal siRNA. Irradiated NIH 3T3 cells at a density of 30,000 cells/cm^2^ were transfected with increasing concentrations of transfection reagent. After 48 hours, live-dead discrimination was performed (calcein AM/ethidium homodimer 1) and enumerated using the IN Cell Analyzer and IN Cell Developer software (GE Healthcare Bio-Sciences). The percentage of viable cells was determined in comparison to nontransfected control wells. Error bars represent standard deviation (*n* = 2). **(B)** Irradiated fibroblasts do not show protein target knockdown. Irradiated NIH 3T3 cells at a density of 10,000 cells/cm^2^ were transfected with 50 nM pooled siRNAs targeting β-actin complexed with increasing transfection reagent concentrations. After 96 hours, β-actin (green) and lamin C (red) levels were detected using the LI-COR Odyssey imaging system (LI-COR Biotechnology, Lincoln, NE, USA). **(C)** Irradiated fibroblasts do not show protein target knockdown. Irradiated IMR-90 human fibroblasts at a density of 10,000 cells/cm^2^ were transfected with 50 nM pooled siRNAs targeting human *GAPDH*, with transfection reagent alone (Lipofectamine 2000) and with a nontargeting siRNA (siCon3) as controls. After 72 hours, Western blotting was performed with both *GAPDH* and then β-actin (control). **(D)** The cell killing abilities of siPLK1 and siTOX were compared in irradiated and nonirradiated NIH 3T3 cells. Cell number was assessed (confluence over time) on the IncuCyte ZOOM live cell microscope for irradiated and nonirradiated NIH 3T3 cells with increasing concentrations of siTOX and mouse siPLK1. Nontransfected cells (Lipofectamine 2000) were included as the control. Cell number was not above starting and/or control conditions for the irradiated fibroblasts, as expected, given their arrested state. Application of siTOX to the irradiated cells had no significant effect on irradiated cell viability (no difference from controls; Dunnett-adjusted *P* > 0.05). In contrast, in the nonirradiated cells, both siPLK1 and siTOX result in cell killing compared to the control (Dunnett-adjusted *P* < 0.02).
Additional file 6: Figure S2. Optimization of genome-wide siRNA screening parameters and external validation of results. **(A)** Total nuclear count accurately reflects the 184-*hTERT* count in the co-culture screening assay. Comparison of total nuclear count and GFP-positive count in co-cultures treated with a siRNA library targeting cell cycle genes showed that the total count can be used as a surrogate for the GFP-positive count. Three thousand irradiated NIH 3T3 cells were plated with four hundred 184-*hTERT*-GFP cells in 96-well plates prior to transfection with 0.3 μl of Lipofectamine 2000 reagent and 30 nM of siRNA per well. Twenty-one fields of view were acquired after 5 days of growth using a 10× lens objective on the IN Cell Analyzer. The GFP-negative count was obtained by subtracting the GFP-positive count (green diamond) from the total nuclear count (red square) and represents the number of irradiated fibroblasts present in each well (blue triangle). **(B)** Deconvolution of siRNA pools and RNA interference with lentiviral shRNA constructs externally validates different subsets of genes. Genes listed in blue and purple within the Venn diagram represent genes with which 184-*hTERT* cells infected with an independently designed lentiviral shRNA construct were unable to grow in culture after puromycin was applied to the cells as a means of selection for stable integration of the lentiviral construct. Genes listed in red and purple within the Venn diagram represent genes with which knockdown with three or four of the individual siRNAs comprising the original siRNA pool led to a statistically significant decrease in cell growth (adjusted *P* < 0.05 by Benjamini-Hochberg multiple-comparisons method).
Additional file 7: Table S2.
**(A)** Ranked growth effect of the 21,121 genes evaluated in the primary siRNA screen. **(B)** Ranked growth effect of the 388 extracellular and plasma membrane genes evaluated in the secondary siRNA screen. **(C)** Relative effects of the 47 target genes in two-dimensional and three-dimensional cultures, as well as their status in the METABRIC and COSMIC databases. **(D)** Relative transcript levels after lentiviral shRNA silencing in 184-*hTERT*-L9 cells. **(E)** RT-qPCR primers and corresponding Roche Universal ProbeLibrary (Roche Applied Science, Penzberg, Germany) probe number utilized for RT-qPCR. **(F)** Functional enrichment scores for 47 target genes. Functional annotation clustering was performed on the 47 genes required for 184-*hTERT*-L9 cell growth using the Gene Functional Classification tool in the DAVID Bioinformatics Database [39]. The data used to derive the annotation clustering heat maps in Additional file 10: Figure S6 are listed.
Additional file 8: Figure S4. Relative contribution of proliferation and apoptosis of target genes in two-dimensional culture. **(A)** Cell proliferation as measured by AUC over time by high-content imaging is positively correlated with the fraction of cells in S-phase, determined by EdU incorporation. EdU incorporation was performed in 24-well plates containing 184-*hTERT*-L9 cells stably transfected with NucLight Red. Cells were transfected with 30 nM siRNA 24 hours after plating and labelled with EdU 68 hours posttransfection. Labelling was detected with the Click-iT EdU Alexa Fluor 488 Flow Cytometry Assay Kit and analysed by flow cytometry. **(B)** Cell proliferation (AUC over time, median value in red) and relative apoptosis (caspase-3/7 activity, median value in green) in the target genes by rank-ordered effects on proliferation display an inverse correlation. 184-*hTERT* cells stably infected with NucLight Red were plated without feeder cells and transfected 24 hours later with 30 nM of siRNA to the respective target genes. CellPlayer Caspase-3/7 reagent was used to mark apoptotic cells after an additional 24 hours. Proliferation was measured every 4 hours for 84 hours, with respective AUCs for serial measurements calculated for each condition for red (proliferating) or dual-labelled (apoptotic) cells.
Additional file 9: Figure S5
**Relative contributions of proliferation and apoptosis of target genes in two-dimensional culture is unchanged by the presence of feeder cells.** Cell proliferation (AUC over time, median value in red) and relative apoptosis (caspase-3/7 activity, median value in green) in the target genes by rank-ordered effects on proliferation display an inverse correlation. 184-*hTERT* cells stably infected with NucLight Red were plated with irradiated NIH 3T3 feeder cells and transfected 24 hours later with 30 nM of siRNA to the respective target genes. CellPlayer Caspase-3/7 reagent was used to mark apoptotic cells after an additional 24 hours. Proliferation was measured every 4 hours for 84 hours, with respective AUCs for serial measurements calculated for each condition for red (proliferating) or dual labelled (apoptotic) cells. Genes with the greatest effect on growth affect both proliferation and apoptosis. (PNG 148 kb)
Additional file 10: Figure S6. Genes identified through screening and functionally related. **(A)** Functional annotation clustering was performed on the 47 genes required for 184-*hTERT*-L9 cell growth using the Gene Functional Classification tool in the DAVID Bioinformatics Database [39]. The cluster of genes involved in axon guidance are highlighted by red stars on the Kyoto Encyclopedia of Genes and Genomes (KEGG) human axon guidance pathway [40,41]. **(B)** Functional annotation clustering was performed on the 47 genes required for 184-*hTERT*-L9 cell growth using the Gene Functional Classification tool in the DAVID Bioinformatics Database [39]. Enrichment clusters are depicted for genes with their corresponding associated Gene Ontology biological process terms. Positive gene term associations are represented in green, and currently unreported gene term associations are represented in black.
Additional file 11: Table S3. Target genes are not differentially expressed in freshly sorted RNA-seq libraries of enriched luminal versus enriched myoepithelial cells taken from human reduction mammoplasty samples. Data from three independent paired libraries of mammary luminal and myoepithelium for each target gene are shown along with luminal:basal expression ratios and the *P*-values calculated with the Wilcoxon and Student’s *t* tests and the Holm-Bonferroni adjustment. No statistically significant differences (Holm-Bonferroni-adjusted *P*-values >0.05) were observed.
Additional file 12: Figure S9. Beanplots depicting target gene expression in the ten biological primary breast cancer subgroups. The individual observations are shown as small horizontal lines in a one-dimensional scatterplot with the estimated density of the distributions shown in colour and the average indicated by the long horizontal line.
Additional file 13: Figure S10. Beanplots depicting target gene expression in the PAM50 breast cancer subgroups. The individual observations are shown as small horizontal lines in a one-dimensional scatterplot with the estimated density of the distributions shown in colour and the average indicated by the long horizontal line.
Additional file 14: Figure S11. Independent disease outcome associations are present with select target genes. Individual genes shown in Figure 9C are depicted with significant survival differences with low (green) versus high (red) gene expression for both integrative cluster (IntClust) breast cancer subtype groups and PAM50 subtypes. Associations were assessed by Kaplan-Meier G-rho-stratified analysis and by Cox proportional hazards analysis, after adjustment for multiple comparisons using the method of Benjamini and Hochberg [26]. CD79A expression shows improved survival for IntClusts 8 and 10 and HER2. KCNJ5 expression shows poorer survival for IntClusts 4 and 8. SERPINH1 expression shows poorer survival for IntClusts 6, 9 and 10. TMEM14C expression shows improved survival for IntClust 4 and luminal B.

## Abbreviations

AIC: Akaike information criterion
AUC: Area under the curve
BPE: Bovine pituitary extract
CI: Confidence interval
DAPI: 4′,6-diamidino-2-phenylindole
E-cad: E-cadherin
EdU: 5-ethynyl-2′-deoxyuridine
ERα: Oestrogen receptor α
EpCAM: Epithelial cell adhesion molecule
FACS: Fluorescence-activated cell sorting
FDR: False discovery rate
FISH: Fluorescence *in situ* hybridization
GFP: Green fluorescent protein
GPCR: G protein–coupled receptor
HER2: Human epidermal growth factor receptor 2
IntClust: Integrative cluster
MEBM: Mammary epithelial cell basal medium
MEGM: Mammary epithelial cell growth medium
MOI: Multiplicity of infection
RNAi: RNA interference
shRNA: Short-hairpin RNA
siRNA: Small interfering RNA
