# Supplementary figures and images for: A co-culture genome-wide RNAi screen with mammary epithelial cells reveals transmembrane signals required for growth and differentiation
Source: Breast Cancer Res. 2015 Jan 9;17:4. doi: 10.1186/s13058-014-0510-y (PMC4322558; doi:10.1186/s13058-014-0510-y)

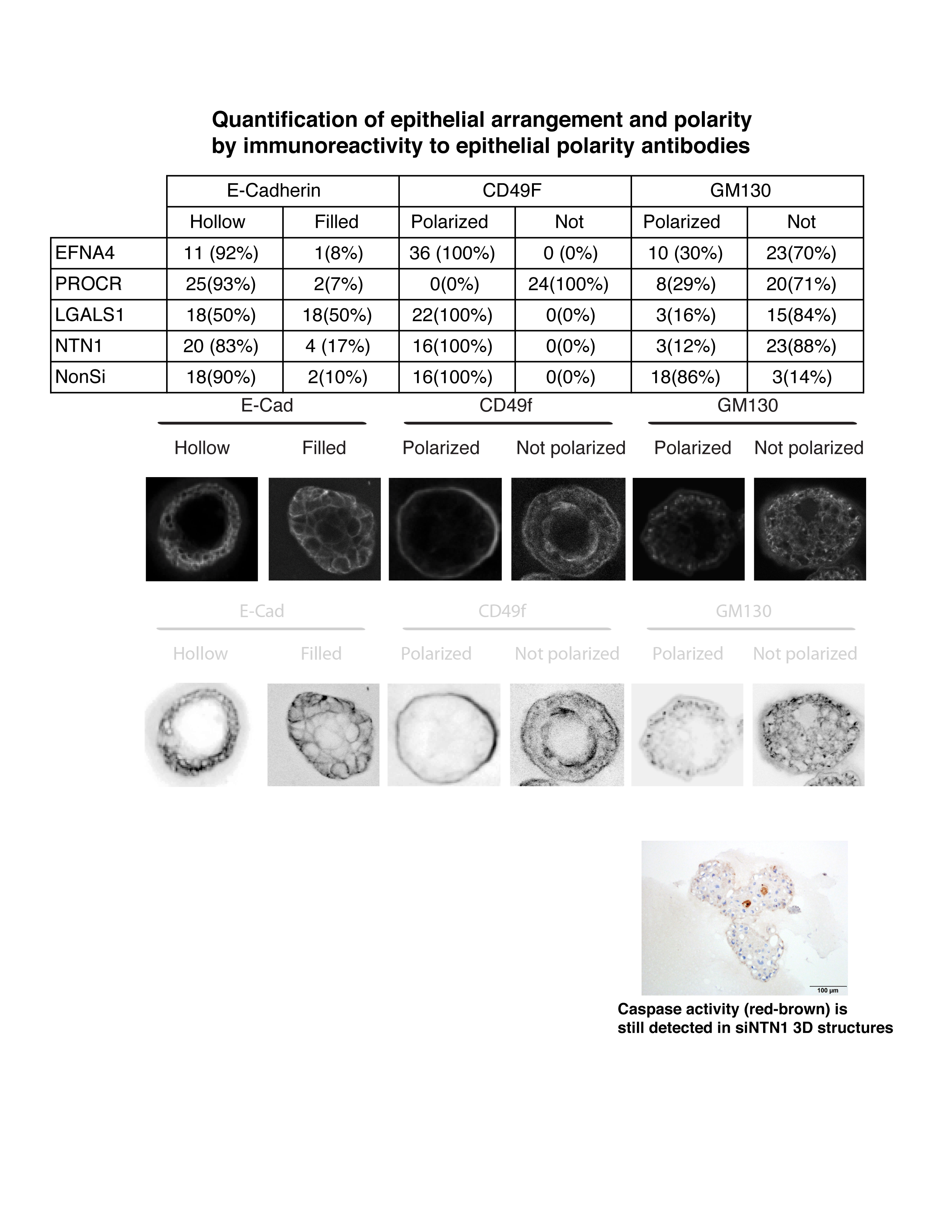

Supplement: Additional file 1: Figure S7. — Target gene expression is necessary for normal acinar formation in three-dimensional culture. 184-hTERT-L9 cell lines with stable integration of pGIPZ shRNA lentiviral constructs against EFNA4, LGALS1, NTN1, PROCR or a nontargeting control were seeded into three-dimensional Matrigel culture and fixed after 21 days of growth. Staining was performed with Alexa Fluor 546–conjugated phalloidin and DRAQ5 nuclear stain prior to imaging on a Nikon confocal laser scanning microscope. For E-cadherin, the percentage of structures that were hollow versus filled was quantified for each condition, with LGALS1 showing a significant increase in the percentage of filled structures relative to the control. For CD49f (marker of basal polarity), the percentage of structures that were polarized versus nonpolarized was quantified, with PROCR showing a complete reversal of polarization relative to the control and other siRNAs. For GM130 (a marker of apical polarity), the percentage of structures that were polarized versus nonpolarized was quantified, with all of the siRNAs tested showing a decrease in polarized structures relative to the nontargeting siRNA control. Examples of the patterns scored in each case are shown in the upper panel series below the table (white indicates higher fluorescence intensity). The middle panel represents the same images with the greyscale inverted to better reveal the antibody pattern (dark indicates higher fluorescence intensity). The presence of apoptotic cells in the siNTN1 structures is shown in the bottom panel (dark reddish-brown indicates caspase by immunohistochemistry). [file 13058_2014_510_MOESM1_ESM.png]

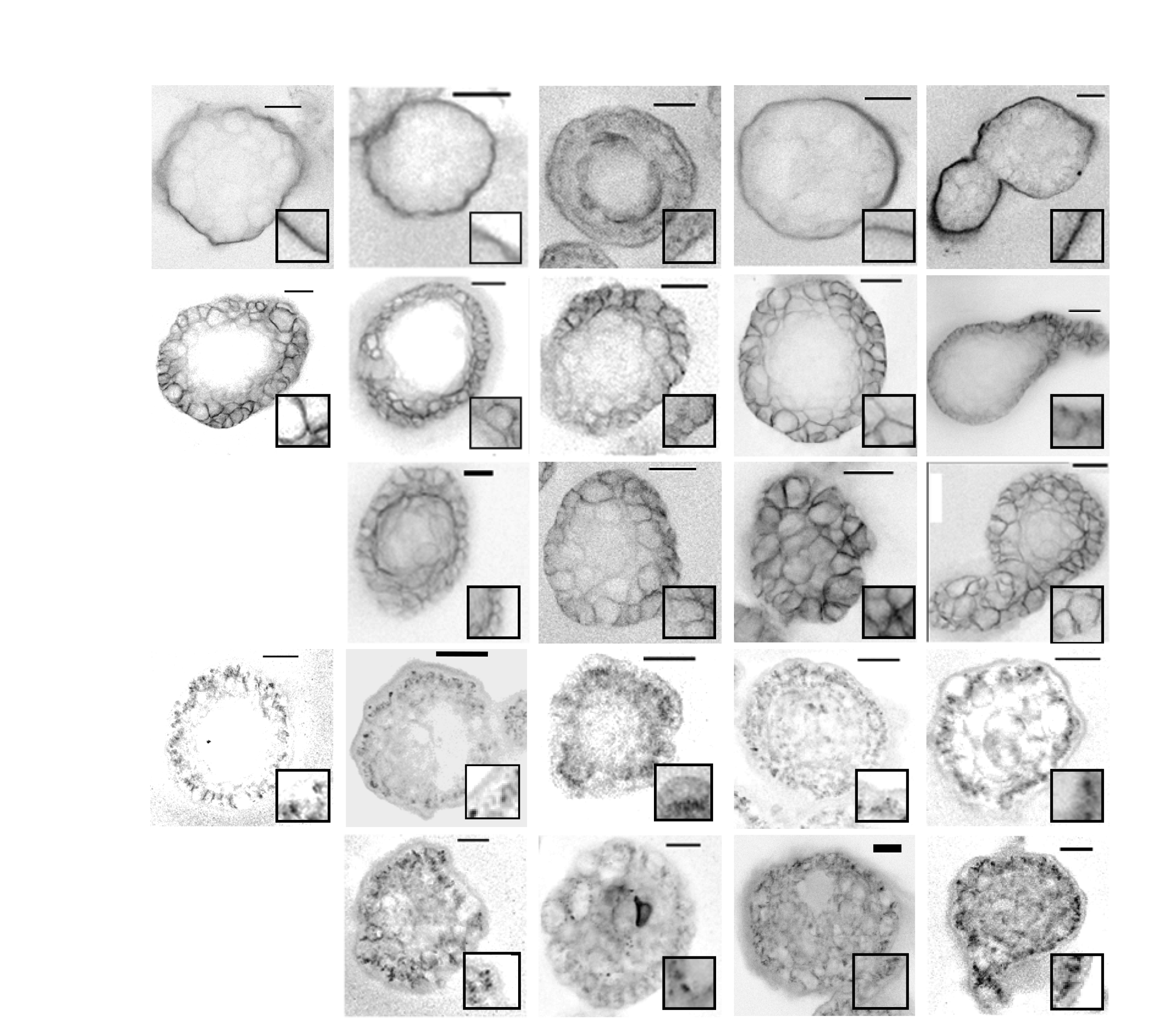

Supplement: Additional file 2: Figure S8. — Epithelial organization is disrupted in three-dimensional culture with silencing of target genes. (A) 184-hTERT-L9 cell lines with stable integration of pGIPZ shRNA lentiviral constructs against EFNA4, LGALS1, NTN1, PROCR or a nontargeting control were seeded into three-dimensional Matrigel culture and fixed after 21 days of growth. Staining was performed with Alexa Fluor 546–conjugated phalloidin and DRAQ5 nuclear stain prior to imaging on a Nikon confocal laser scanning microscope. Magnified views of representative structures for each condition are presented for CD49f, E-cadherin and GM130 staining. (B) The same images shown in (A) are depicted with greyscale inverted for visual clarity. [file 13058_2014_510_MOESM2_ESM.zip › 3501832231099773_add9.png]

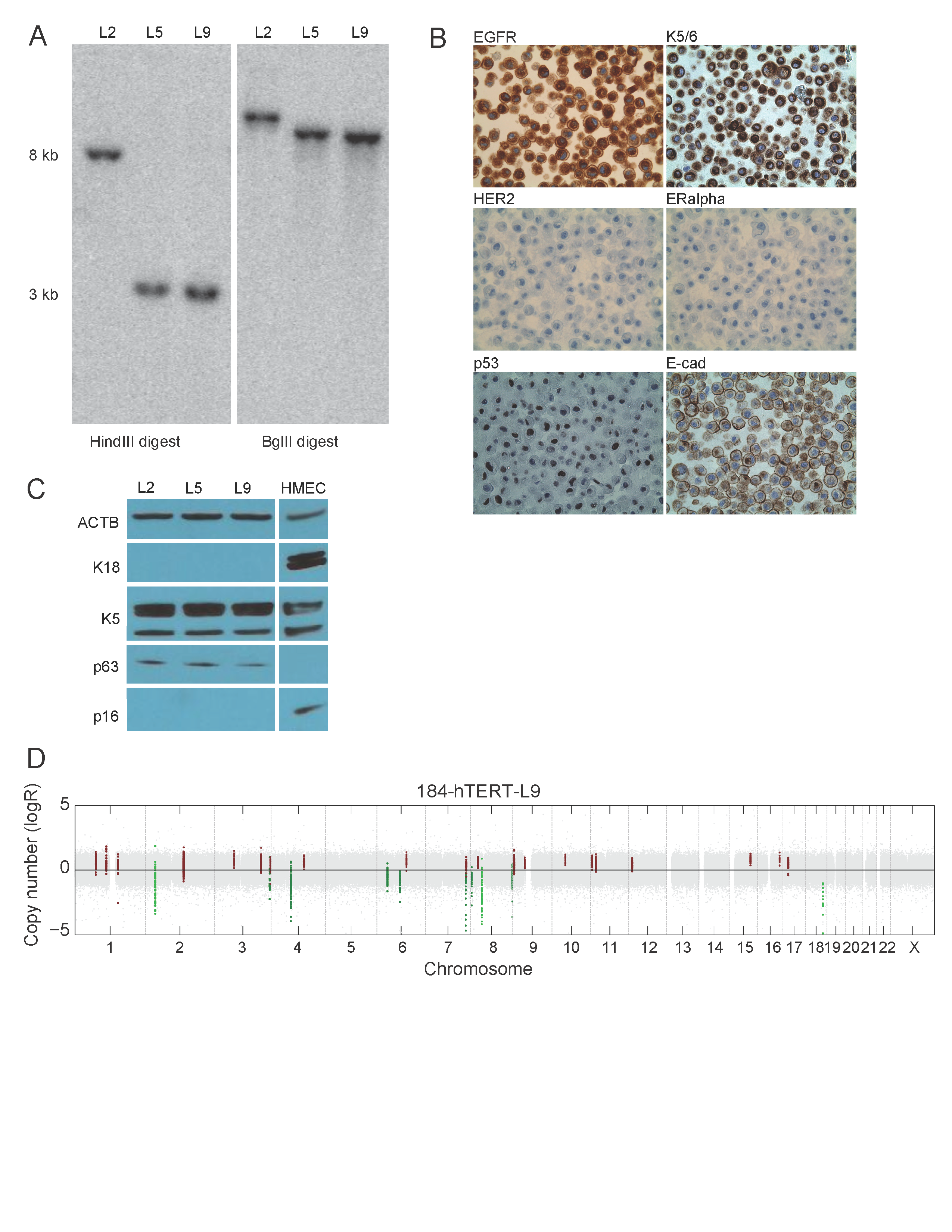

Supplement: Additional file 3: Figure S1. — 184-hTERT-L9 cells are a cytogenetically normal cloned human mammary epithelial cell line. (A) Southern blots of HindIII- and BglII-digested DNA with a cDNA probe targeting the neomycin resistance gene present on a lentiviral construct used to immortalize the cells showing one band per digestion, suggesting a single integration site and a single population of cells after cloning in three representative cell lines (184-hTERT-L9, 184-hTERT-L5 and 184-hTERT-L2). (B) Immunohistochemistry of formalin-fixed, paraffin-embedded 184-hTERT-L9 cell blocks shows ubiquitous expression of keratin 5/6, epidermal growth factor receptor, the epithelial cell marker E-cadherin and wild-type p53. There is no detectable expression of oestrogen receptor α (ERα) or human epidermal growth factor receptor 2 (HER2). (C) Western blots of clonal 184-hTERT-L9, 184-hTERT-L5, 184-hTERT-L9 and primary unsorted mammary epithelial cells (HMECs) with antibodies raised against β-actin, keratin 18, keratin 5, p63 and p16. (D) Array-based comparative genomic hybridization was performed using the Affymetrix GeneChip SNP 6.0 array (Affymetrix, Santa Clara, CA, USA). Log2 ratios of signal intensity for 184-hTERT cell lines compared to normal human female reference DNA are plotted in relation to chromosomal position. [file 13058_2014_510_MOESM3_ESM.png]

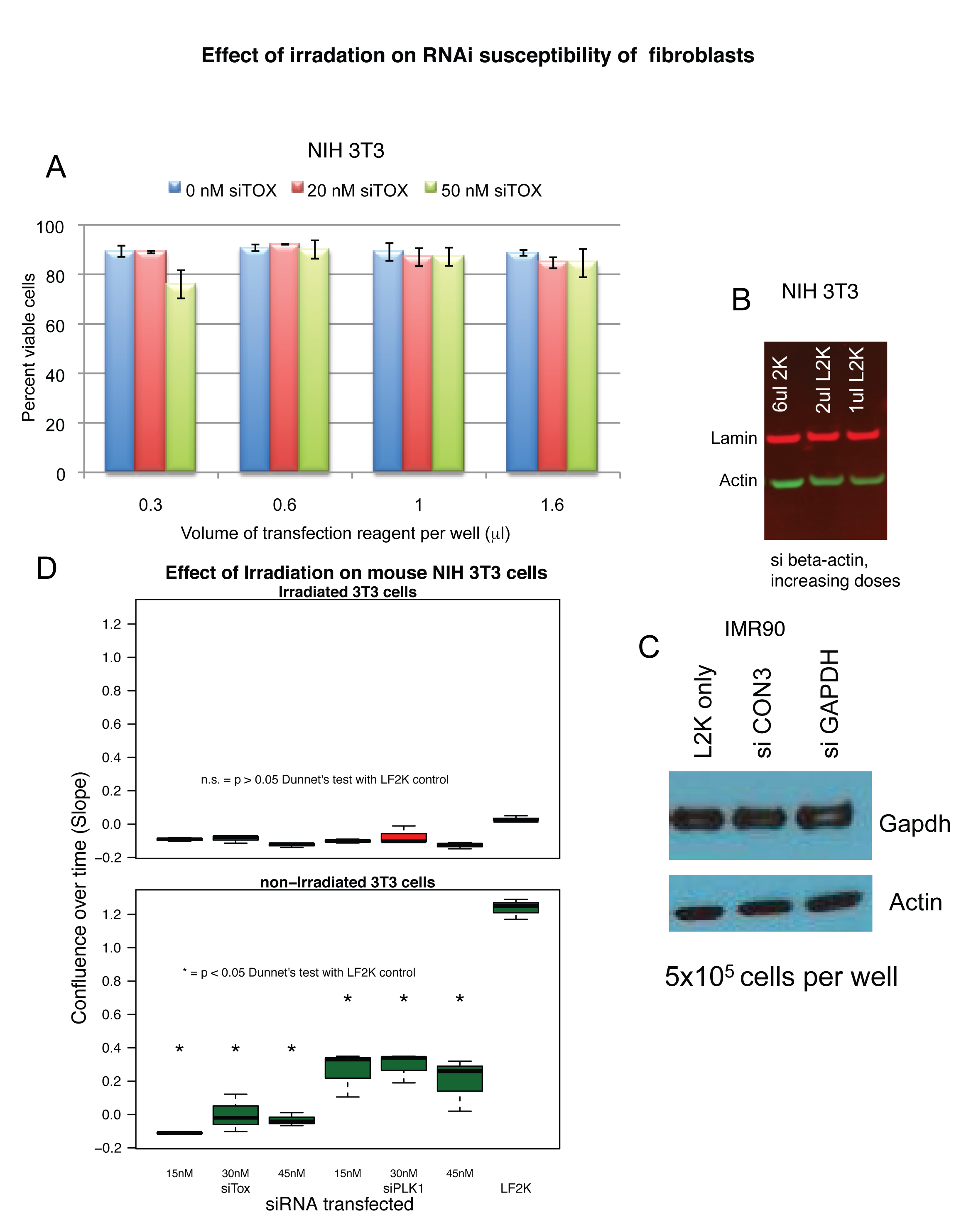

Supplement: Additional file 5: Figure S3. — Irradiated fibroblasts are not susceptible to siRNA-mediated RNA interference. (A) Irradiated murine NIH 3T3 cells do not efficiently mediate RNAi with a universal cell-lethal siRNA. Irradiated NIH 3T3 cells at a density of 30,000 cells/cm2 were transfected with increasing concentrations of transfection reagent. After 48 hours, live-dead discrimination was performed (calcein AM/ethidium homodimer 1) and enumerated using the IN Cell Analyzer and IN Cell Developer software (GE Healthcare Bio-Sciences). The percentage of viable cells was determined in comparison to nontransfected control wells. Error bars represent standard deviation (n = 2). (B) Irradiated fibroblasts do not show protein target knockdown. Irradiated NIH 3T3 cells at a density of 10,000 cells/cm2 were transfected with 50 nM pooled siRNAs targeting β-actin complexed with increasing transfection reagent concentrations. After 96 hours, β-actin (green) and lamin C (red) levels were detected using the LI-COR Odyssey imaging system (LI-COR Biotechnology, Lincoln, NE, USA). (C) Irradiated fibroblasts do not show protein target knockdown. Irradiated IMR-90 human fibroblasts at a density of 10,000 cells/cm2 were transfected with 50 nM pooled siRNAs targeting human GAPDH, with transfection reagent alone (Lipofectamine 2000) and with a nontargeting siRNA (siCon3) as controls. After 72 hours, Western blotting was performed with both GAPDH and then β-actin (control). (D) The cell killing abilities of siPLK1 and siTOX were compared in irradiated and nonirradiated NIH 3T3 cells. Cell number was assessed (confluence over time) on the IncuCyte ZOOM live cell microscope for irradiated and nonirradiated NIH 3T3 cells with increasing concentrations of siTOX and mouse siPLK1. Nontransfected cells (Lipofectamine 2000) were included as the control. Cell number was not above starting and/or control conditions for the irradiated fibroblasts, as expected, given their arrested state. Application of siTOX to the irradiated cells [file 13058_2014_510_MOESM5_ESM.png]

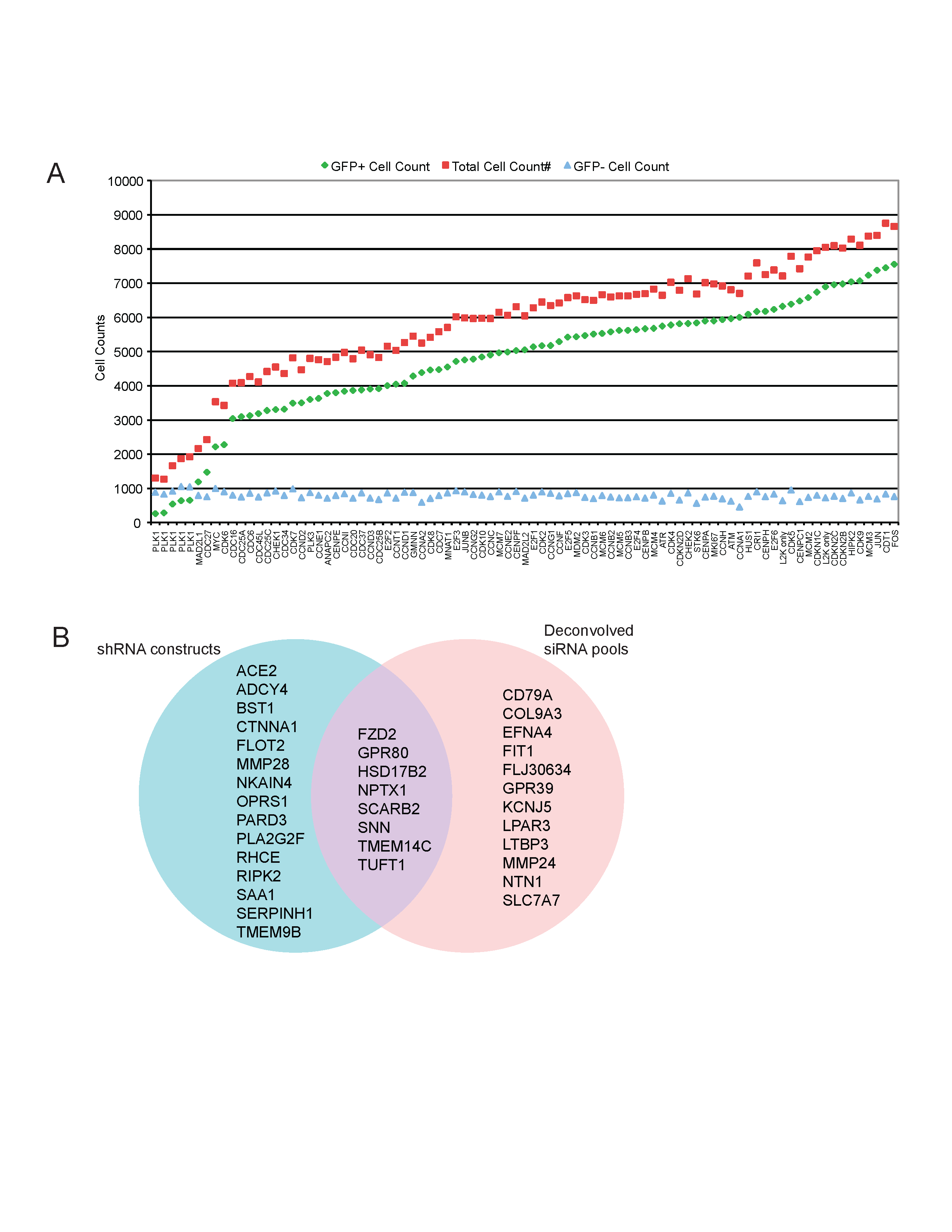

Supplement: Additional file 6: Figure S2. — Optimization of genome-wide siRNA screening parameters and external validation of results. (A) Total nuclear count accurately reflects the 184-hTERT count in the co-culture screening assay. Comparison of total nuclear count and GFP-positive count in co-cultures treated with a siRNA library targeting cell cycle genes showed that the total count can be used as a surrogate for the GFP-positive count. Three thousand irradiated NIH 3T3 cells were plated with four hundred 184-hTERT-GFP cells in 96-well plates prior to transfection with 0.3 μl of Lipofectamine 2000 reagent and 30 nM of siRNA per well. Twenty-one fields of view were acquired after 5 days of growth using a 10× lens objective on the IN Cell Analyzer. The GFP-negative count was obtained by subtracting the GFP-positive count (green diamond) from the total nuclear count (red square) and represents the number of irradiated fibroblasts present in each well (blue triangle). (B) Deconvolution of siRNA pools and RNA interference with lentiviral shRNA constructs externally validates different subsets of genes. Genes listed in blue and purple within the Venn diagram represent genes with which 184-hTERT cells infected with an independently designed lentiviral shRNA construct were unable to grow in culture after puromycin was applied to the cells as a means of selection for stable integration of the lentiviral construct. Genes listed in red and purple within the Venn diagram represent genes with which knockdown with three or four of the individual siRNAs comprising the original siRNA pool led to a statistically significant decrease in cell growth (adjusted P < 0.05 by Benjamini-Hochberg multiple-comparisons method). [file 13058_2014_510_MOESM6_ESM.png]

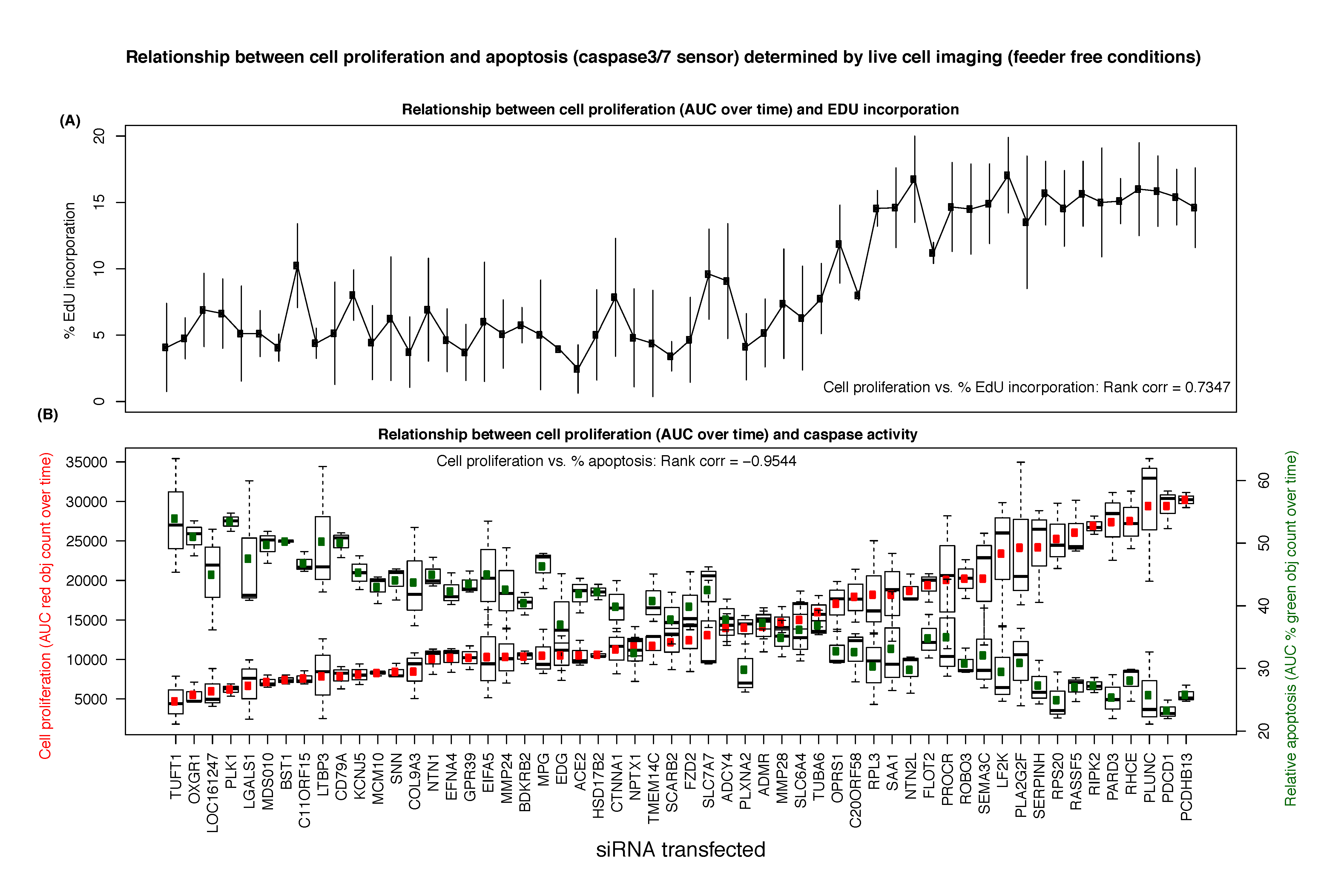

Supplement: Additional file 8: Figure S4. — Relative contribution of proliferation and apoptosis of target genes in two-dimensional culture. (A) Cell proliferation as measured by AUC over time by high-content imaging is positively correlated with the fraction of cells in S-phase, determined by EdU incorporation. EdU incorporation was performed in 24-well plates containing 184-hTERT-L9 cells stably transfected with NucLight Red. Cells were transfected with 30 nM siRNA 24 hours after plating and labelled with EdU 68 hours posttransfection. Labelling was detected with the Click-iT EdU Alexa Fluor 488 Flow Cytometry Assay Kit and analysed by flow cytometry. (B) Cell proliferation (AUC over time, median value in red) and relative apoptosis (caspase-3/7 activity, median value in green) in the target genes by rank-ordered effects on proliferation display an inverse correlation. 184-hTERT cells stably infected with NucLight Red were plated without feeder cells and transfected 24 hours later with 30 nM of siRNA to the respective target genes. CellPlayer Caspase-3/7 reagent was used to mark apoptotic cells after an additional 24 hours. Proliferation was measured every 4 hours for 84 hours, with respective AUCs for serial measurements calculated for each condition for red (proliferating) or dual-labelled (apoptotic) cells. [file 13058_2014_510_MOESM8_ESM.png]

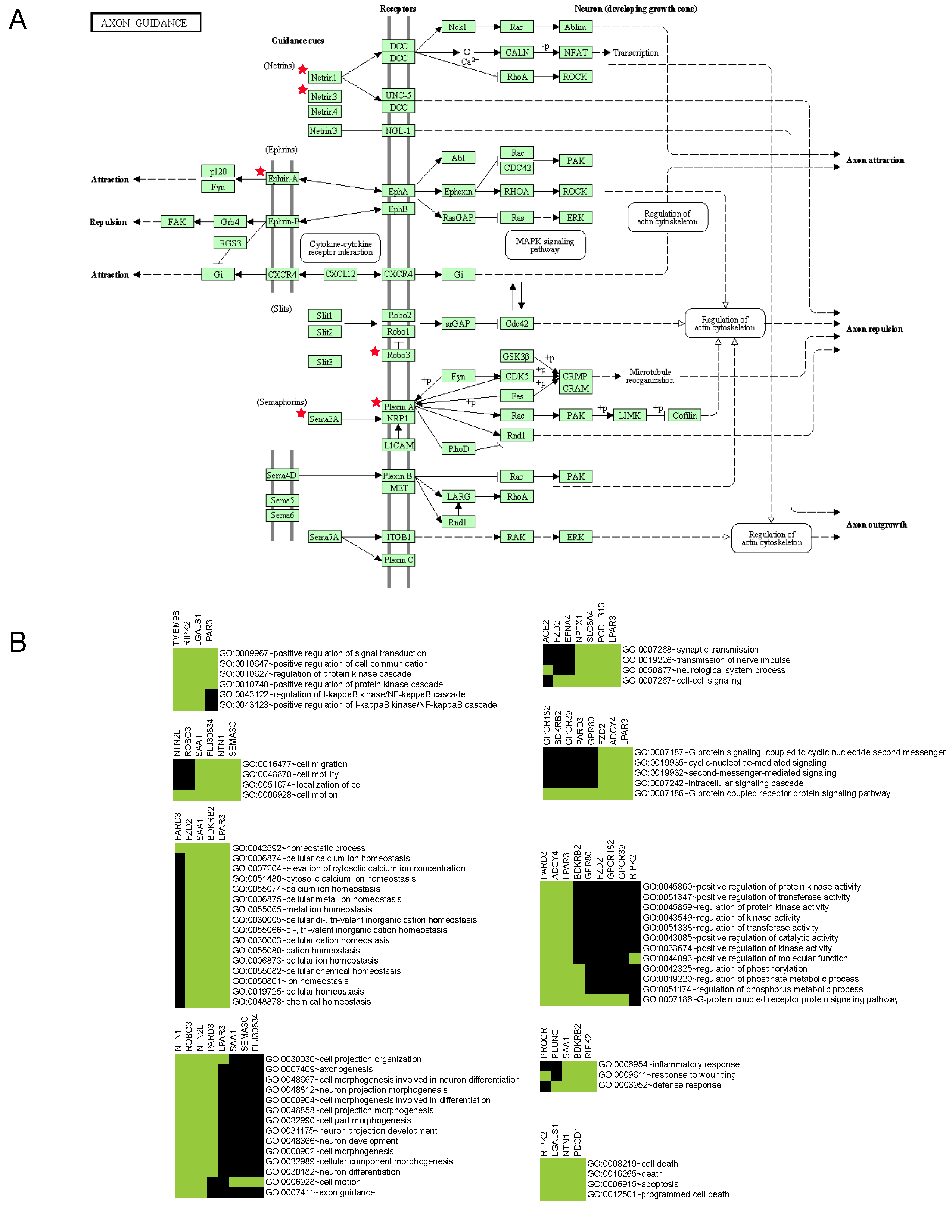

Supplement: Additional file 10: Figure S6. — Genes identified through screening and functionally related. (A) Functional annotation clustering was performed on the 47 genes required for 184-hTERT-L9 cell growth using the Gene Functional Classification tool in the DAVID Bioinformatics Database [39]. The cluster of genes involved in axon guidance are highlighted by red stars on the Kyoto Encyclopedia of Genes and Genomes (KEGG) human axon guidance pathway [40,41]. (B) Functional annotation clustering was performed on the 47 genes required for 184-hTERT-L9 cell growth using the Gene Functional Classification tool in the DAVID Bioinformatics Database [39]. Enrichment clusters are depicted for genes with their corresponding associated Gene Ontology biological process terms. Positive gene term associations are represented in green, and currently unreported gene term associations are represented in black. [file 13058_2014_510_MOESM10_ESM.png]
