# Supplementary material for: A co-culture genome-wide RNAi screen with mammary epithelial cells reveals transmembrane signals required for growth and differentiation
Source: Breast Cancer Res. 2015 Jan 9;17:4. doi: 10.1186/s13058-014-0510-y (PMC4322558; doi:10.1186/s13058-014-0510-y)

Epithelial organisation revealed by immunofluorescence with polarity markers in 3-D colonies

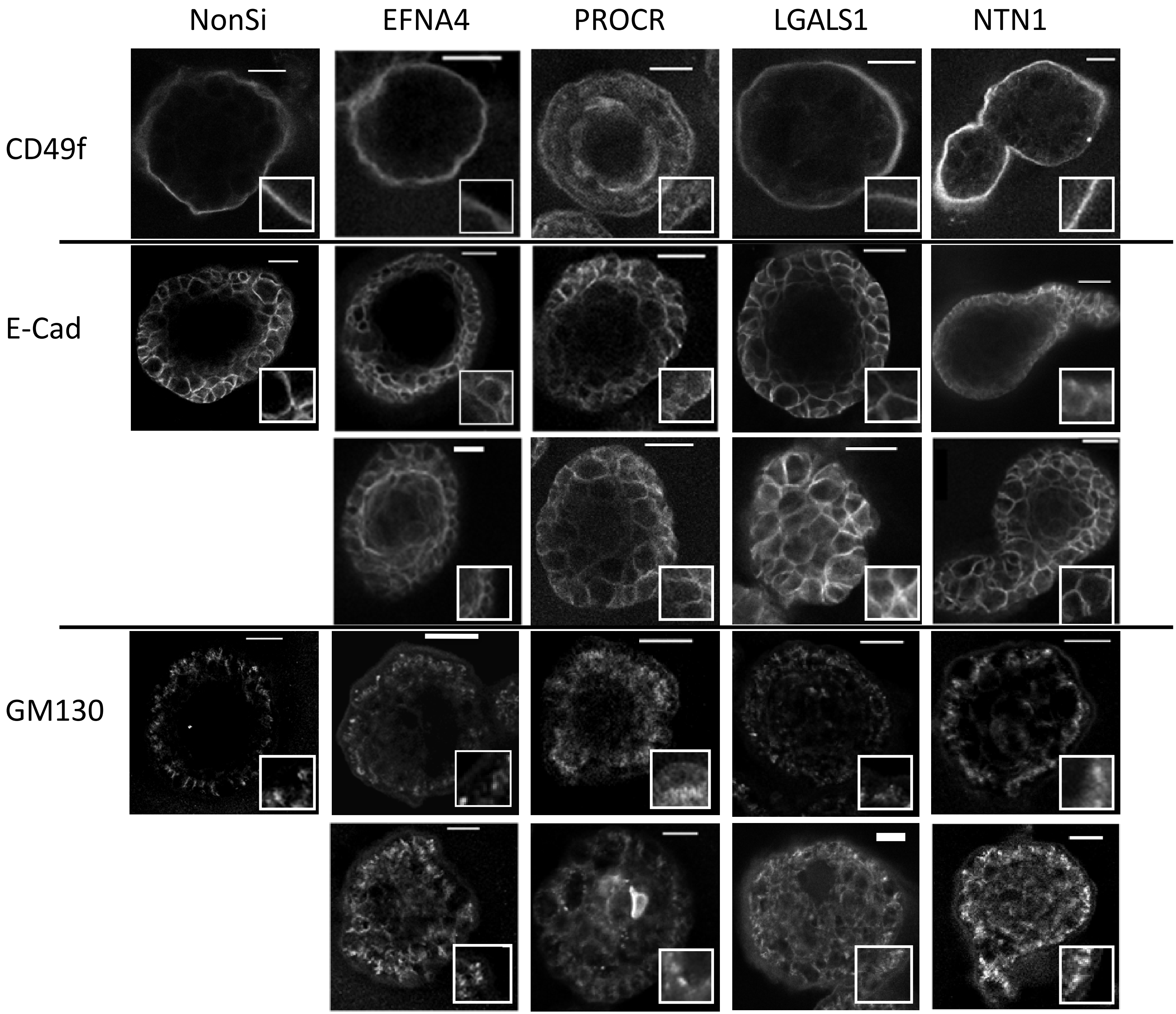

Supplement: Additional file 2: Figure S8. — Epithelial organization is disrupted in three-dimensional culture with silencing of target genes. (A) 184-hTERT-L9 cell lines with stable integration of pGIPZ shRNA lentiviral constructs against EFNA4, LGALS1, NTN1, PROCR or a nontargeting control were seeded into three-dimensional Matrigel culture and fixed after 21 days of growth. Staining was performed with Alexa Fluor 546–conjugated phalloidin and DRAQ5 nuclear stain prior to imaging on a Nikon confocal laser scanning microscope. Magnified views of representative structures for each condition are presented for CD49f, E-cadherin and GM130 staining. (B) The same images shown in (A) are depicted with greyscale inverted for visual clarity. [file 13058_2014_510_MOESM2_ESM.zip › 3501832231099773_add8.pdf]
