# Supplementary material for: A co-culture genome-wide RNAi screen with mammary epithelial cells reveals transmembrane signals required for growth and differentiation
Source: Breast Cancer Res. 2015 Jan 9;17:4. doi: 10.1186/s13058-014-0510-y (PMC4322558; doi:10.1186/s13058-014-0510-y)
Supplement: Additional file 12: Figure S9. — Beanplots depicting target gene expression in the ten biological primary breast cancer subgroups. The individual observations are shown as small horizontal lines in a one-dimensional scatterplot with the estimated density of the distributions shown in colour and the average indicated by the long horizontal line. [file 13058_2014_510_MOESM12_ESM.pdf]

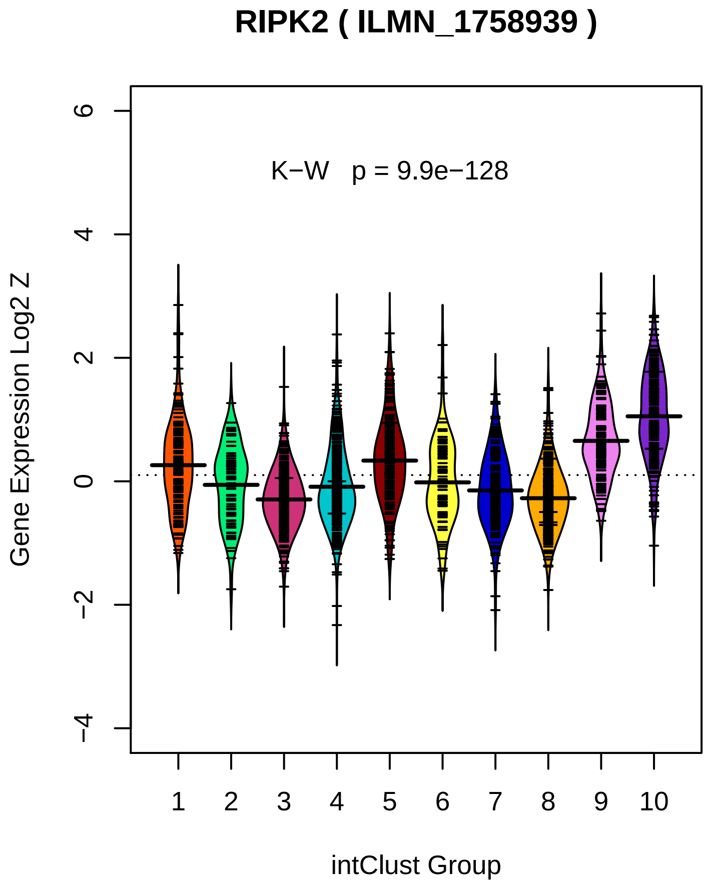
RIPK2 (ILMN_1758939)
K−W p = 9.9e−128
Gene Expression Log2 Z
intClust Group
1 2 3 4 5 6 7 8 9 10
-4 -2 0 2 4 6

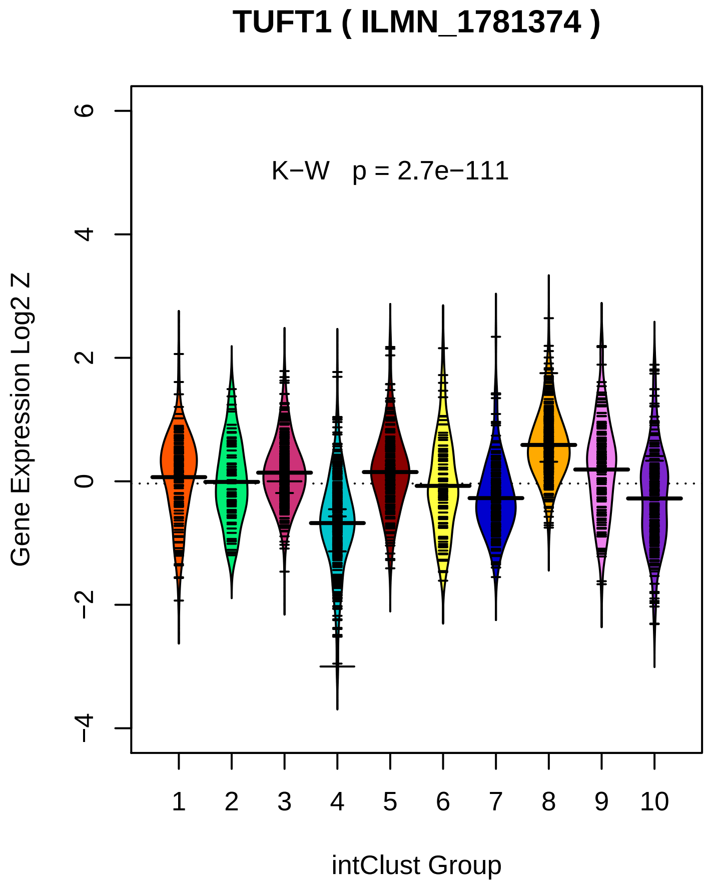
TUFT1 (ILMN_1781374)
K−W p = 2.7e−111
Gene Expression Log2 Z
intClust Group
1 2 3 4 5 6 7 8 9 10
-4 -2 0 2 4 6

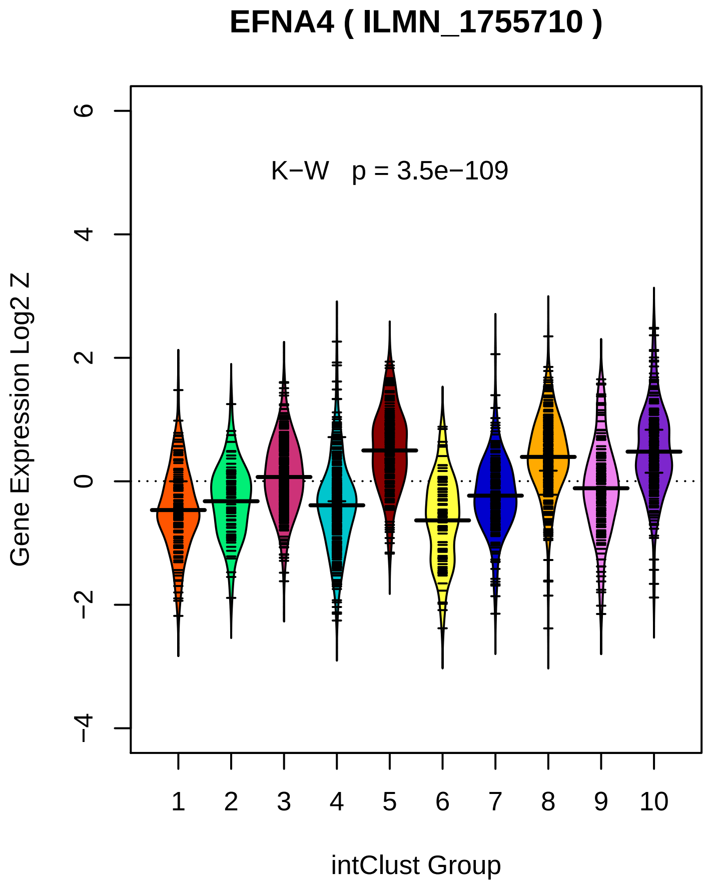
EFNA4 (ILMN_1755710)
K−W p = 3.5e−109
Gene Expression Log2 Z
intClust Group
1 2 3 4 5 6 7 8 9 10
-4 -2 0 2 4 6

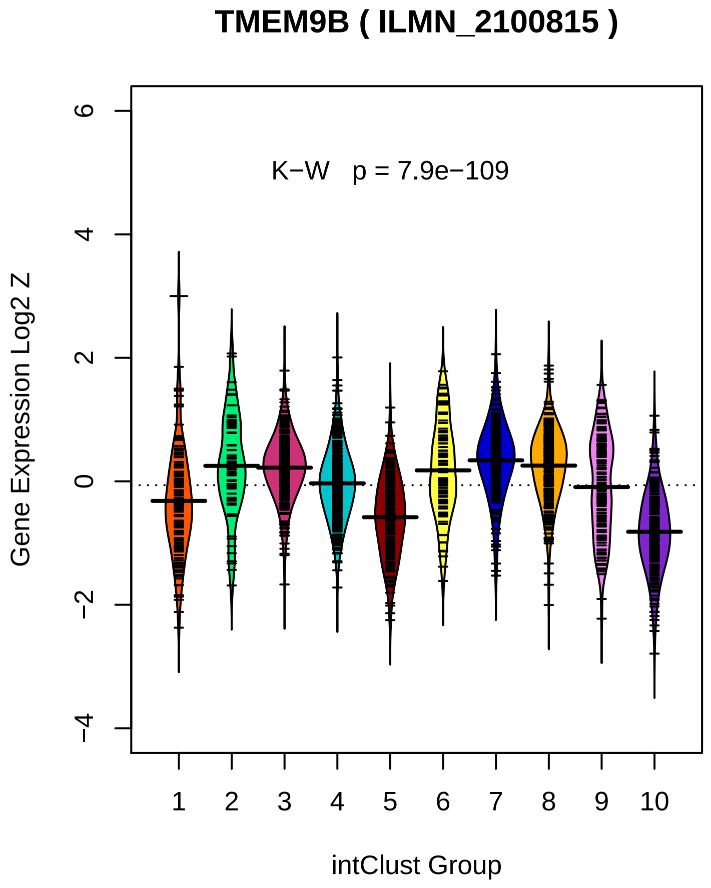
TMEM9B (ILMN_2100815)
K−W p = 7.9e−109
Gene Expression Log2 Z
intClust Group
1 2 3 4 5 6 7 8 9 10
-4 -2 0 2 4 6

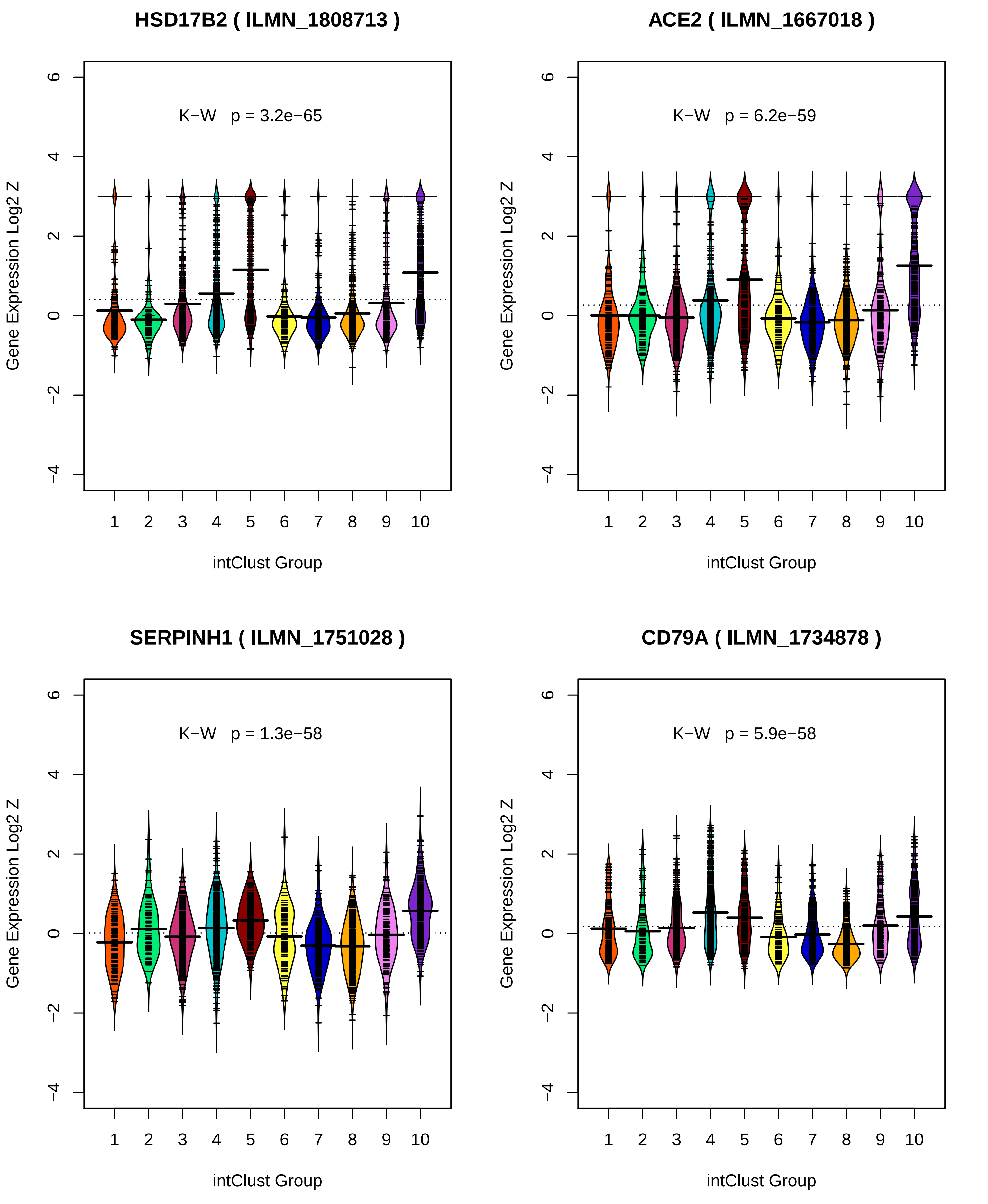

HSD17B2 (ILMN_1808713)
K−W p = 3.2e−65
ACE2 (ILMN_1667018)
K−W p = 6.2e−59
SERPINH1 (ILMN_1751028)
K−W p = 1.3e−58
CD79A (ILMN_1734878)
K−W p = 5.9e−58
Gene Expression Log2 Z
intClust Group
1 2 3 4 5 6 7 8 9 10
-4 -2 0 2 4 6

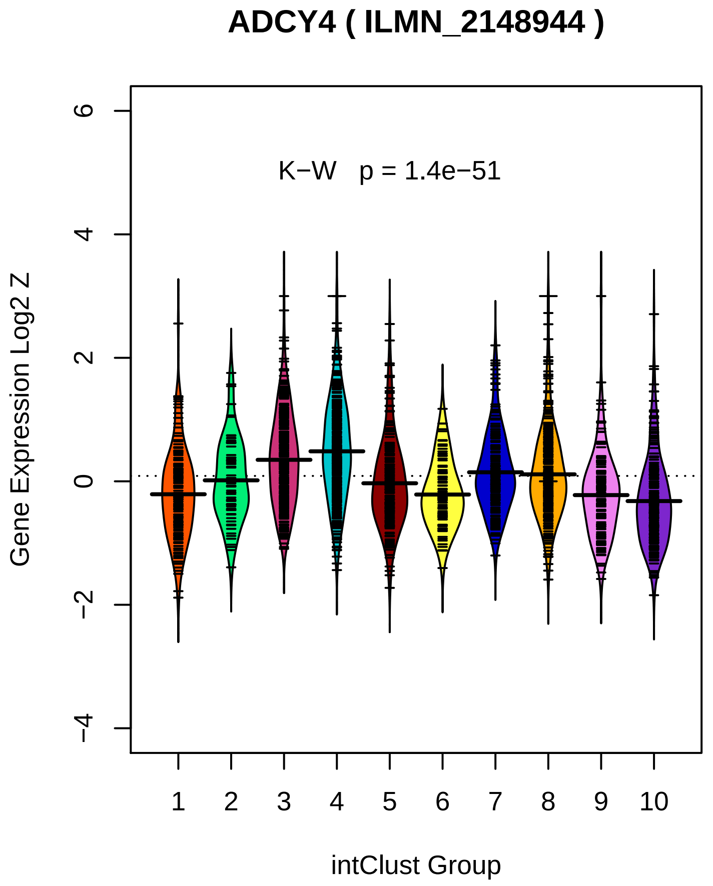
ADCY4 (ILMN_2148944)
K−W p = 1.4e−51
Gene Expression Log2 Z
-4
-2
0
2
4
6
1 2 3 4 5 6 7 8 9 10
intClust Group

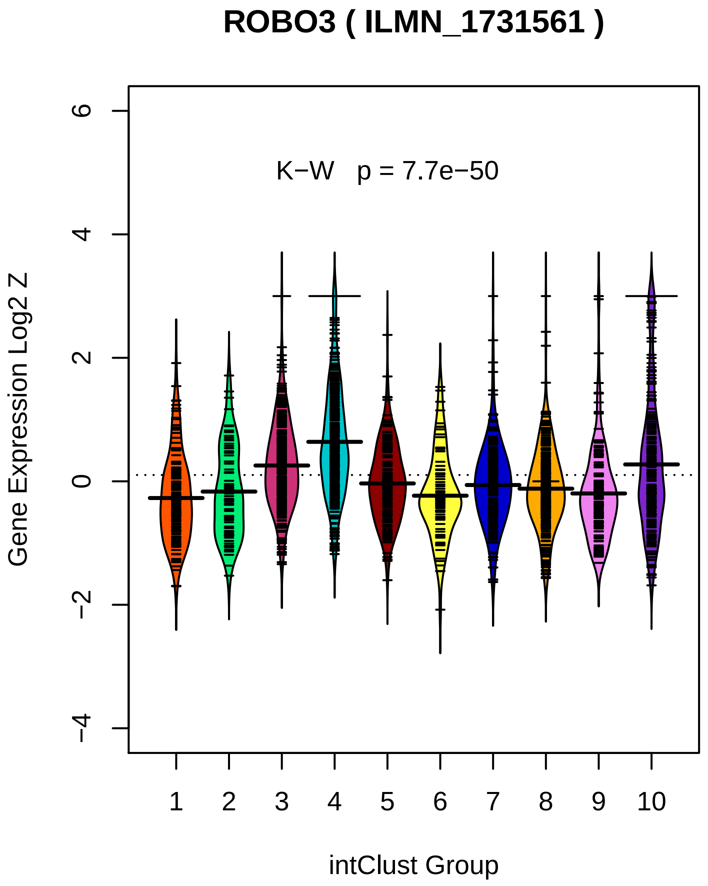
ROBO3 (ILMN_1731561)
K−W p = 7.7e−50
Gene Expression Log2 Z
-4
-2
0
2
4
6
1 2 3 4 5 6 7 8 9 10
intClust Group

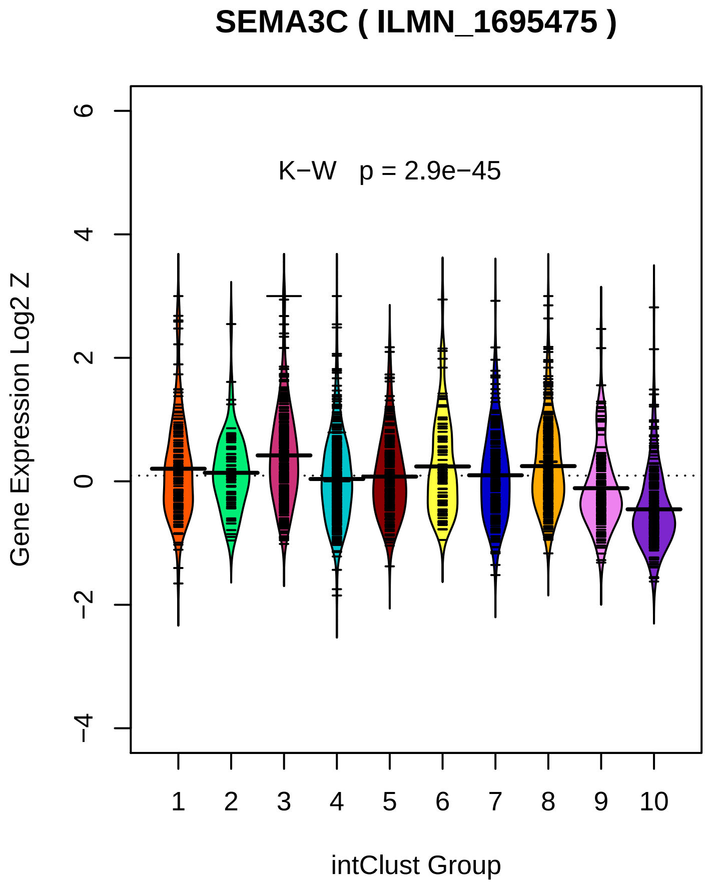
SEMA3C (ILMN_1695475)
K−W p = 2.9e−45
Gene Expression Log2 Z
-4
-2
0
2
4
6
1 2 3 4 5 6 7 8 9 10
intClust Group

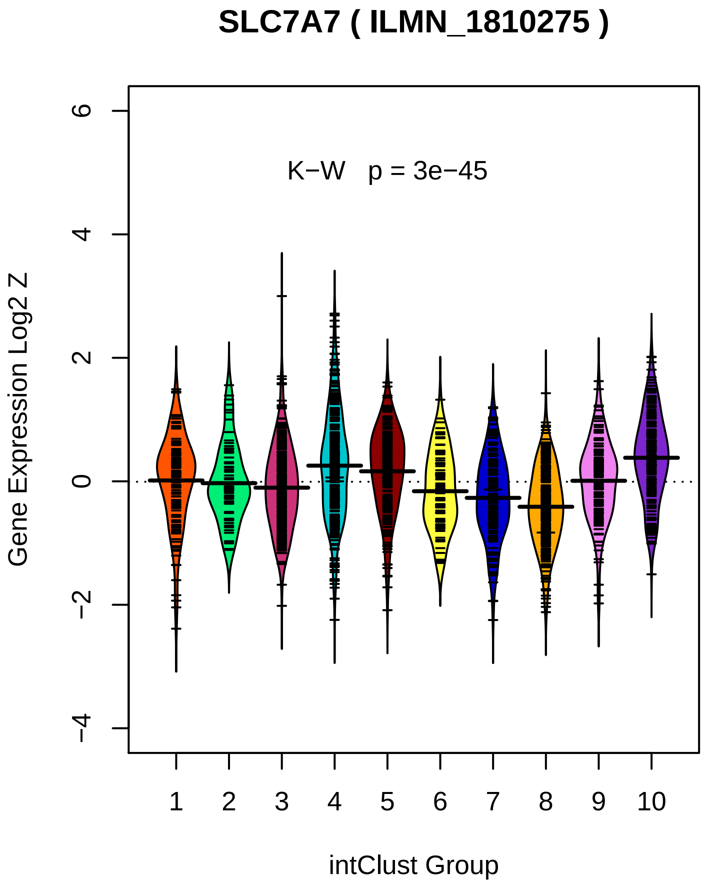
SLC7A7 (ILMN_1810275)
K−W p = 3e−45
Gene Expression Log2 Z
-4
-2
0
2
4
6
1 2 3 4 5 6 7 8 9 10
intClust Group

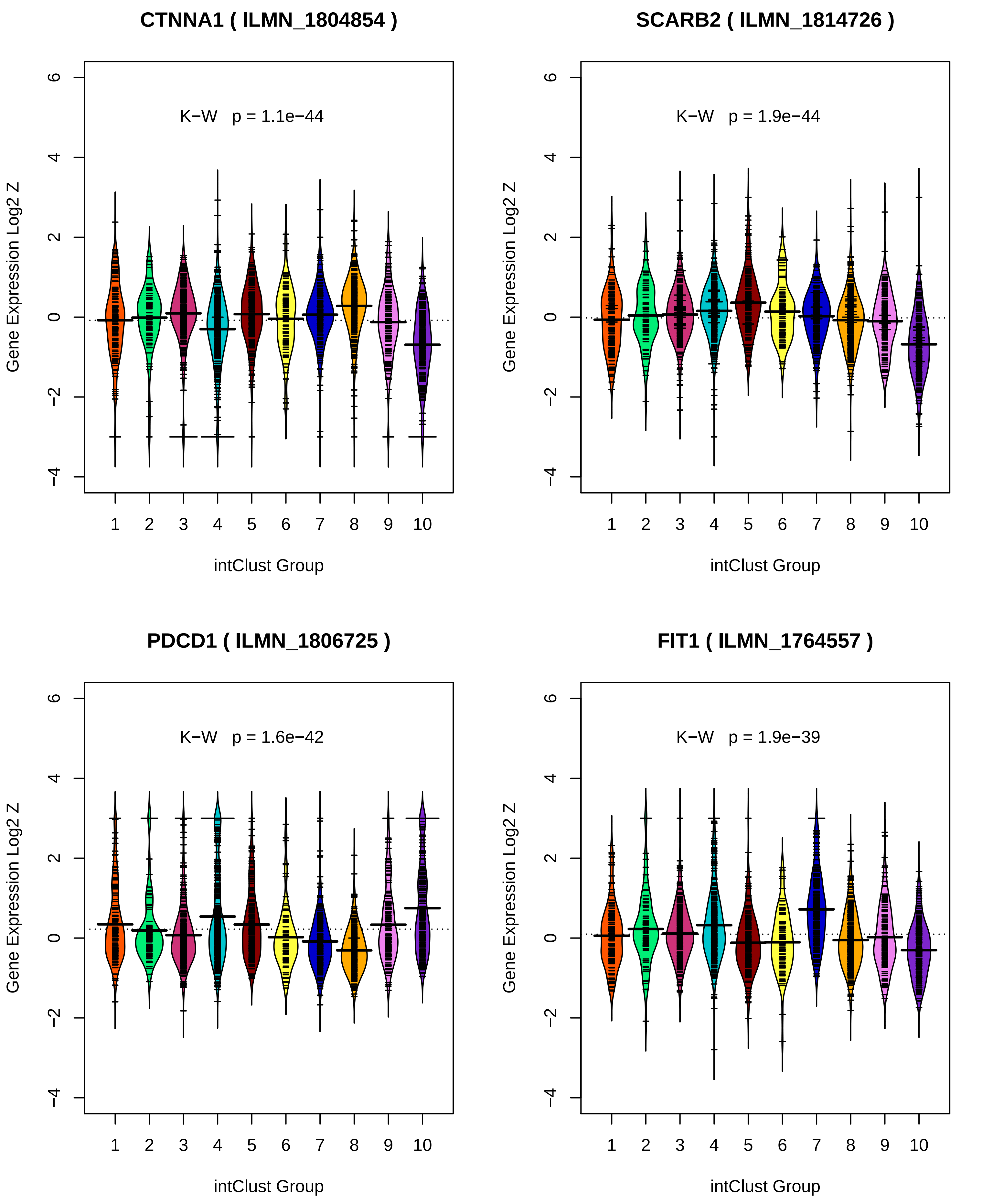

CTNNA1 (ILMN_1804854)
K−W p = 1.1e−44
SCARB2 (ILMN_1814726)
K−W p = 1.9e−44
PDCD1 (ILMN_1806725)
K−W p = 1.6e−42
FIT1 (ILMN_1764557)
K−W p = 1.9e−39
Gene Expression Log2 Z
intClust Group
1
2
3
4
5
6
7
8
9
10
6
4
2
0
−2
−4

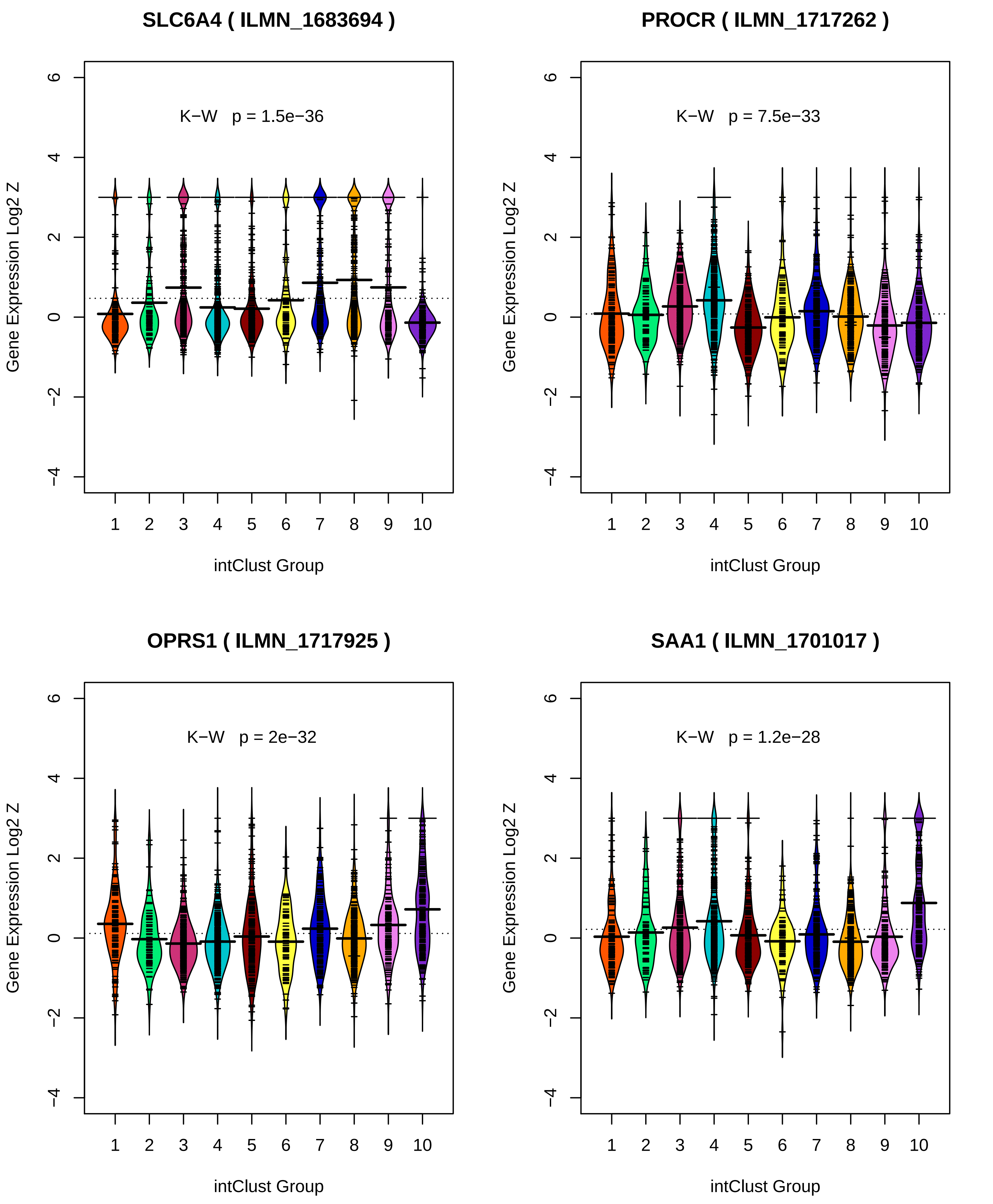

SLC6A4 (ILMN_1683694)
K−W p = 1.5e−36
PROCR (ILMN_1717262)
K−W p = 7.5e−33
OPRS1 (ILMN_1717925)
K−W p = 2e−32
SAA1 (ILMN_1701017)
K−W p = 1.2e−28
Gene Expression Log2 Z
intClust Group

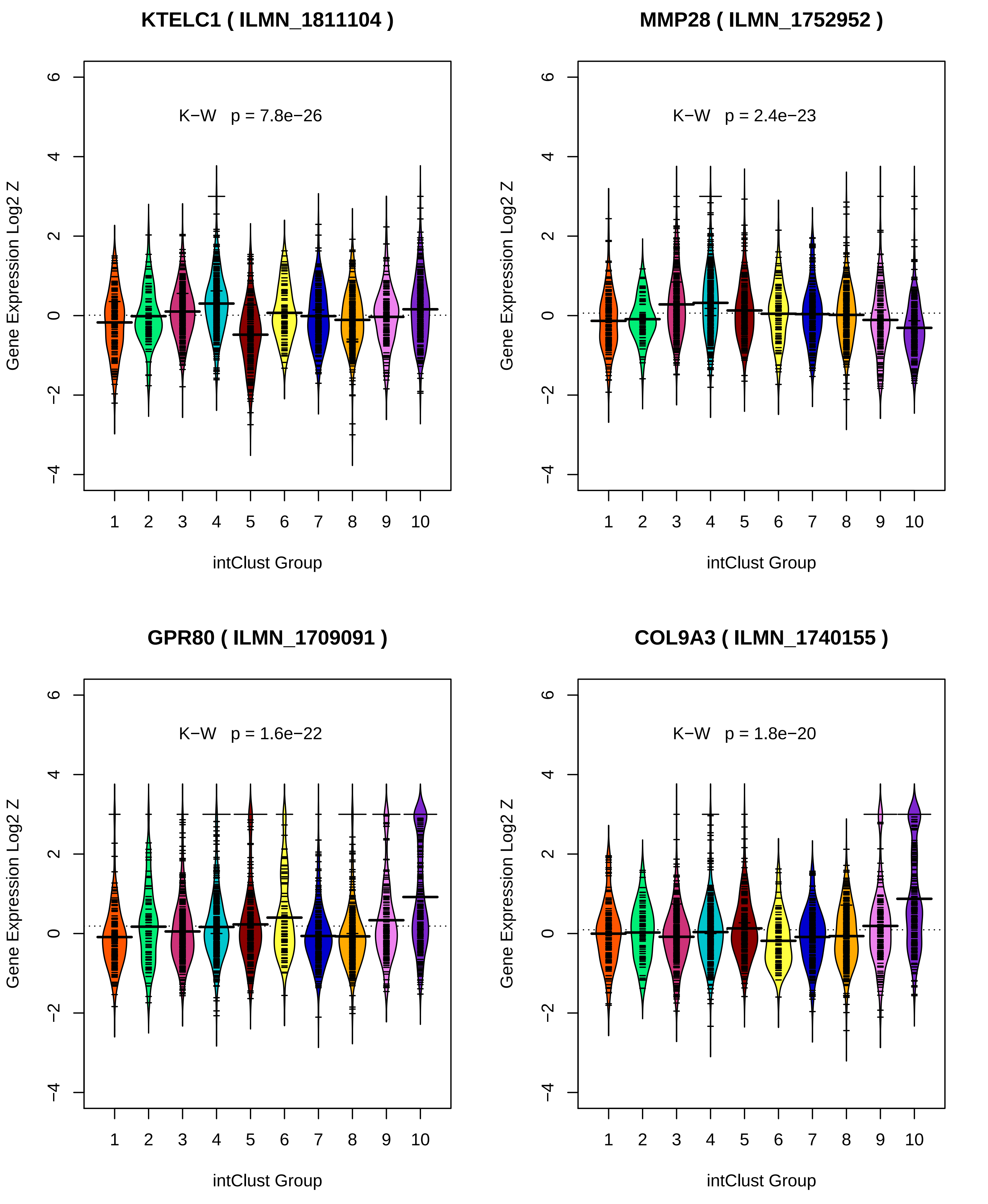
KTELC1 (ILMN_1811104)
K−W p = 7.8e−26
MMP28 (ILMN_1752952)
K−W p = 2.4e−23
GPR80 (ILMN_1709091)
K−W p = 1.6e−22
COL9A3 (ILMN_1740155)
K−W p = 1.8e−20
Gene Expression Log2 Z
intClust Group
1 2 3 4 5 6 7 8 9 10
−4 −2 0 2 4 6

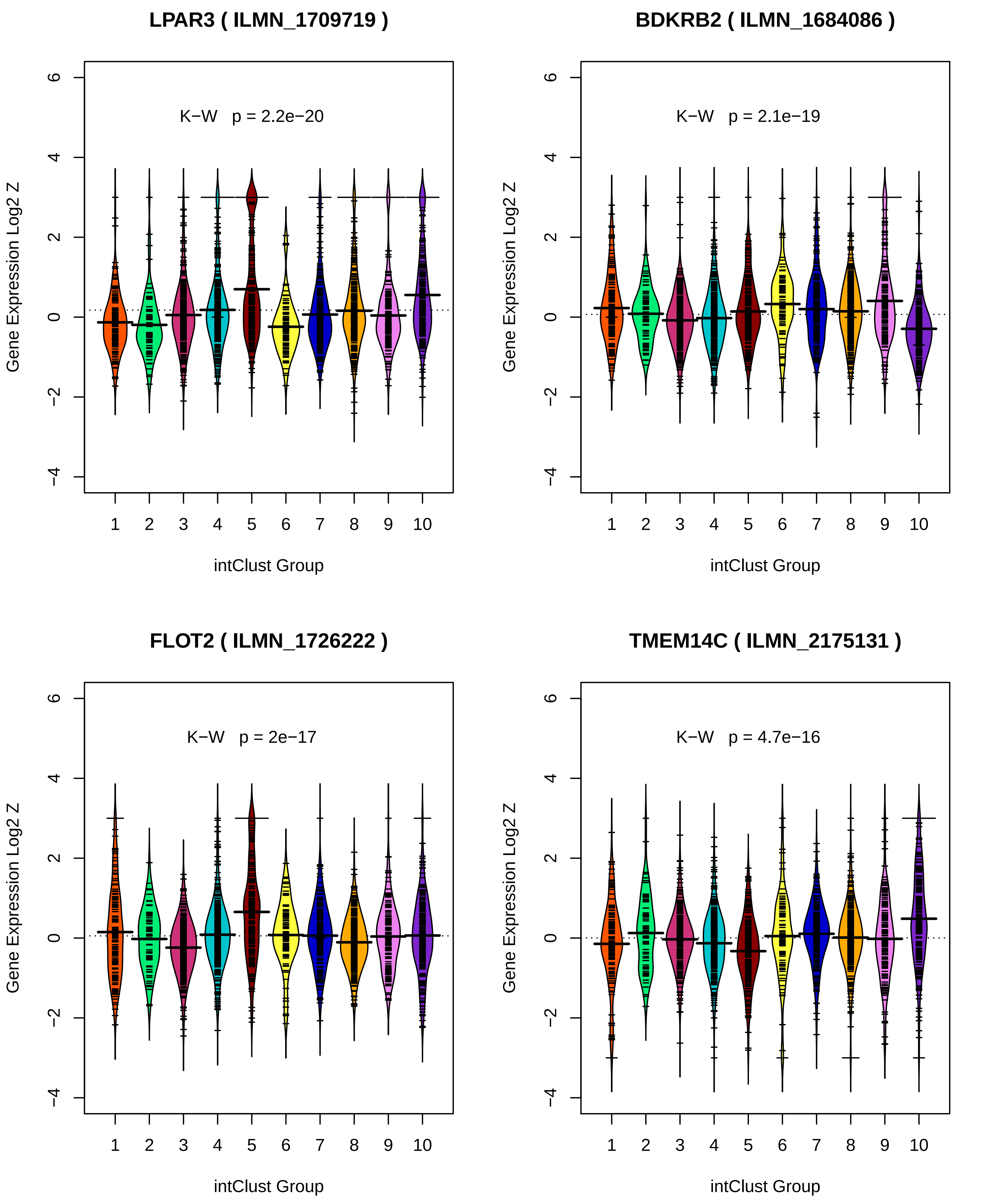

LPAR3 (ILMN_1709719)
K−W p = 2.2e−20
BDKRB2 (ILMN_1684086)
K−W p = 2.1e−19
FLOT2 (ILMN_1726222)
K−W p = 2e−17
TMEM14C (ILMN_2175131)
K−W p = 4.7e−16
Gene Expression Log2 Z
intClust Group

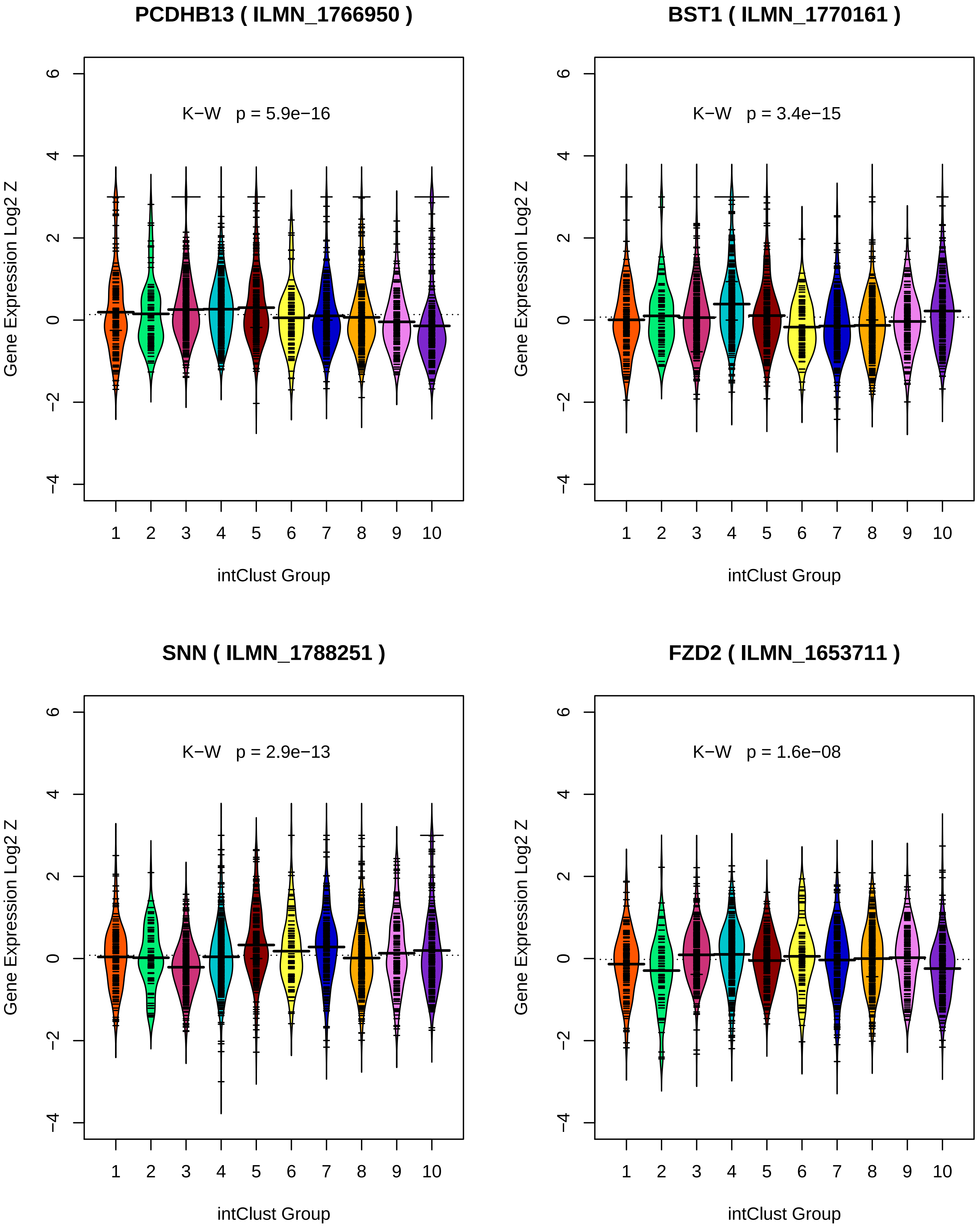
PCDHB13 (ILMN_1766950)
K−W p = 5.9e−16
BST1 (ILMN_1770161)
K−W p = 3.4e−15
SNN (ILMN_1788251)
K−W p = 2.9e−13
FZD2 (ILMN_1653711)
K−W p = 1.6e−08
Gene Expression Log2 Z
intClust Group
−4
−2
0
2
4
6
1
2
3
4
5
6
7
8
9
10

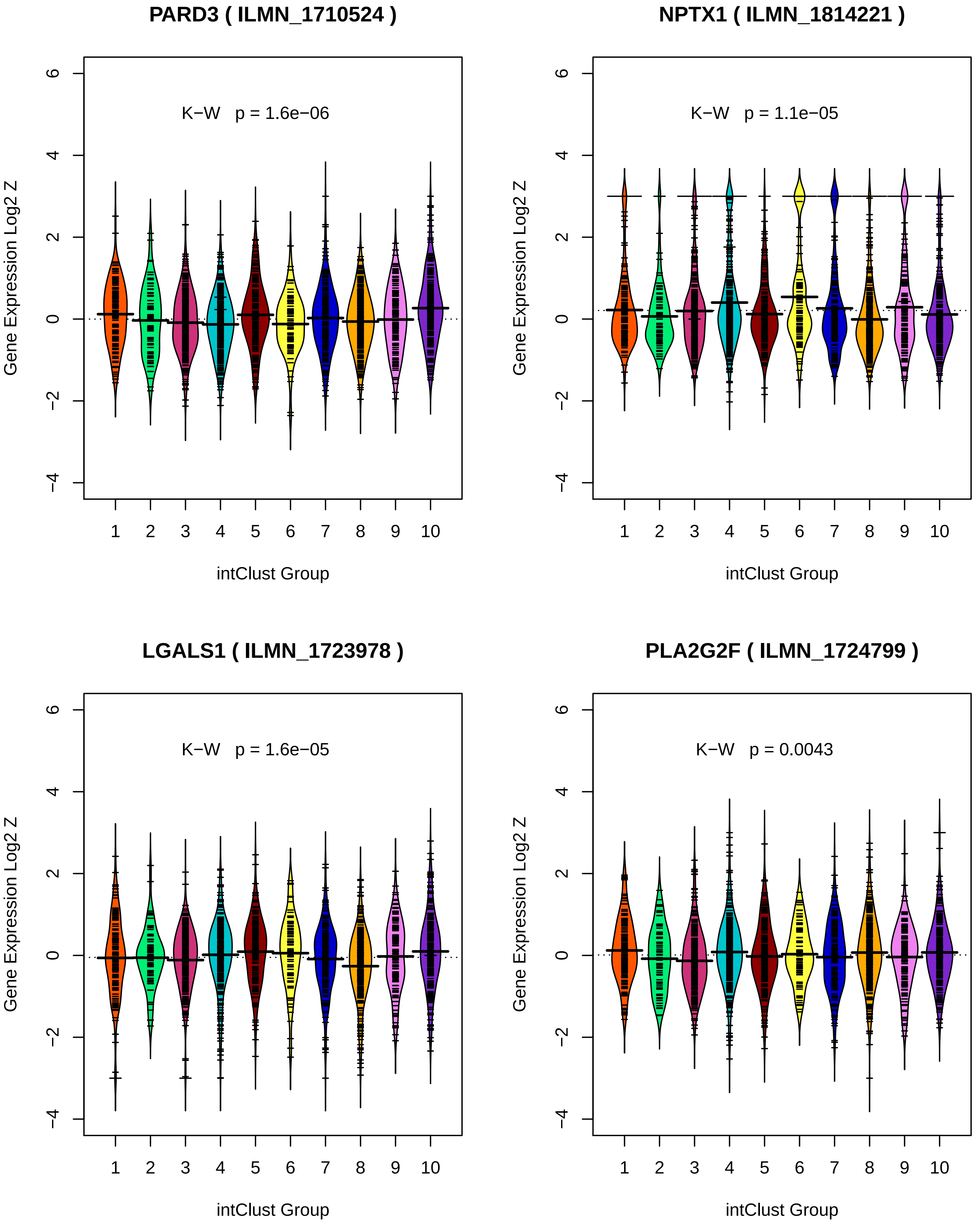
PARD3 (ILMN_1710524)
K−W p = 1.6e−06
Gene Expression Log2 Z
intClust Group
NPTX1 (ILMN_1814221)
K−W p = 1.1e−05
Gene Expression Log2 Z
intClust Group
LGALS1 (ILMN_1723978)
K−W p = 1.6e−05
Gene Expression Log2 Z
intClust Group
PLA2G2F (ILMN_1724799)
K−W p = 0.0043
Gene Expression Log2 Z
intClust Group

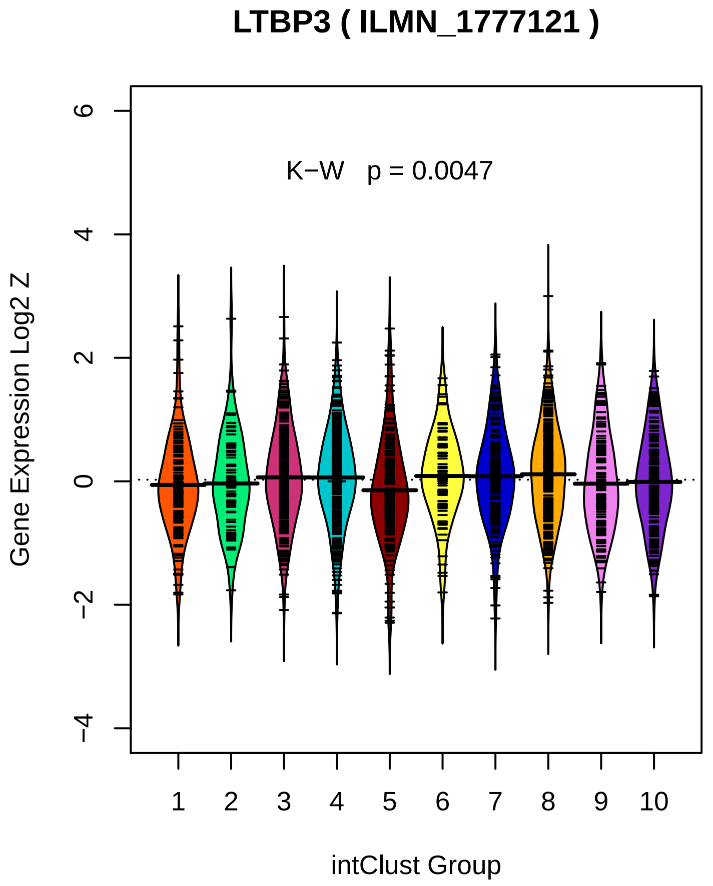
LTBP3 (ILMN_1777121)
K−W p = 0.0047
Gene Expression Log2 Z
intClust Group

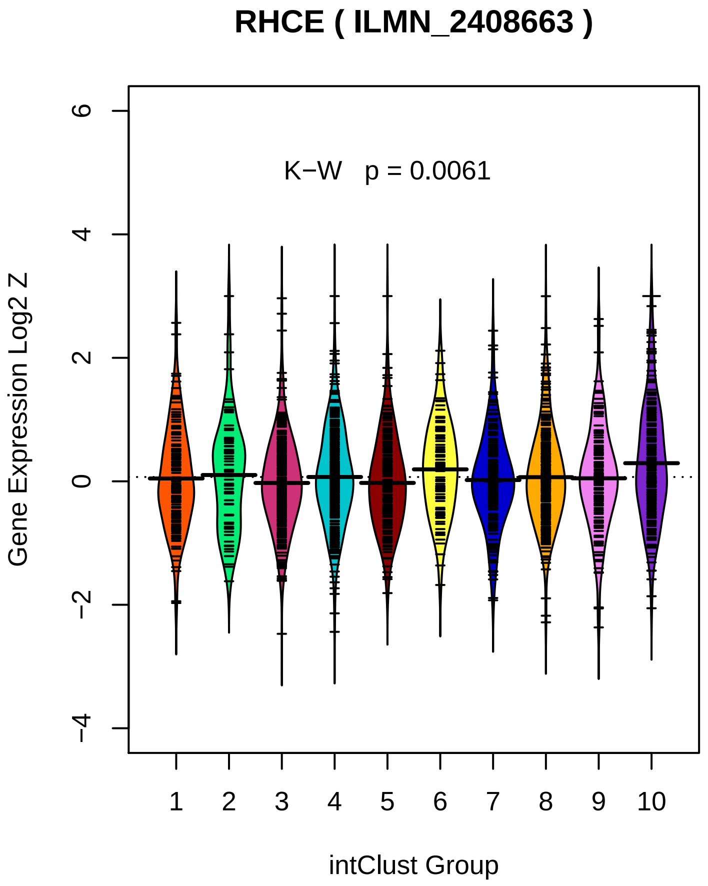
RHCE (ILMN_2408663)
K−W p = 0.0061
Gene Expression Log2 Z
intClust Group

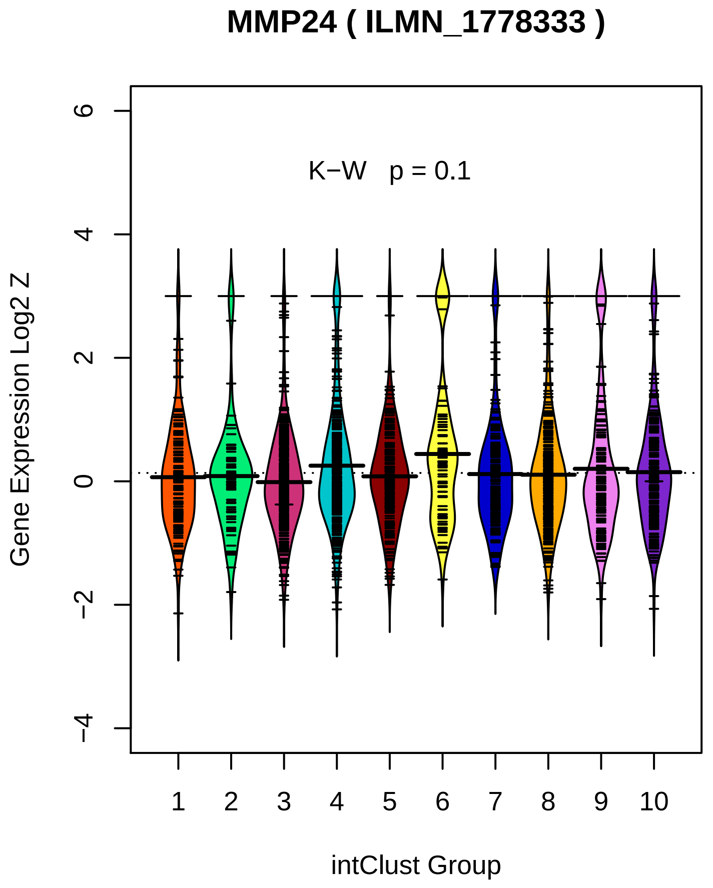
MMP24 (ILMN_1778333)
K−W p = 0.1
Gene Expression Log2 Z
intClust Group

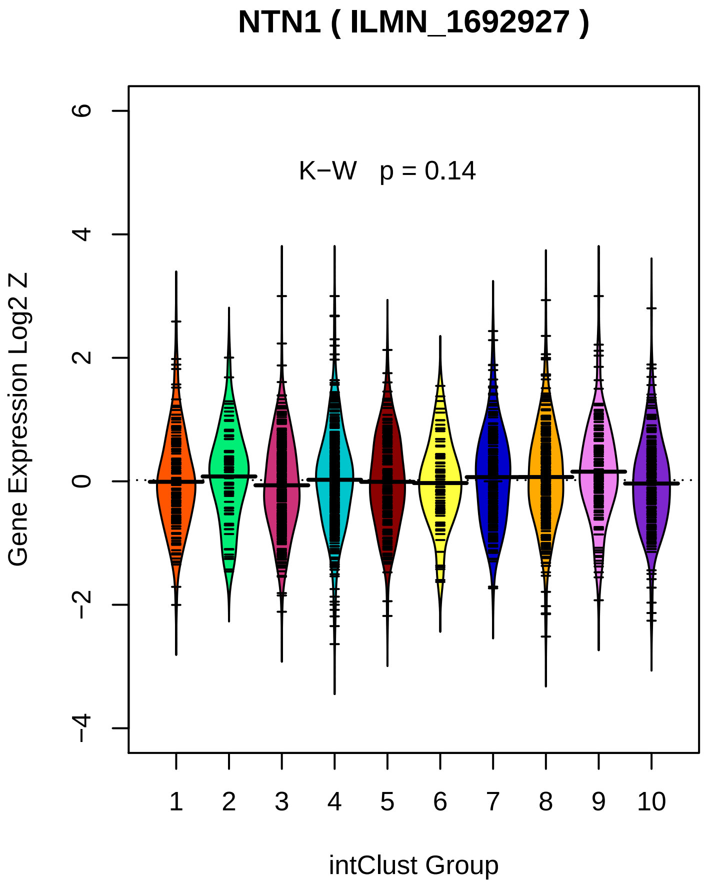
NTN1 (ILMN_1692927)
K−W p = 0.14
Gene Expression Log2 Z
intClust Group

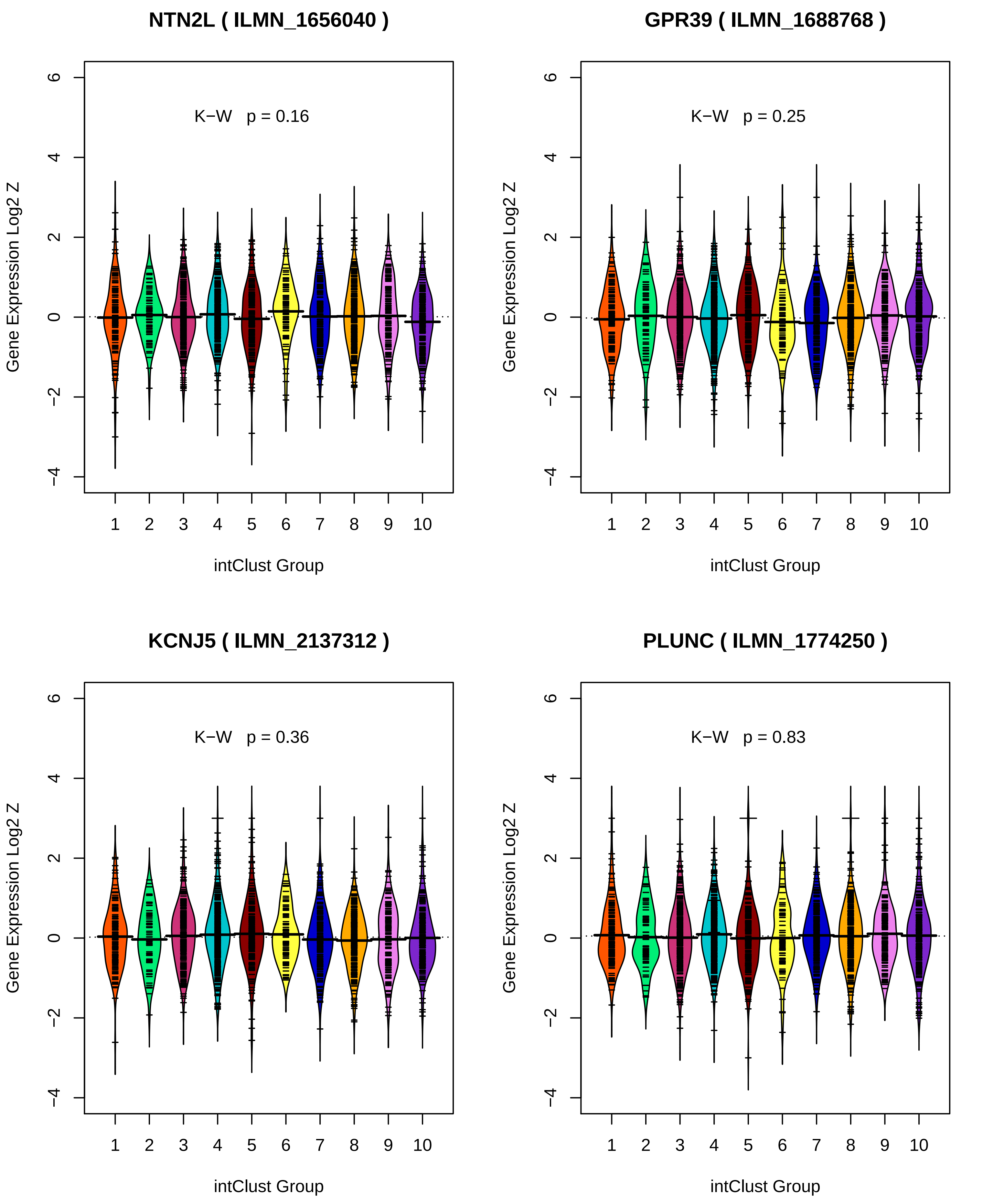
NTN2L (ILMN_1656040)
K−W p = 0.16
GPR39 (ILMN_1688768)
K−W p = 0.25
KCNJ5 (ILMN_2137312)
K−W p = 0.36
PLUNC (ILMN_1774250)
K−W p = 0.83
Gene Expression Log2 Z
intClust Group
1 2 3 4 5 6 7 8 9 10
−4 −2 0 2 4 6

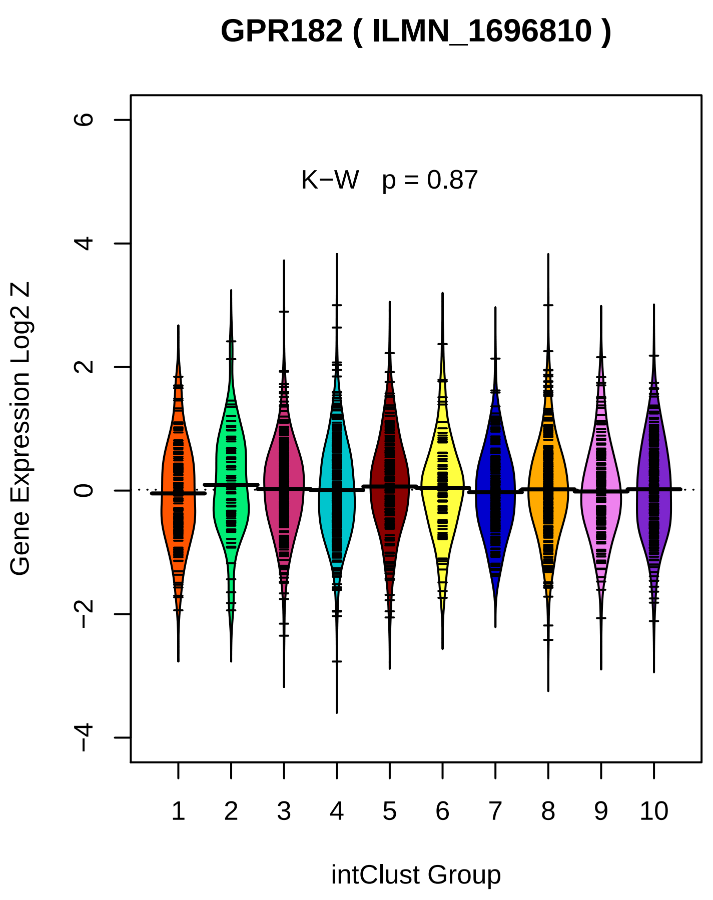
GPR182 (ILMN_1696810)
K−W p = 0.87
Gene Expression Log2 Z
6
4
2
0
−2
−4
1 2 3 4 5 6 7 8 9 10
intClust Group

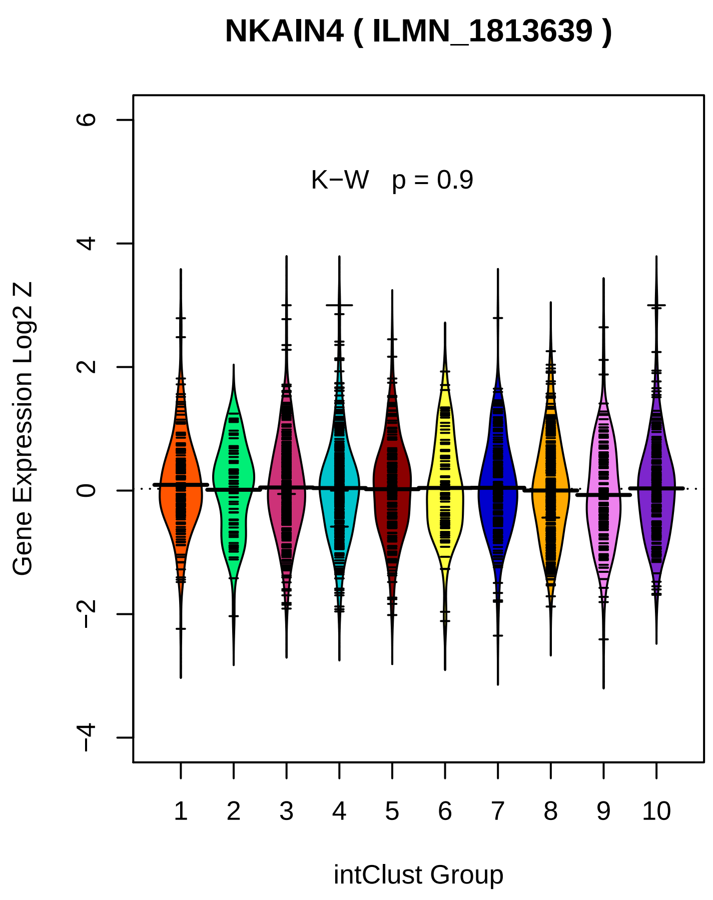
NKAIN4 (ILMN_1813639)
K−W p = 0.9
Gene Expression Log2 Z
6
4
2
0
−2
−4
1 2 3 4 5 6 7 8 9 10
intClust Group
