# Supplementary material for: A co-culture genome-wide RNAi screen with mammary epithelial cells reveals transmembrane signals required for growth and differentiation
Source: Breast Cancer Res. 2015 Jan 9;17:4. doi: 10.1186/s13058-014-0510-y (PMC4322558; doi:10.1186/s13058-014-0510-y)
Supplement: Additional file 13: Figure S10. — Beanplots depicting target gene expression in the PAM50 breast cancer subgroups. The individual observations are shown as small horizontal lines in a one-dimensional scatterplot with the estimated density of the distributions shown in colour and the average indicated by the long horizontal line. [file 13058_2014_510_MOESM13_ESM.pdf]

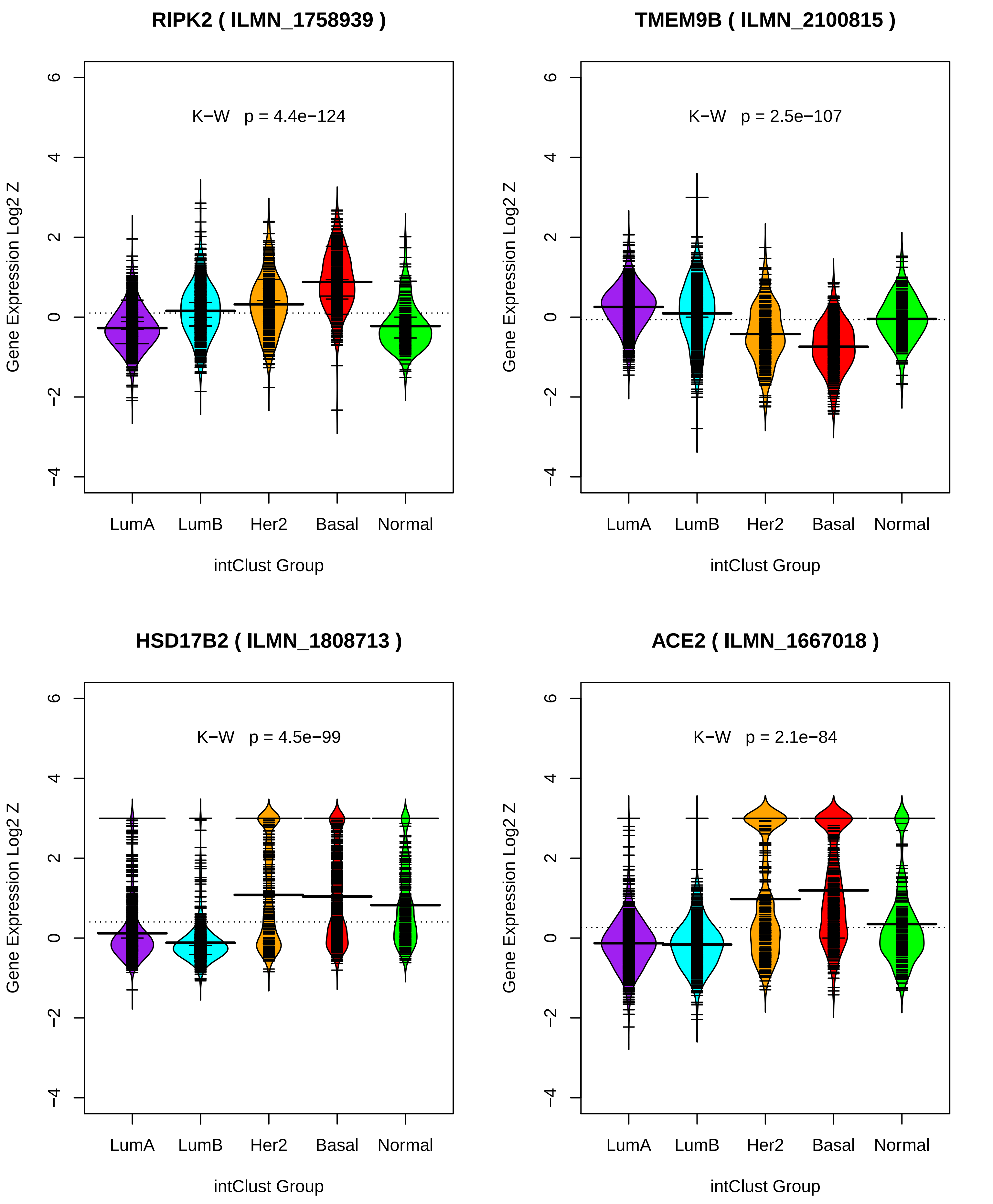
RIPK2 (ILMN_1758939)
K−W p = 4.4e−124
TMEM9B (ILMN_2100815)
K−W p = 2.5e−107
HSD17B2 (ILMN_1808713)
K−W p = 4.5e−99
ACE2 (ILMN_1667018)
K−W p = 2.1e−84
Gene Expression Log2 Z
intClust Group
LumA
LumB
Her2
Basal
Normal
6
4
2
0
−2
−4

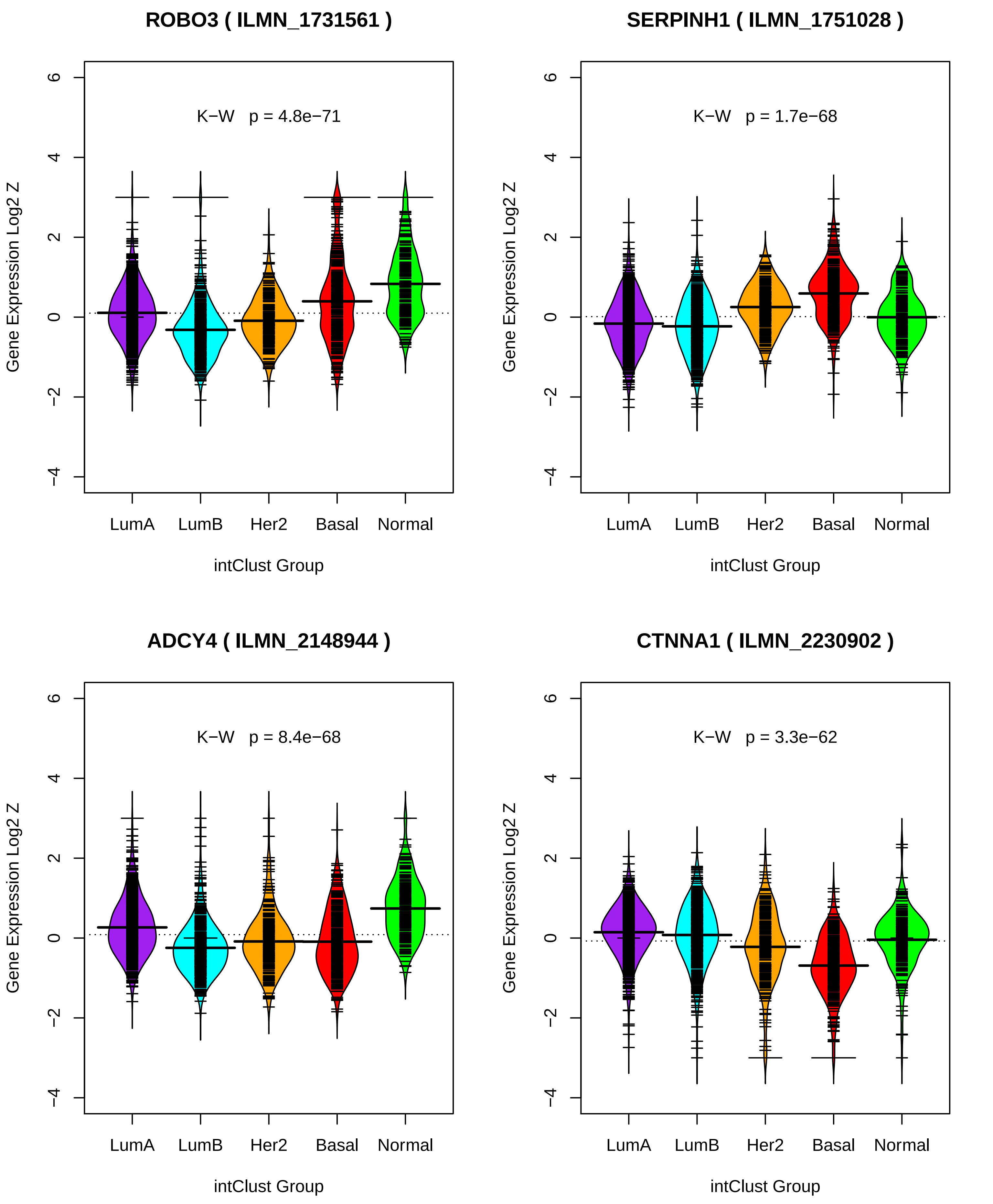

ROBO3 (ILMN_1731561)
K−W p = 4.8e−71
SERPINH1 (ILMN_1751028)
K−W p = 1.7e−68
ADCY4 (ILMN_2148944)
K−W p = 8.4e−68
CTNNA1 (ILMN_2230902)
K−W p = 3.3e−62
Gene Expression Log2 Z
6
4
2
0
−2
−4
LumA
LumB
Her2
Basal
Normal
intClust Group

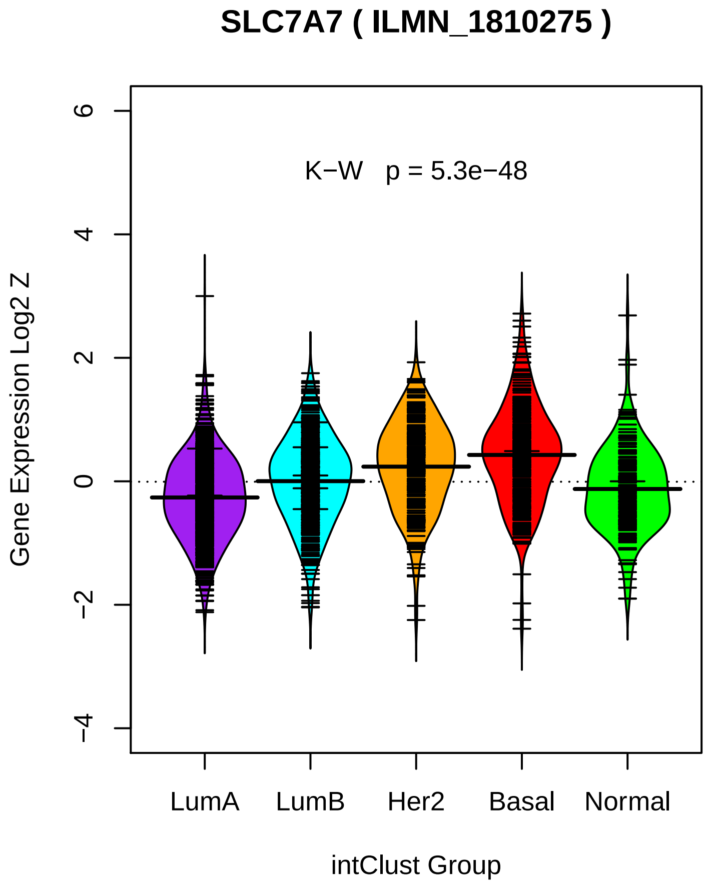
SLC7A7 (ILMN_1810275)
K−W p = 5.3e−48
Gene Expression Log2 Z
6
4
2
0
−2
−4
LumA
LumB
Her2
Basal
Normal
intClust Group

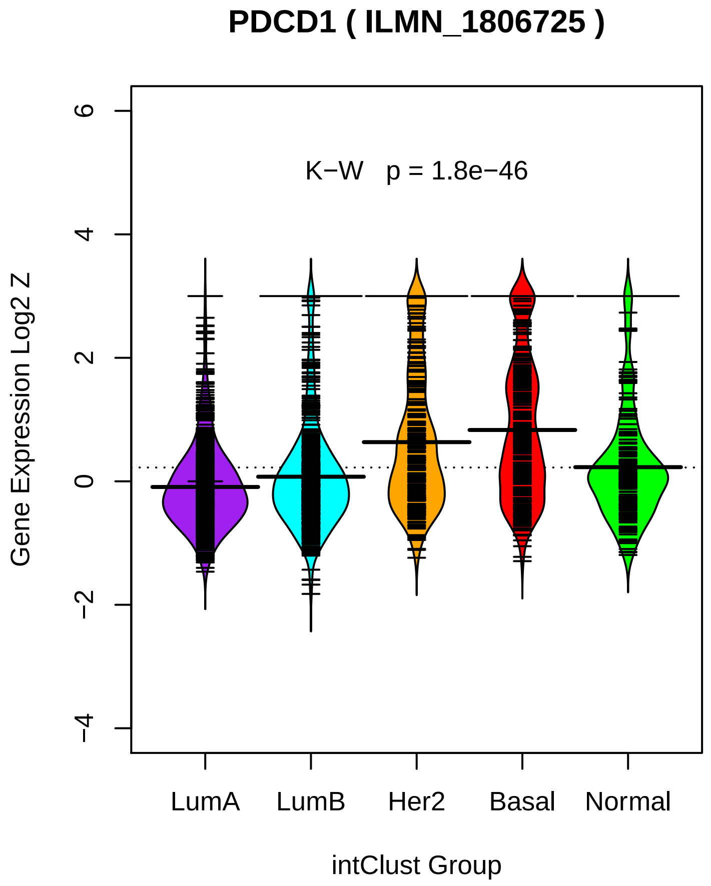
PDCD1 (ILMN_1806725)
K−W p = 1.8e−46
Gene Expression Log2 Z
6
4
2
0
−2
−4
LumA
LumB
Her2
Basal
Normal
intClust Group

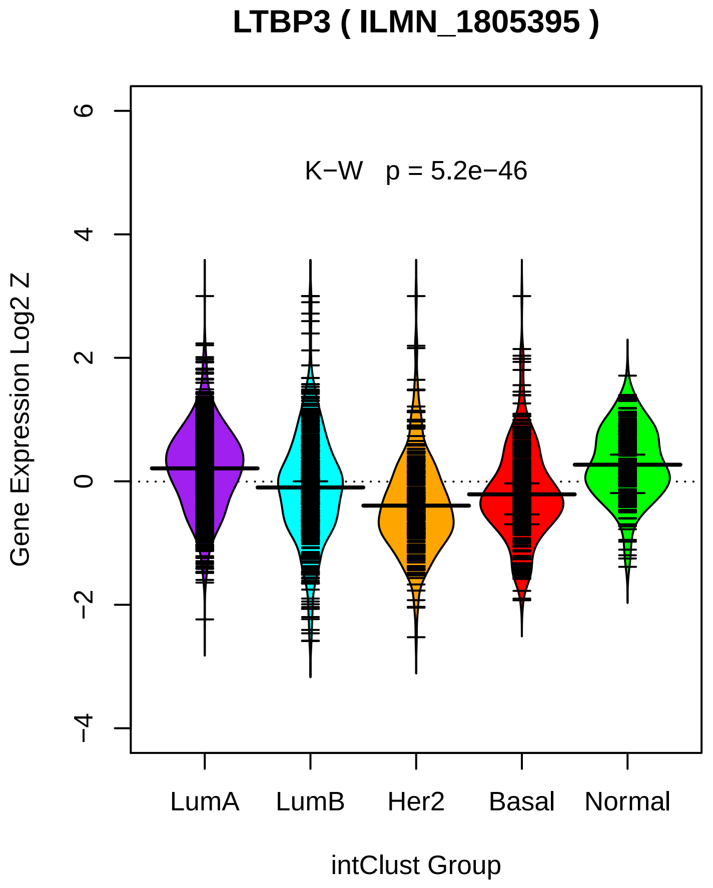
LTBP3 (ILMN_1805395)
K−W p = 5.2e−46
Gene Expression Log2 Z
6
4
2
0
−2
−4
LumA
LumB
Her2
Basal
Normal
intClust Group

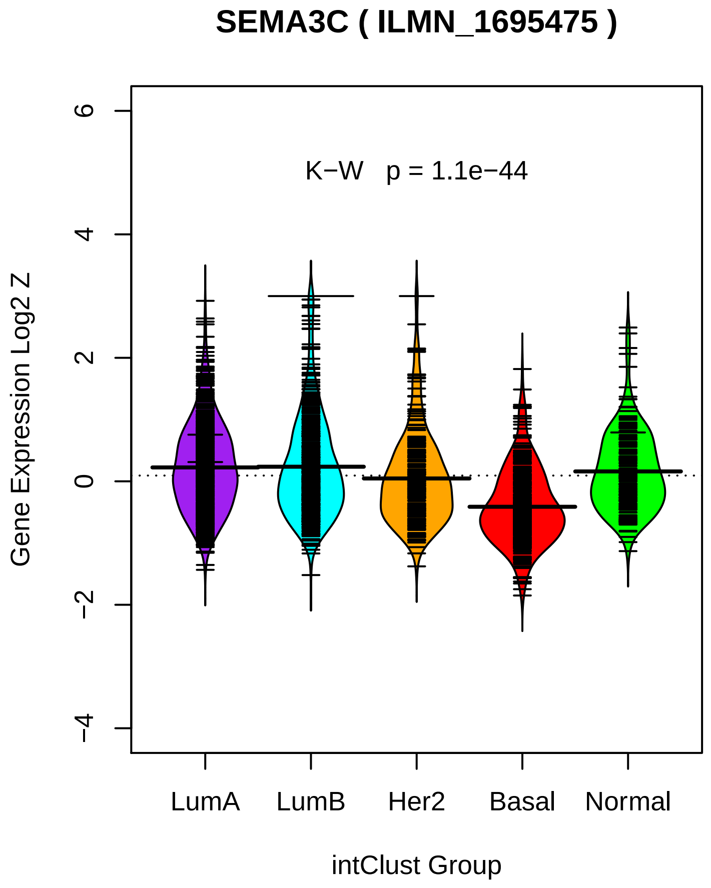
SEMA3C (ILMN_1695475)
K−W p = 1.1e−44
Gene Expression Log2 Z
6
4
2
0
−2
−4
LumA
LumB
Her2
Basal
Normal
intClust Group

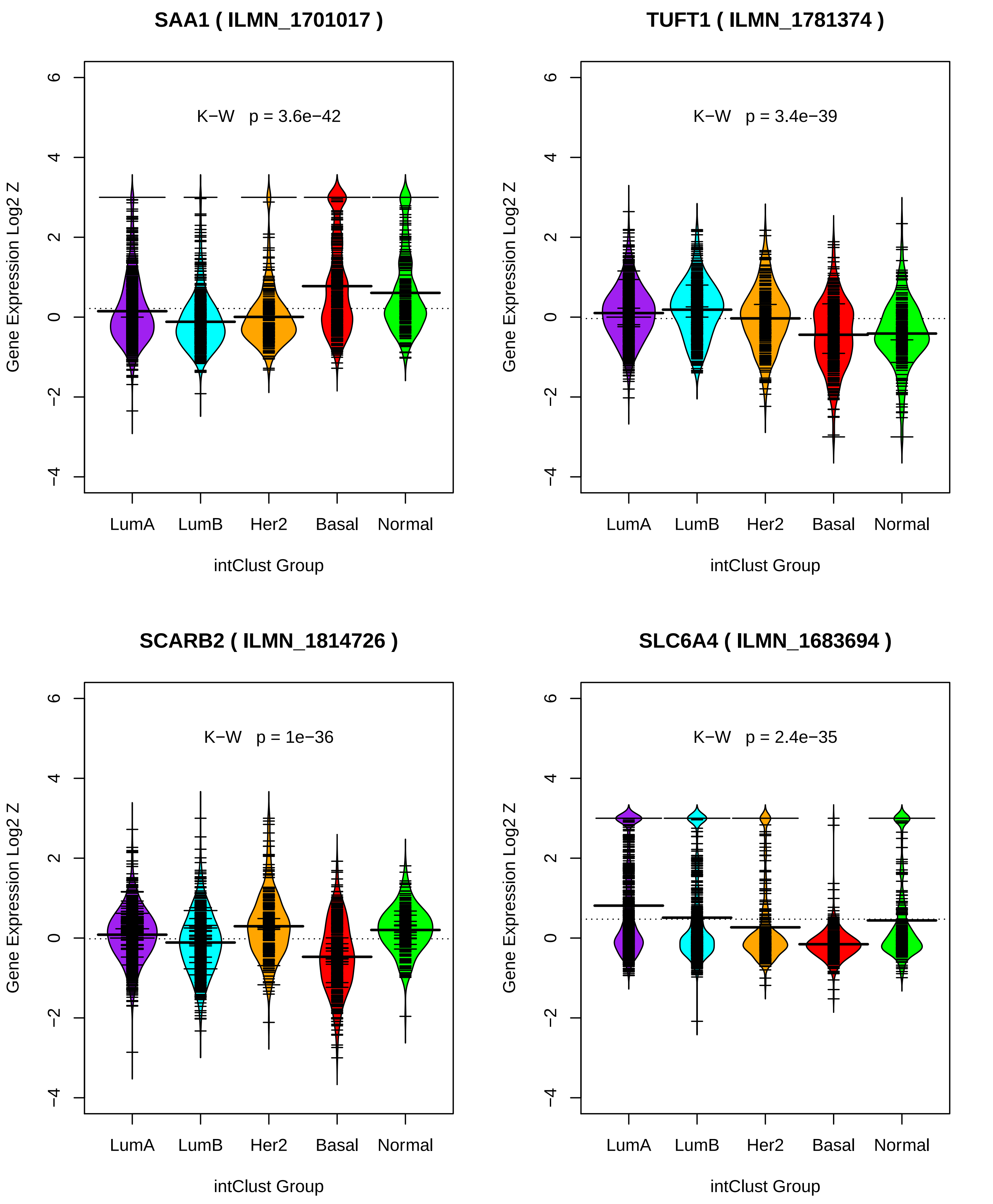
SAA1 (ILMN_1701017)
K−W p = 3.6e−42
TUFT1 (ILMN_1781374)
K−W p = 3.4e−39
SCARB2 (ILMN_1814726)
K−W p = 1e−36
SLC6A4 (ILMN_1683694)
K−W p = 2.4e−35
Gene Expression Log2 Z
6
4
2
0
−2
−4
LumA
LumB
Her2
Basal
Normal
intClust Group

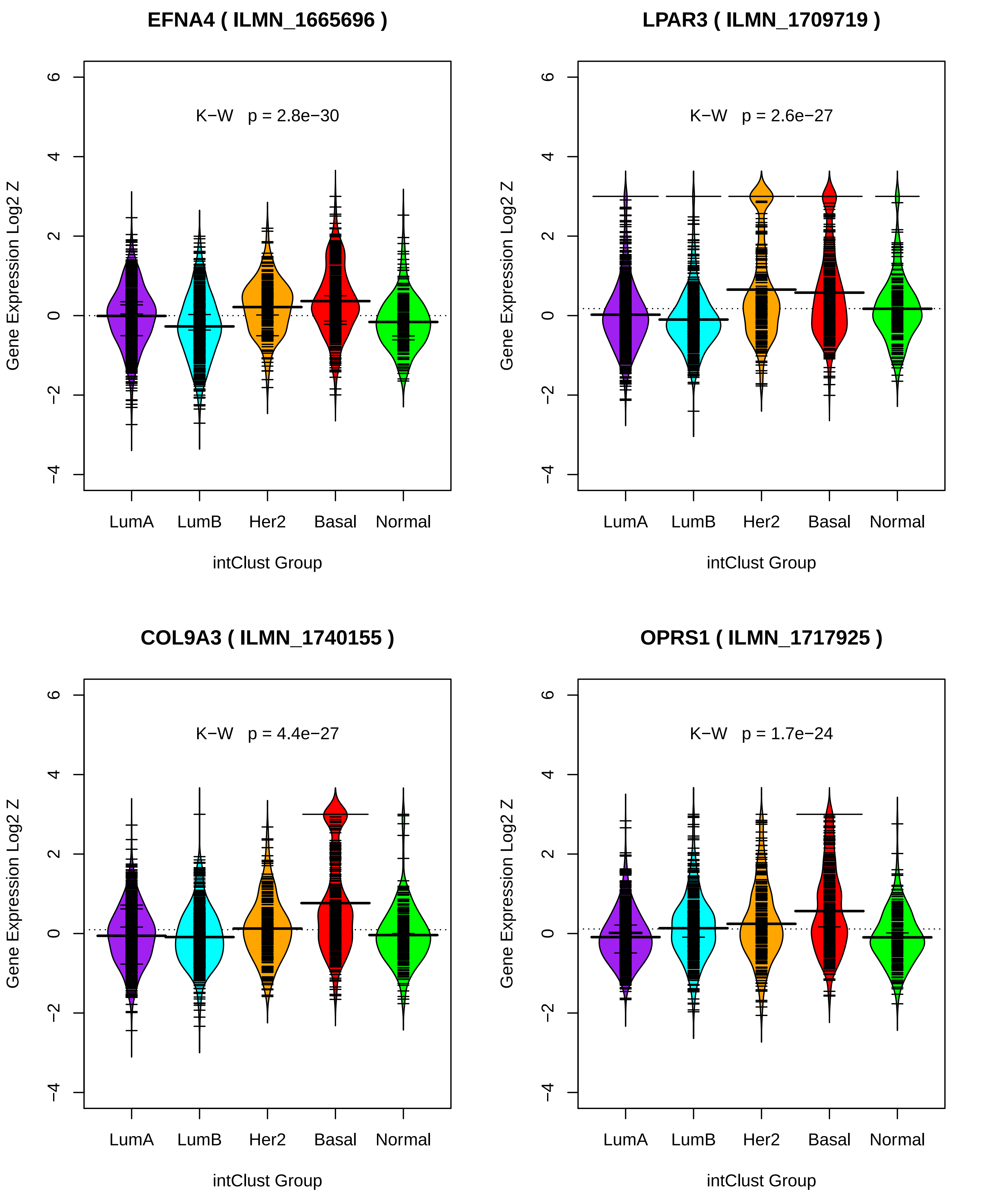
EFNA4 (ILMN_1665696)
K−W p = 2.8e−30
LPAR3 (ILMN_1709719)
K−W p = 2.6e−27
COL9A3 (ILMN_1740155)
K−W p = 4.4e−27
OPRS1 (ILMN_1717925)
K−W p = 1.7e−24
Gene Expression Log2 Z
-4
-2
0
2
4
6
LumA
LumB
Her2
Basal
Normal
intClust Group

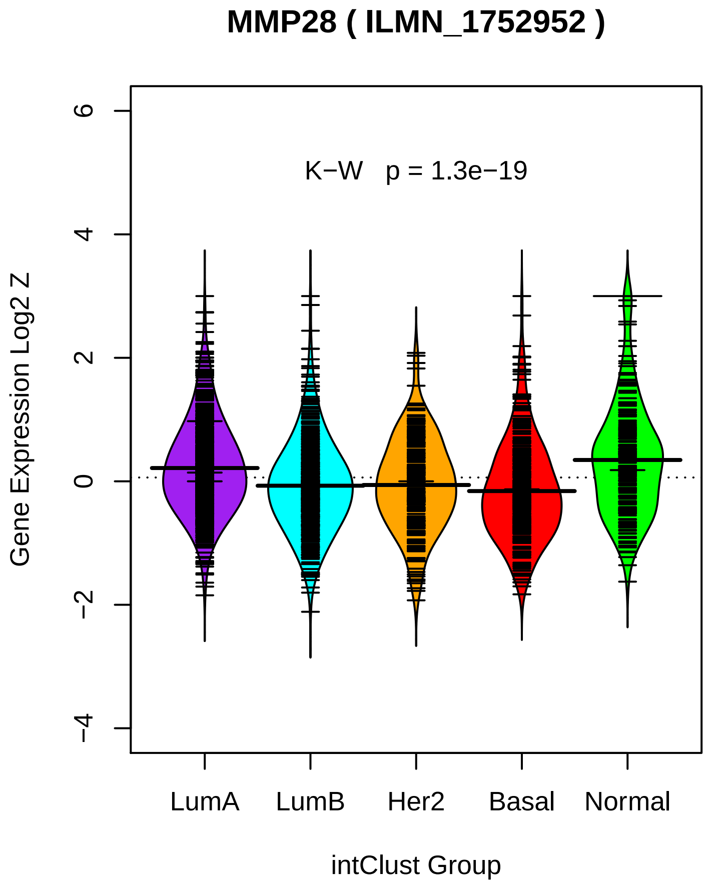
MMP28 (ILMN_1752952)
K−W p = 1.3e−19
Gene Expression Log2 Z
6
4
2
0
−2
−4
LumA
LumB
Her2
Basal
Normal
intClust Group

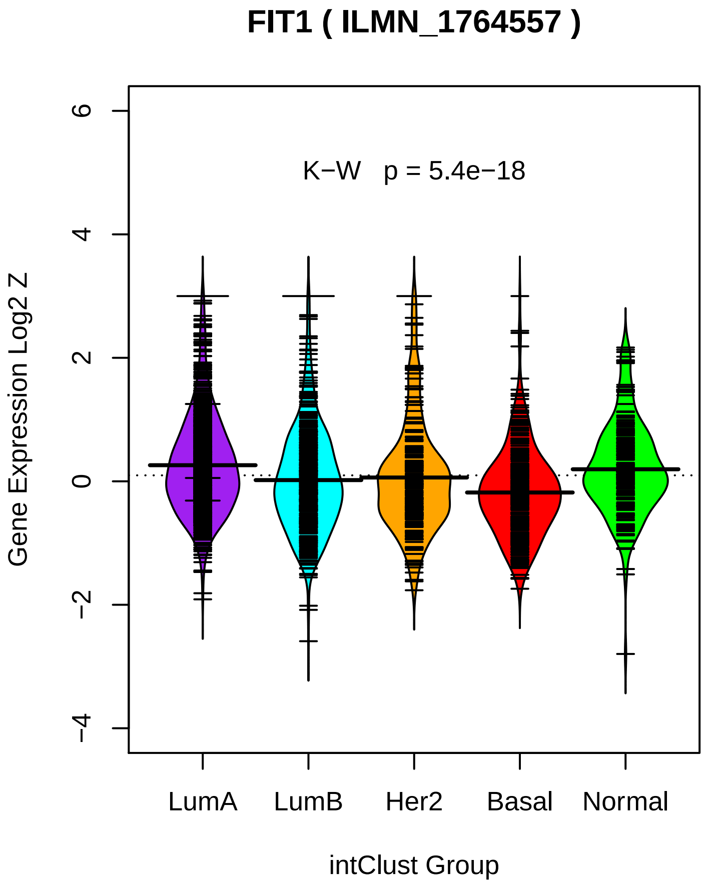
FIT1 (ILMN_1764557)
K−W p = 5.4e−18
Gene Expression Log2 Z
6
4
2
0
−2
−4
LumA
LumB
Her2
Basal
Normal
intClust Group

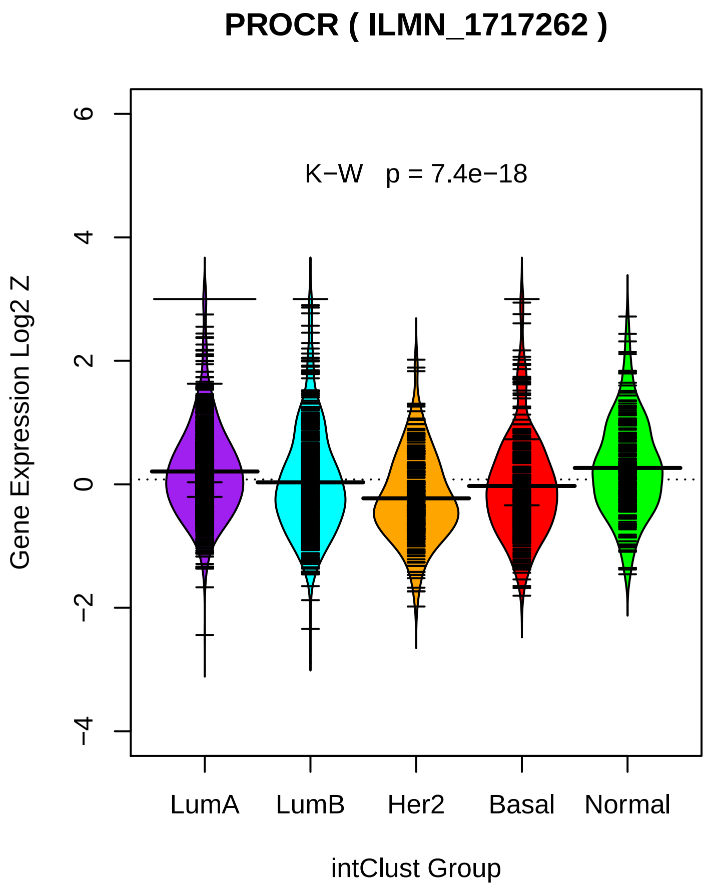
PROCR (ILMN_1717262)
K−W p = 7.4e−18
Gene Expression Log2 Z
6
4
2
0
−2
−4
LumA
LumB
Her2
Basal
Normal
intClust Group

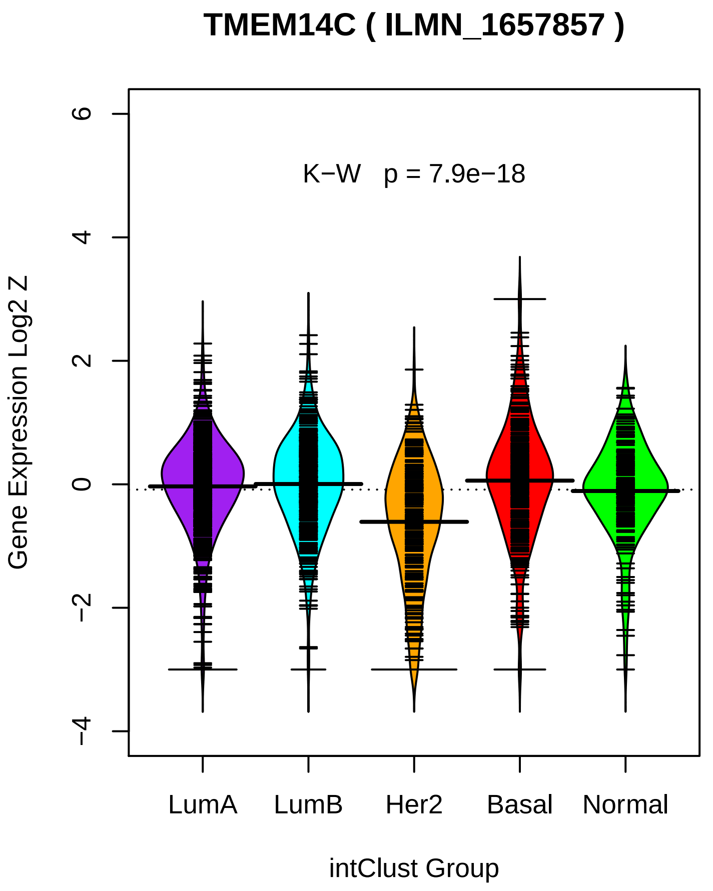
TMEM14C (ILMN_1657857)
K−W p = 7.9e−18
Gene Expression Log2 Z
6
4
2
0
−2
−4
LumA
LumB
Her2
Basal
Normal
intClust Group

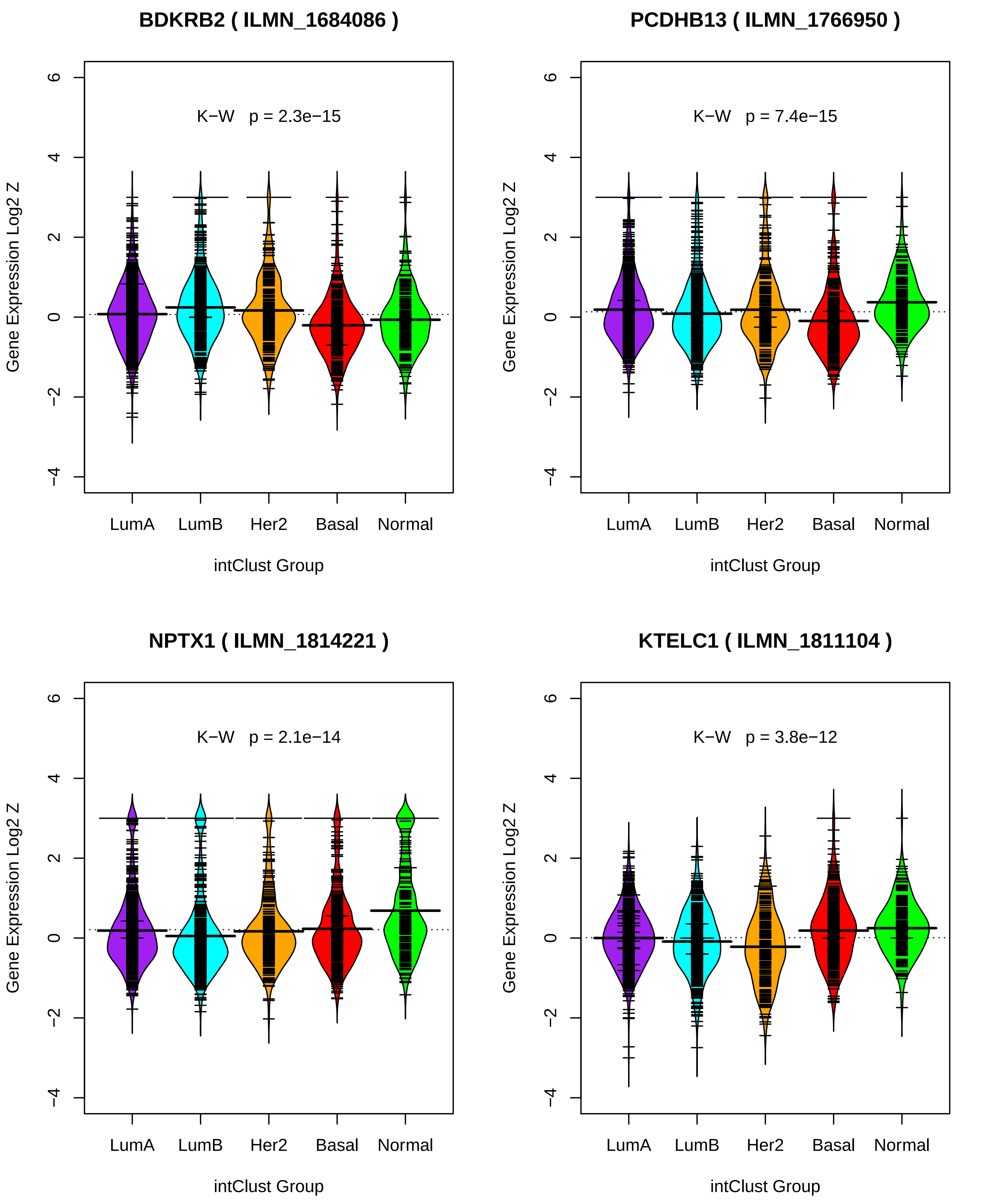
BDKRB2 (ILMN_1684086)
K−W p = 2.3e−15
PCDHB13 (ILMN_1766950)
K−W p = 7.4e−15
NPTX1 (ILMN_1814221)
K−W p = 2.1e−14
KTELC1 (ILMN_1811104)
K−W p = 3.8e−12
Gene Expression Log2 Z
−4
−2
0
2
4
6
LumA
LumB
Her2
Basal
Normal
intClust Group

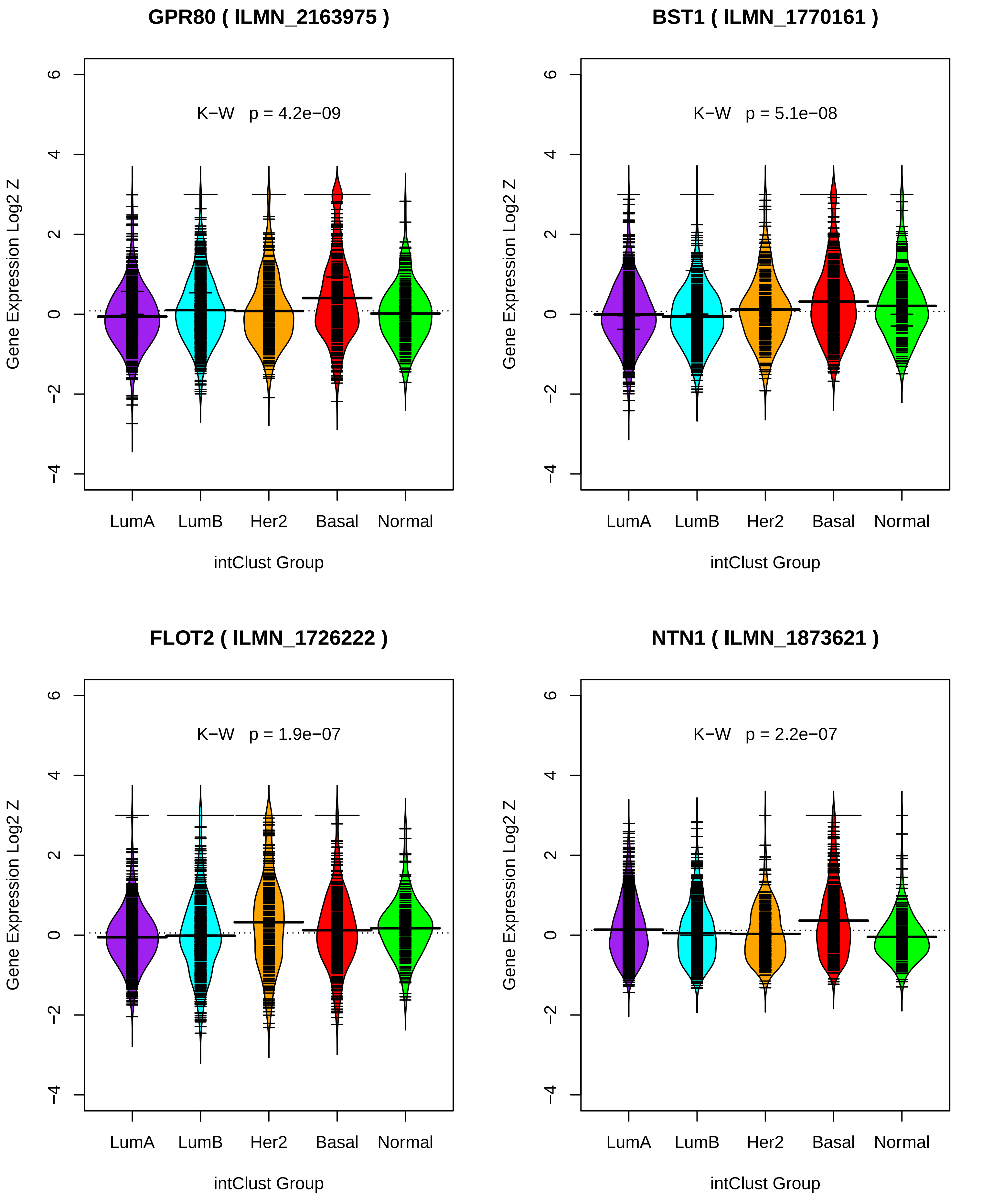
GPR80 (ILMN_2163975)
K−W p = 4.2e−09
BST1 (ILMN_1770161)
K−W p = 5.1e−08
FLOT2 (ILMN_1726222)
K−W p = 1.9e−07
NTN1 (ILMN_1873621)
K−W p = 2.2e−07
Gene Expression Log2 Z
LumA
LumB
Her2
Basal
Normal
intClust Group

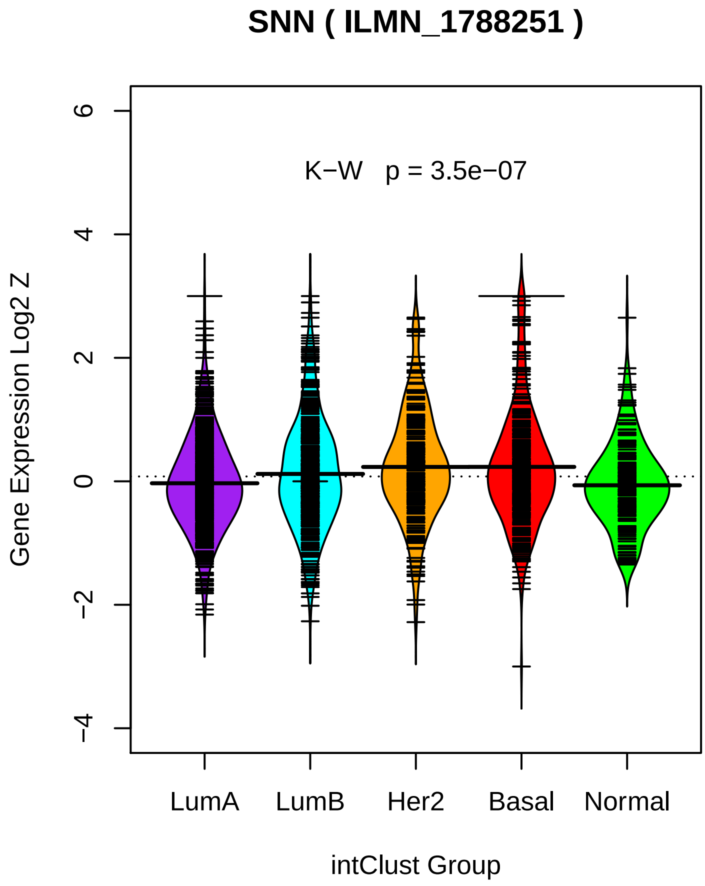
SNN (ILMN_1788251)
K−W p = 3.5e−07
Gene Expression Log2 Z
6
4
2
0
−2
−4
LumA
LumB
Her2
Basal
Normal
intClust Group

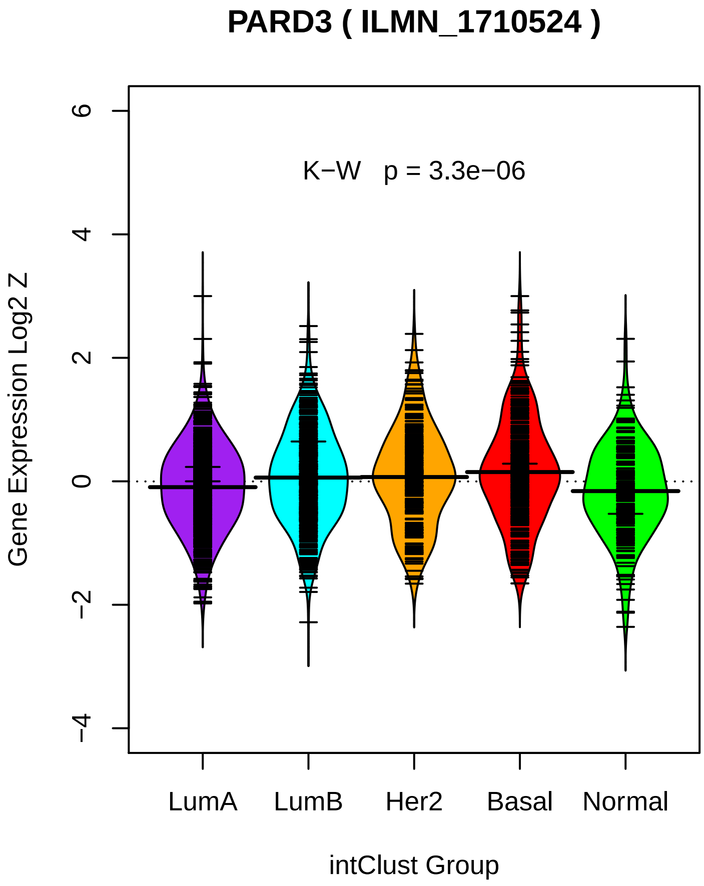
PARD3 (ILMN_1710524)
K−W p = 3.3e−06
Gene Expression Log2 Z
6
4
2
0
−2
−4
LumA
LumB
Her2
Basal
Normal
intClust Group

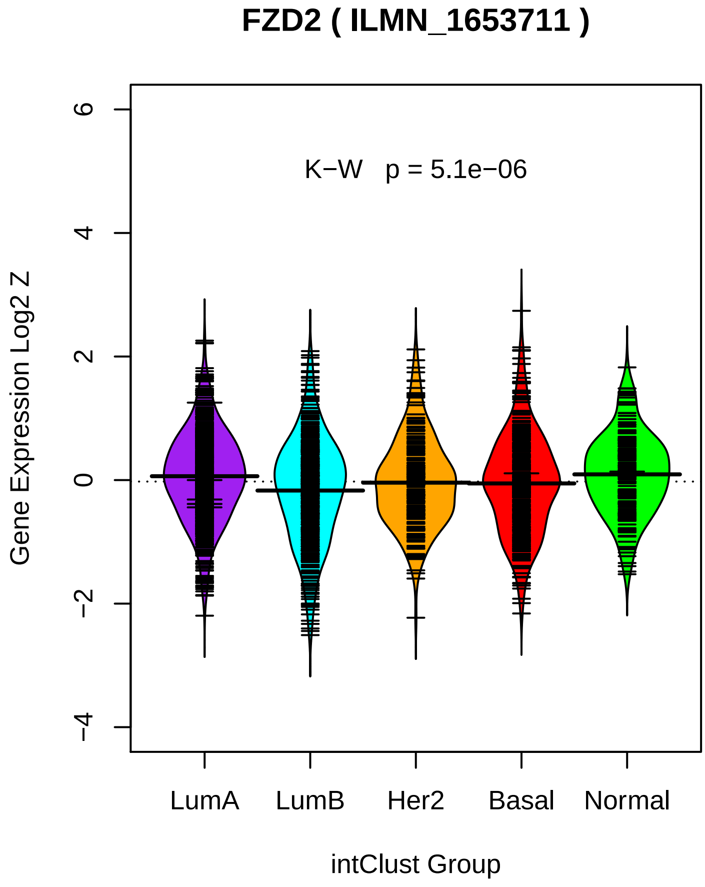
FZD2 (ILMN_1653711)
K−W p = 5.1e−06
Gene Expression Log2 Z
6
4
2
0
−2
−4
LumA
LumB
Her2
Basal
Normal
intClust Group

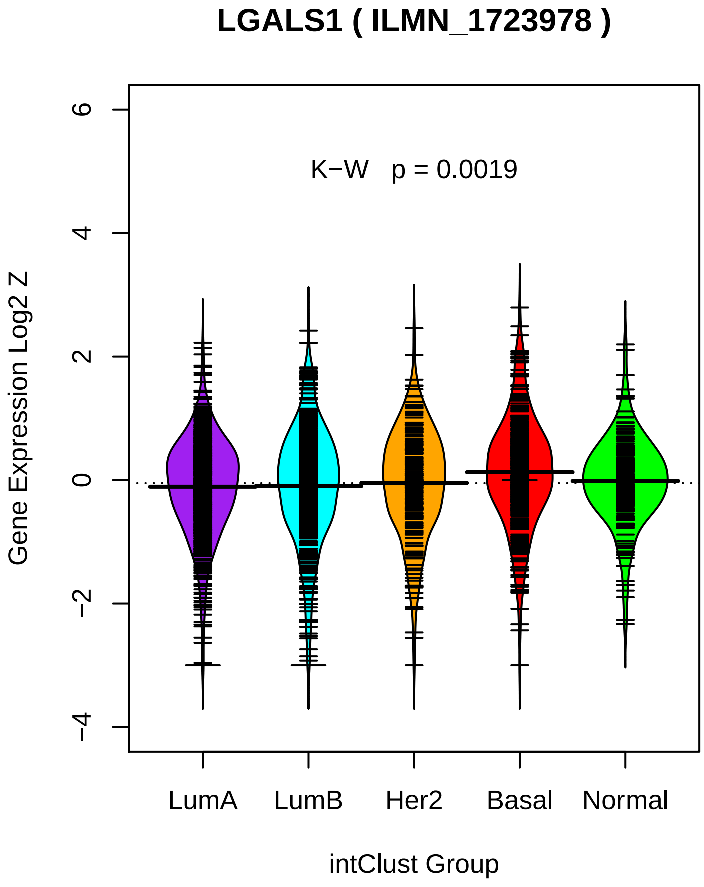
LGALS1 (ILMN_1723978)
K−W p = 0.0019
Gene Expression Log2 Z
6
4
2
0
−2
−4
LumA
LumB
Her2
Basal
Normal
intClust Group

CD79A (ILMN_2410371)

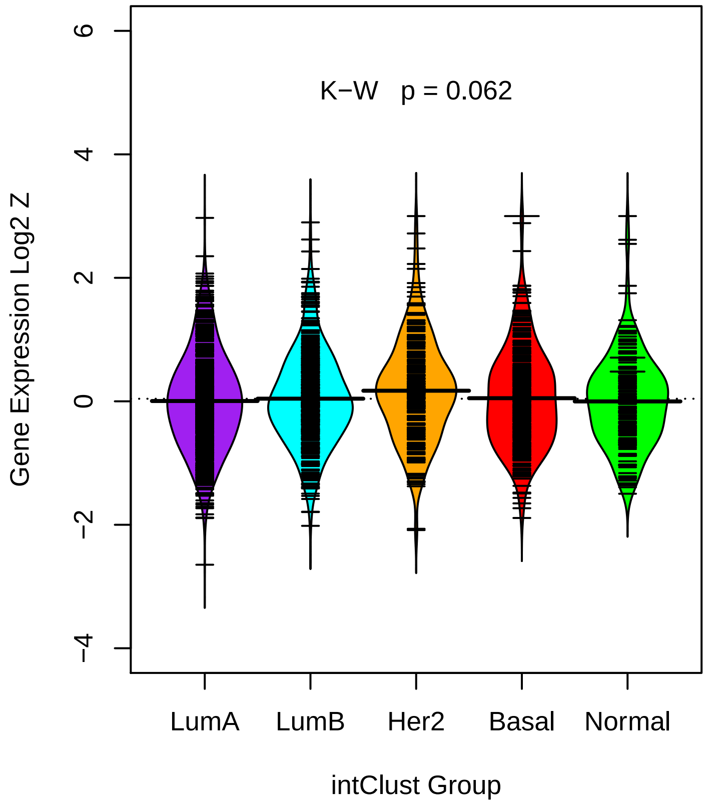

PLA2G2F (ILMN_1724799)

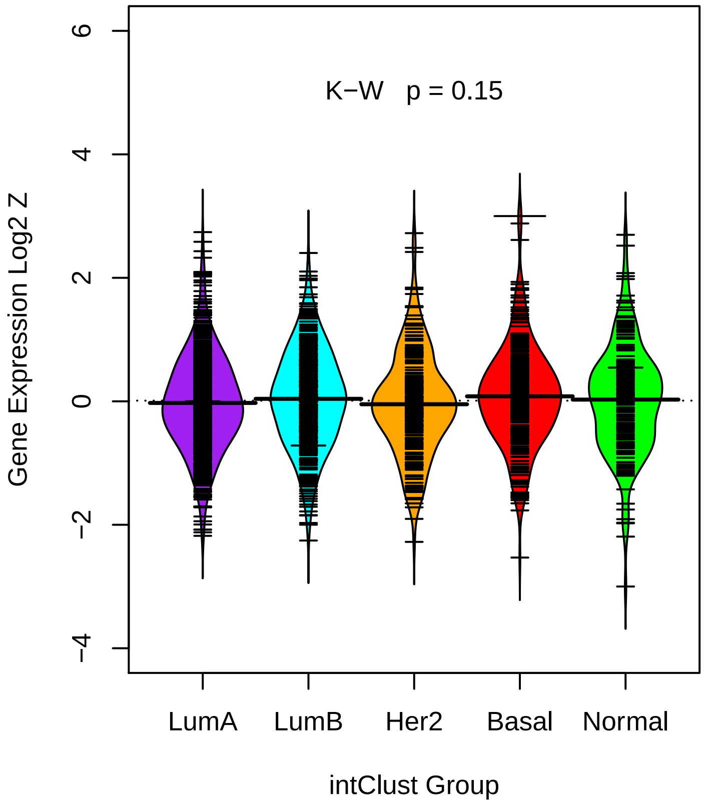

KCNJ5 (ILMN_2137312)

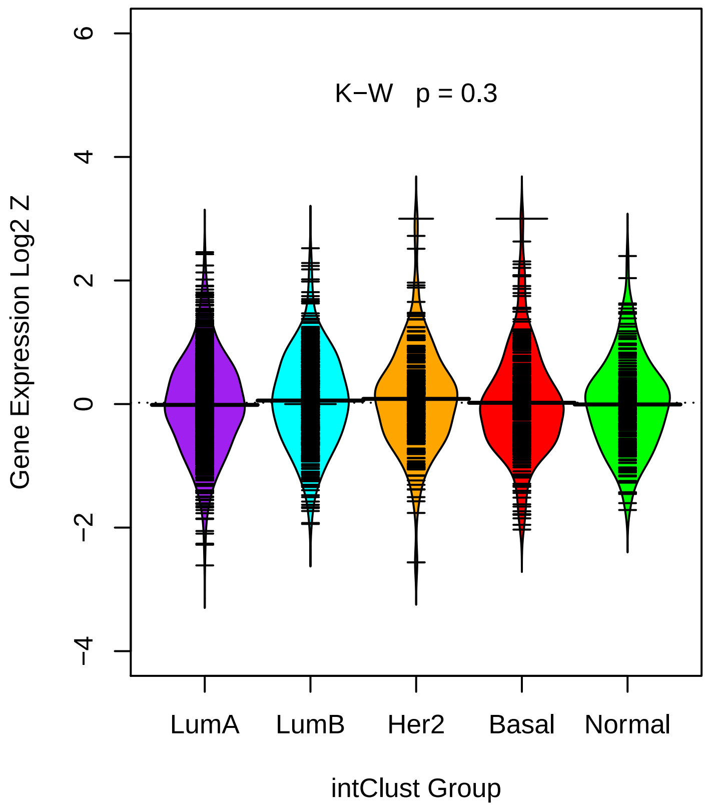

NTN2L (ILMN_1656040)

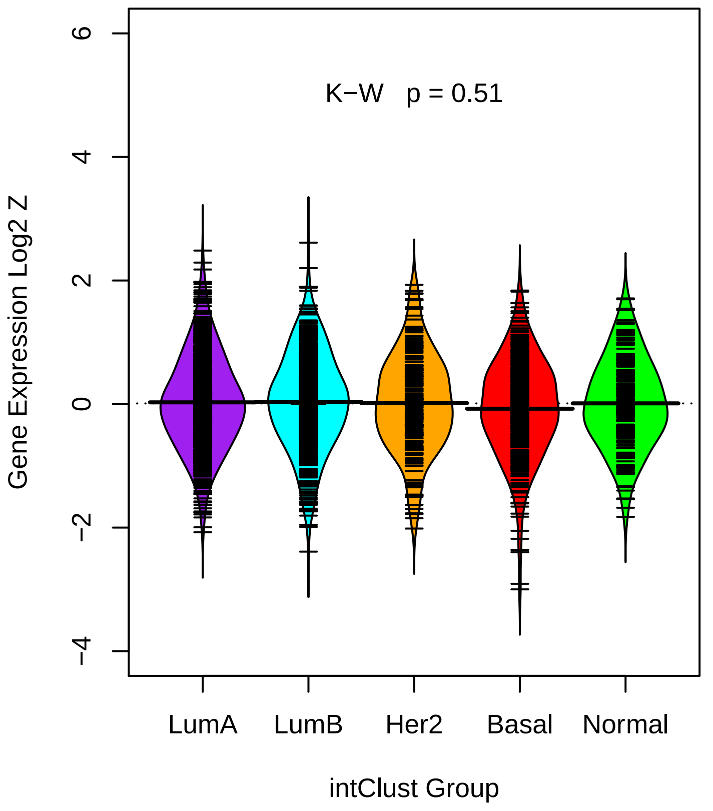

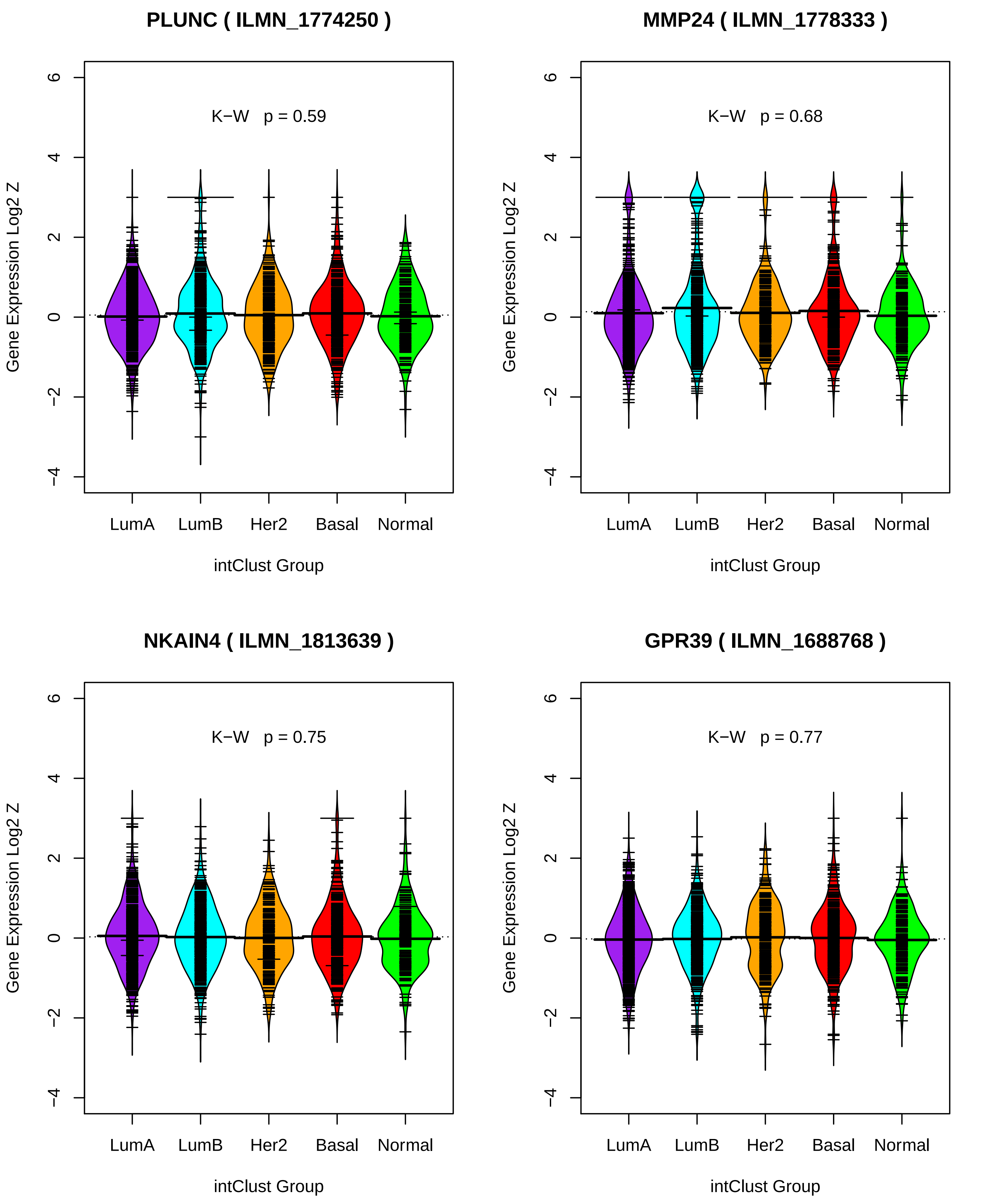
PLUNC (ILMN_1774250)
K−W p = 0.59
MMP24 (ILMN_1778333)
K−W p = 0.68
NKAIN4 (ILMN_1813639)
K−W p = 0.75
GPR39 (ILMN_1688768)
K−W p = 0.77
Gene Expression Log2 Z
6
4
2
0
−2
−4
LumA
LumB
Her2
Basal
Normal
intClust Group

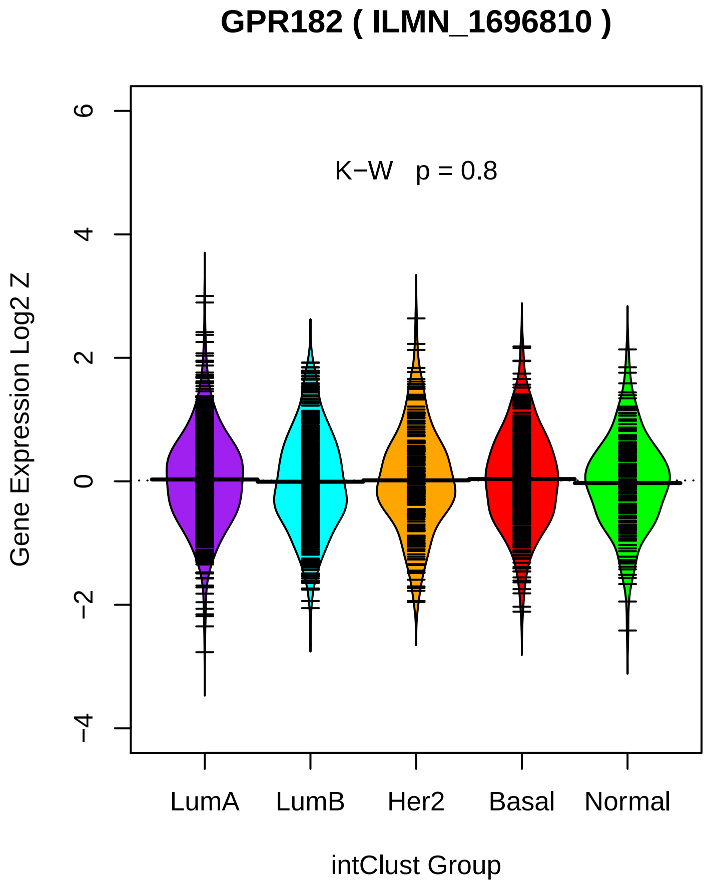
GPR182 (ILMN_1696810)
K−W p = 0.8
Gene Expression Log2 Z
6
4
2
0
−2
−4
LumA
LumB
Her2
Basal
Normal
intClust Group

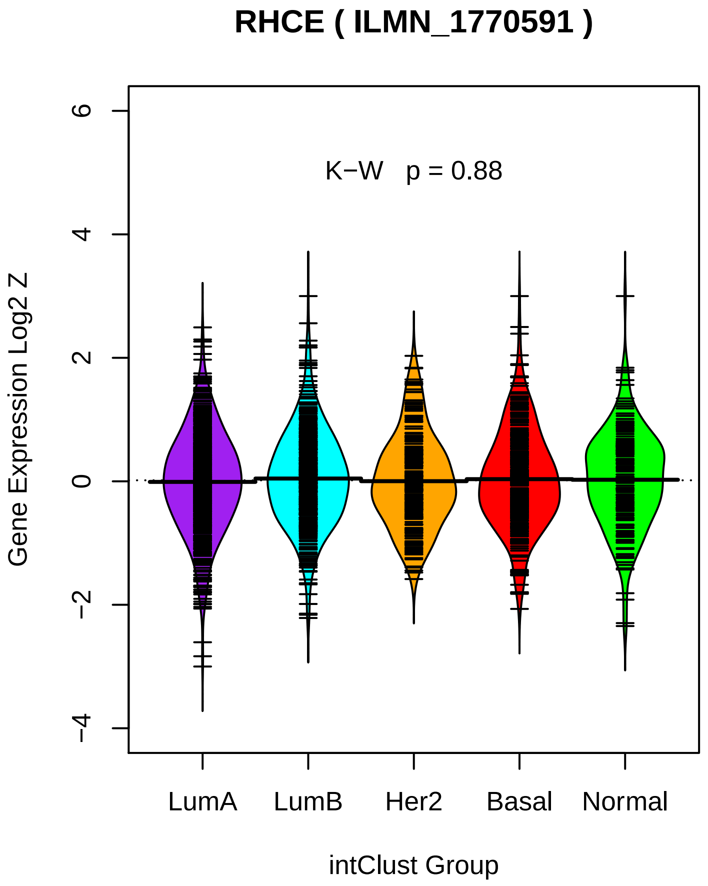
RHCE (ILMN_1770591)
K−W p = 0.88
Gene Expression Log2 Z
6
4
2
0
−2
−4
LumA
LumB
Her2
Basal
Normal
intClust Group
