# Supplementary material for: A co-culture genome-wide RNAi screen with mammary epithelial cells reveals transmembrane signals required for growth and differentiation
Source: Breast Cancer Res. 2015 Jan 9;17:4. doi: 10.1186/s13058-014-0510-y (PMC4322558; doi:10.1186/s13058-014-0510-y)
Supplement: Additional file 14: Figure S11. — Independent disease outcome associations are present with select target genes. Individual genes shown in Figure 9C are depicted with significant survival differences with low (green) versus high (red) gene expression for both integrative cluster (IntClust) breast cancer subtype groups and PAM50 subtypes. Associations were assessed by Kaplan-Meier G-rho-stratified analysis and by Cox proportional hazards analysis, after adjustment for multiple comparisons using the method of Benjamini and Hochberg [26]. CD79A expression shows improved survival for IntClusts 8 and 10 and HER2. KCNJ5 expression shows poorer survival for IntClusts 4 and 8. SERPINH1 expression shows poorer survival for IntClusts 6, 9 and 10. TMEM14C expression shows improved survival for IntClust 4 and luminal B. [file 13058_2014_510_MOESM14_ESM.pdf]

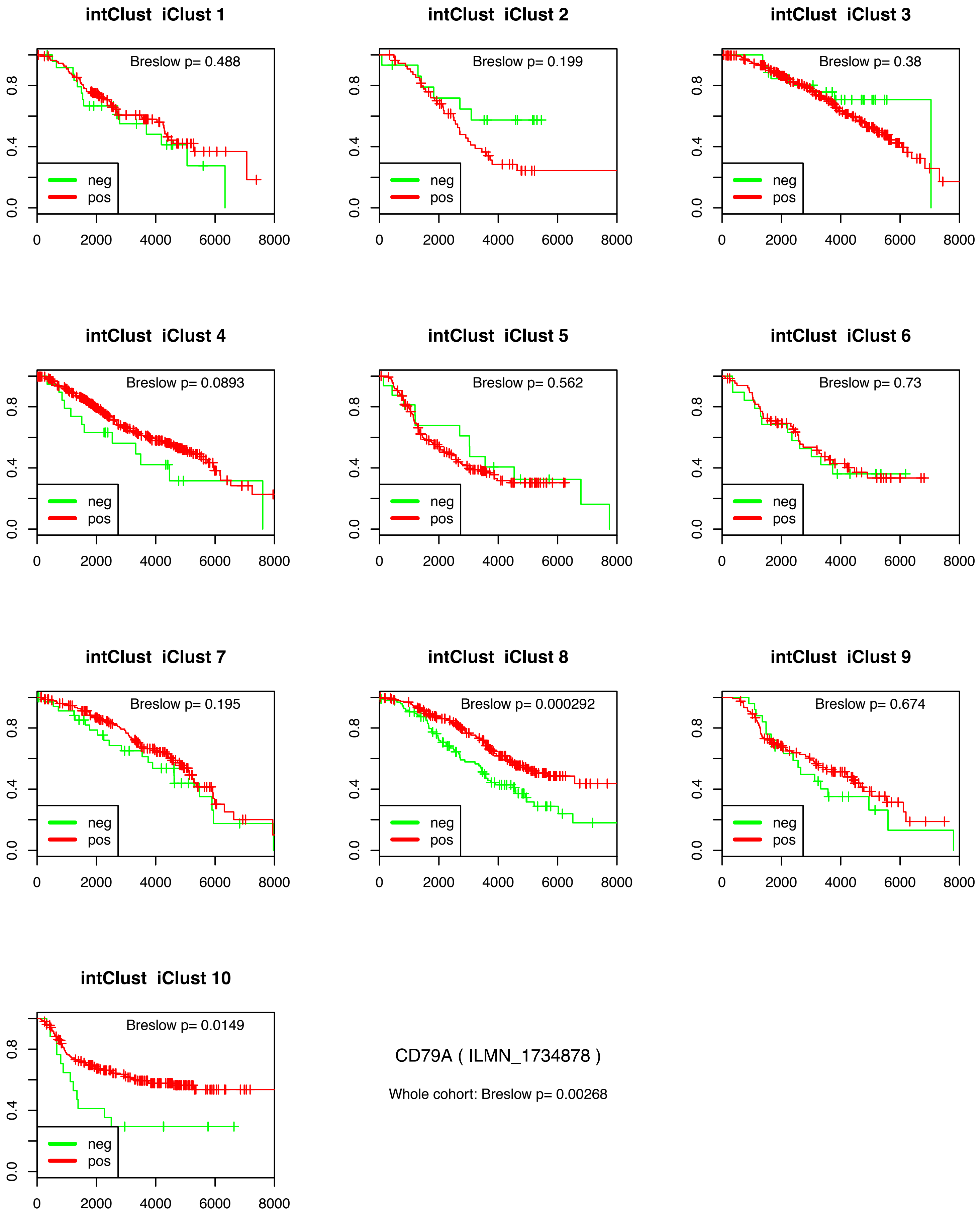
intClust iClust 1
Breslow p= 0.488
intClust iClust 2
Breslow p= 0.199
intClust iClust 3
Breslow p= 0.38
intClust iClust 4
Breslow p= 0.0893
intClust iClust 5
Breslow p= 0.562
intClust iClust 6
Breslow p= 0.73
intClust iClust 7
Breslow p= 0.195
intClust iClust 8
Breslow p= 0.000292
intClust iClust 9
Breslow p= 0.674
intClust iClust 10
Breslow p= 0.0149
neg
pos
0.0
0.4
0.8
0
2000
4000
6000
8000
CD79A (ILMN_1734878)
Whole cohort: Breslow p= 0.00268

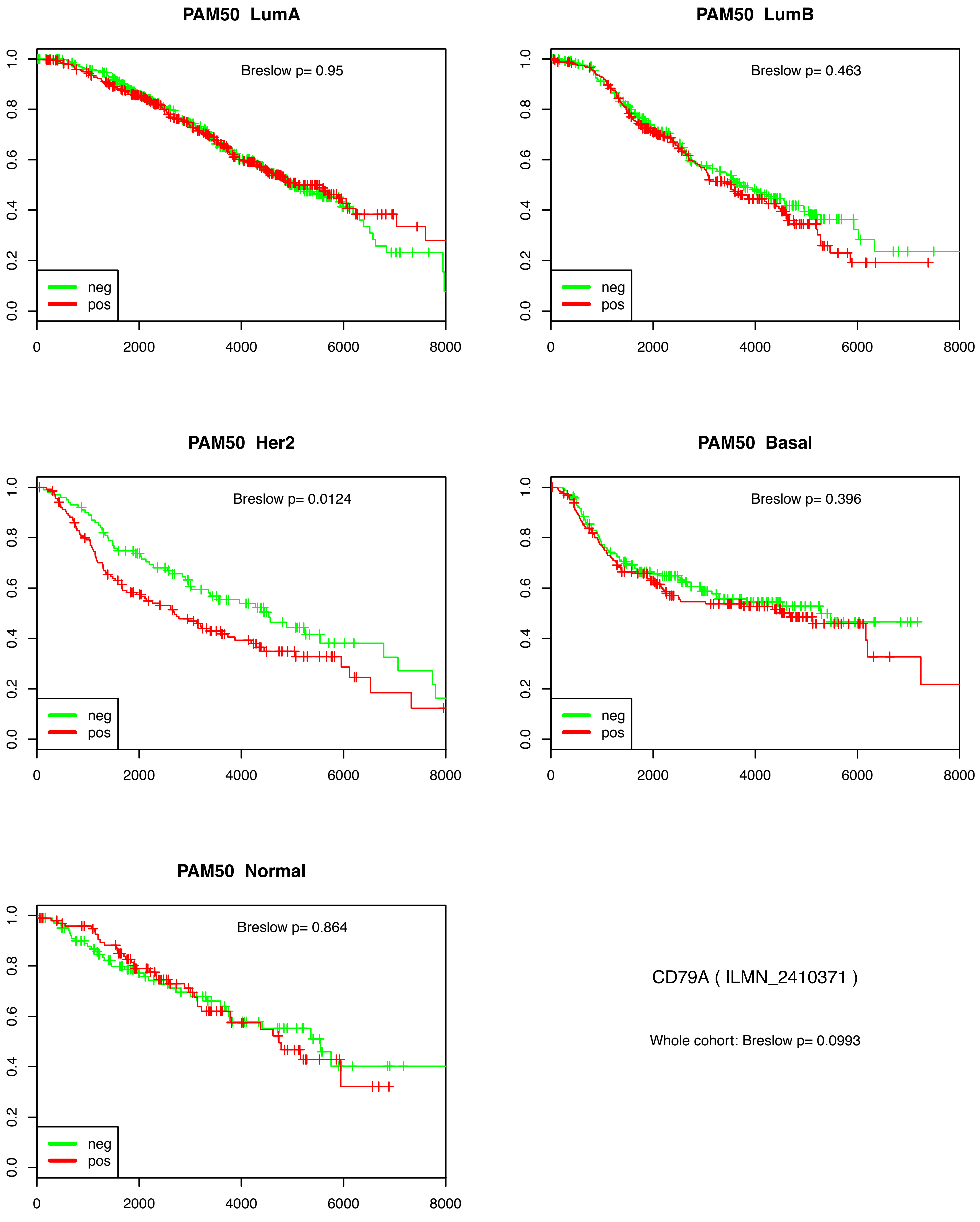
PAM50 LumA
Breslow p= 0.95
PAM50 LumB
Breslow p= 0.463
PAM50 Her2
Breslow p= 0.0124
PAM50 Basal
Breslow p= 0.396
PAM50 Normal
Breslow p= 0.864
neg
pos
0.0
0.2
0.4
0.6
0.8
1.0
0
2000
4000
6000
8000
CD79A (ILMN_2410371)
Whole cohort: Breslow p= 0.0993

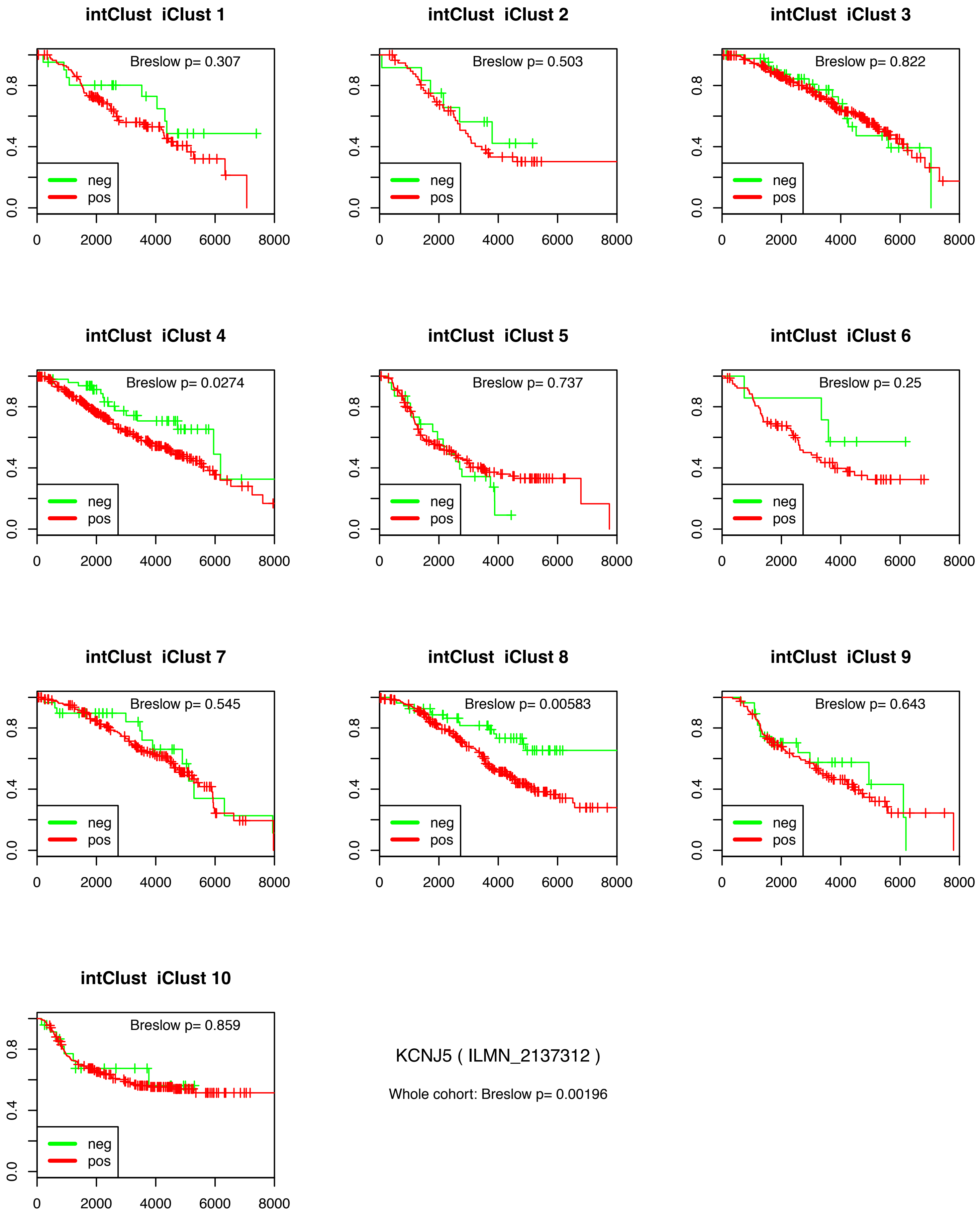
intClust iClust 1
Breslow p= 0.307
intClust iClust 2
Breslow p= 0.503
intClust iClust 3
Breslow p= 0.822
intClust iClust 4
Breslow p= 0.0274
intClust iClust 5
Breslow p= 0.737
intClust iClust 6
Breslow p= 0.25
intClust iClust 7
Breslow p= 0.545
intClust iClust 8
Breslow p= 0.00583
intClust iClust 9
Breslow p= 0.643
intClust iClust 10
Breslow p= 0.859
neg
pos
0.0
0.4
0.8
0
2000
4000
6000
8000
KCNJ5 (ILMN_2137312)
Whole cohort: Breslow p= 0.00196

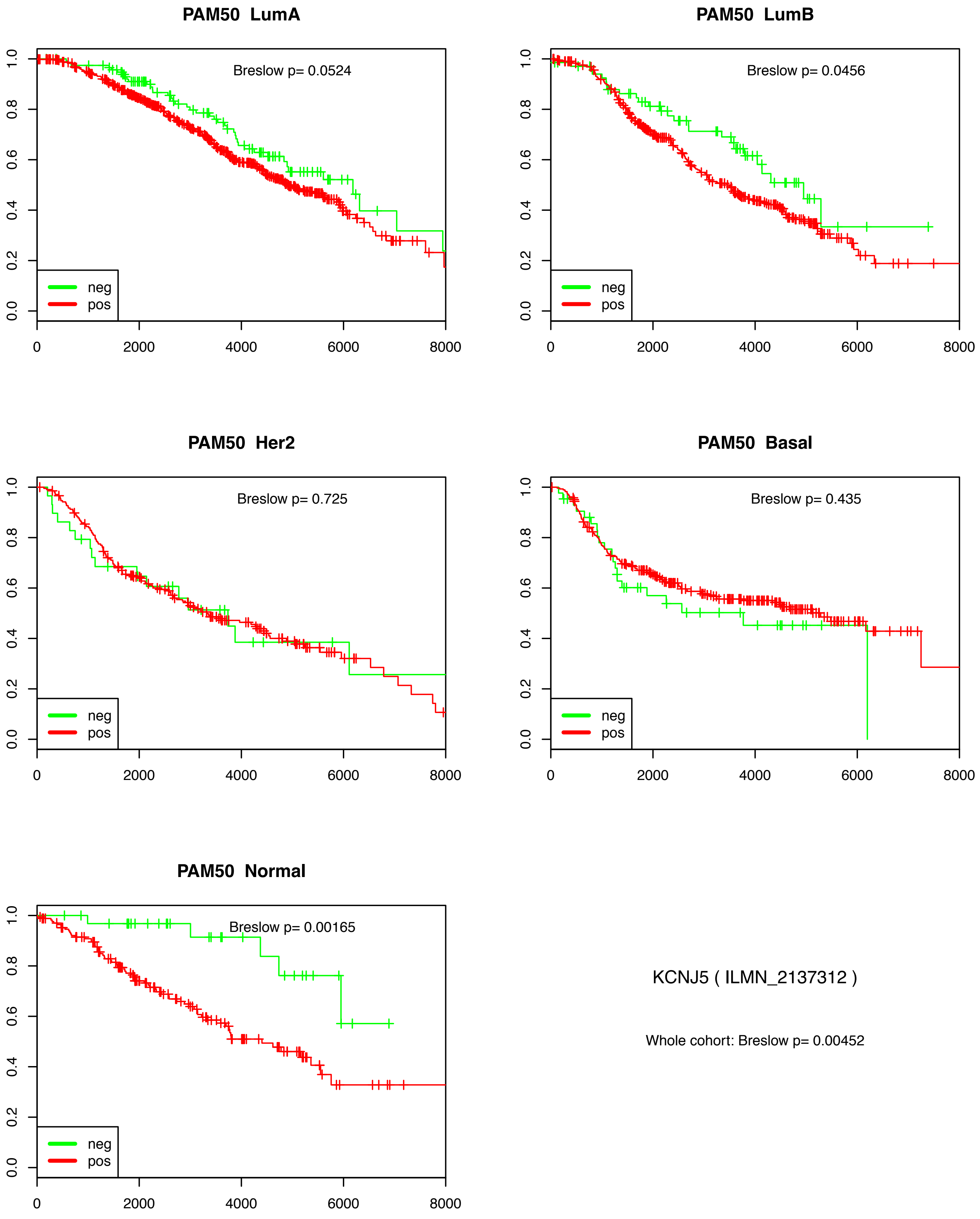
PAM50 LumA
Breslow p= 0.0524
neg
pos
PAM50 LumB
Breslow p= 0.0456
neg
pos
PAM50 Her2
Breslow p= 0.725
neg
pos
PAM50 Basal
Breslow p= 0.435
neg
pos
PAM50 Normal
Breslow p= 0.00165
neg
pos
0.0 0.2 0.4 0.6 0.8 1.0
0 2000 4000 6000 8000
KCNJ5 (ILMN_2137312)
Whole cohort: Breslow p= 0.00452

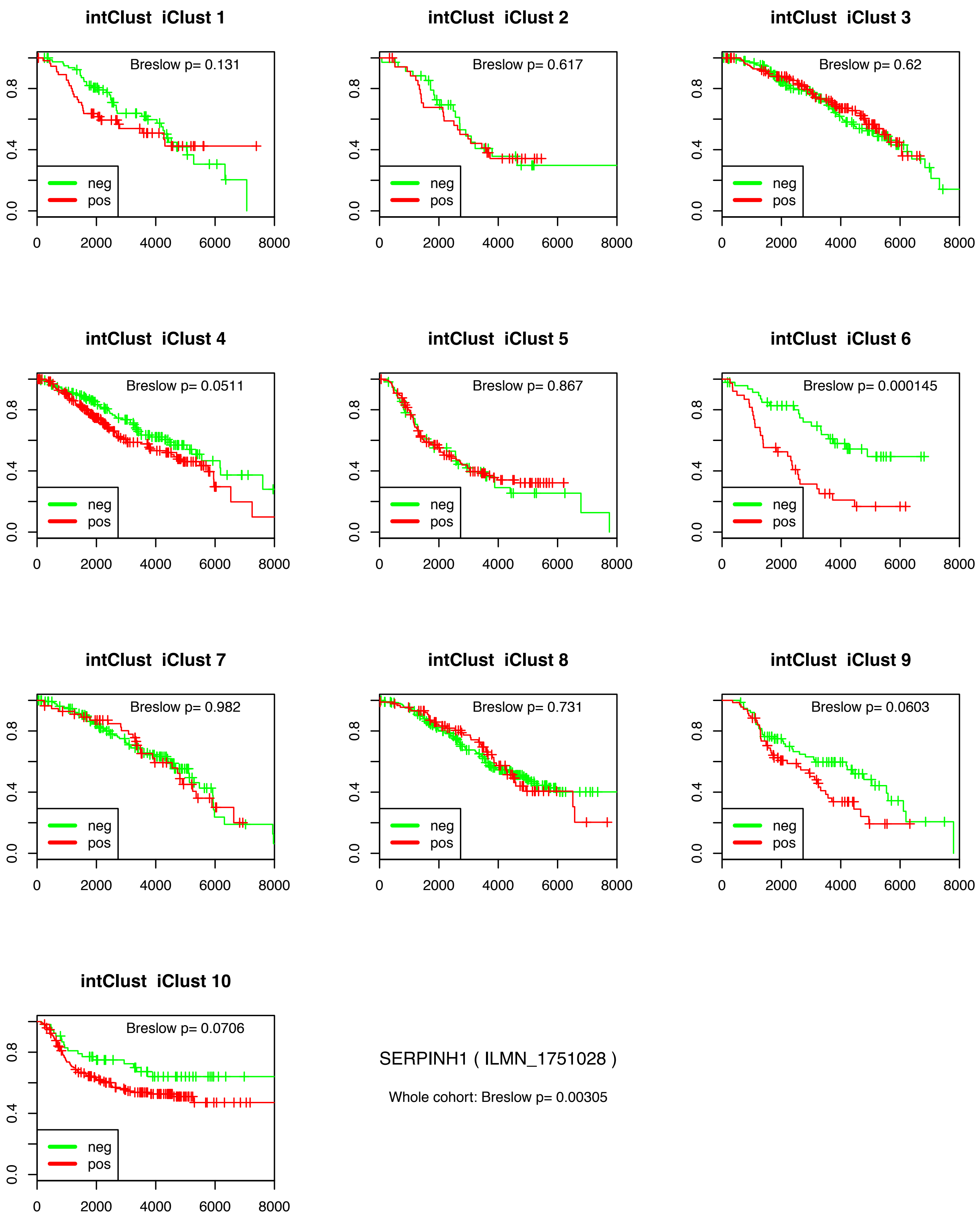
intClust iClust 1
Breslow p= 0.131
intClust iClust 2
Breslow p= 0.617
intClust iClust 3
Breslow p= 0.62
intClust iClust 4
Breslow p= 0.0511
intClust iClust 5
Breslow p= 0.867
intClust iClust 6
Breslow p= 0.000145
intClust iClust 7
Breslow p= 0.982
intClust iClust 8
Breslow p= 0.731
intClust iClust 9
Breslow p= 0.0603
intClust iClust 10
Breslow p= 0.0706
neg
pos
0.0
0.4
0.8
0
2000
4000
6000
8000
SERPINH1 (ILMN_1751028)
Whole cohort: Breslow p= 0.00305

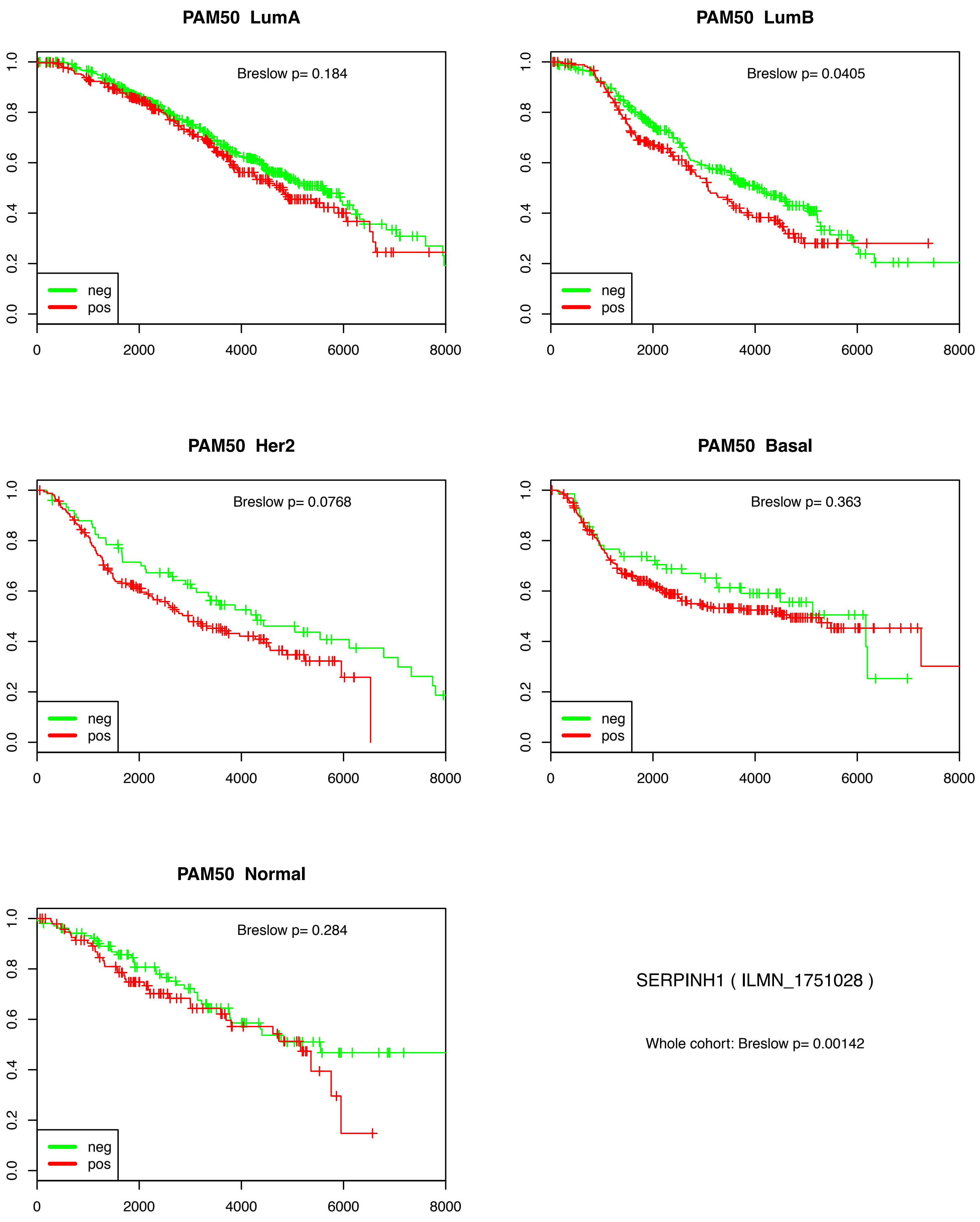
PAM50 LumA
Breslow p= 0.184
neg
pos
PAM50 LumB
Breslow p= 0.0405
neg
pos
PAM50 Her2
Breslow p= 0.0768
neg
pos
PAM50 Basal
Breslow p= 0.363
neg
pos
PAM50 Normal
Breslow p= 0.284
neg
pos
SERPINH1 (ILMN_1751028)
Whole cohort: Breslow p= 0.00142

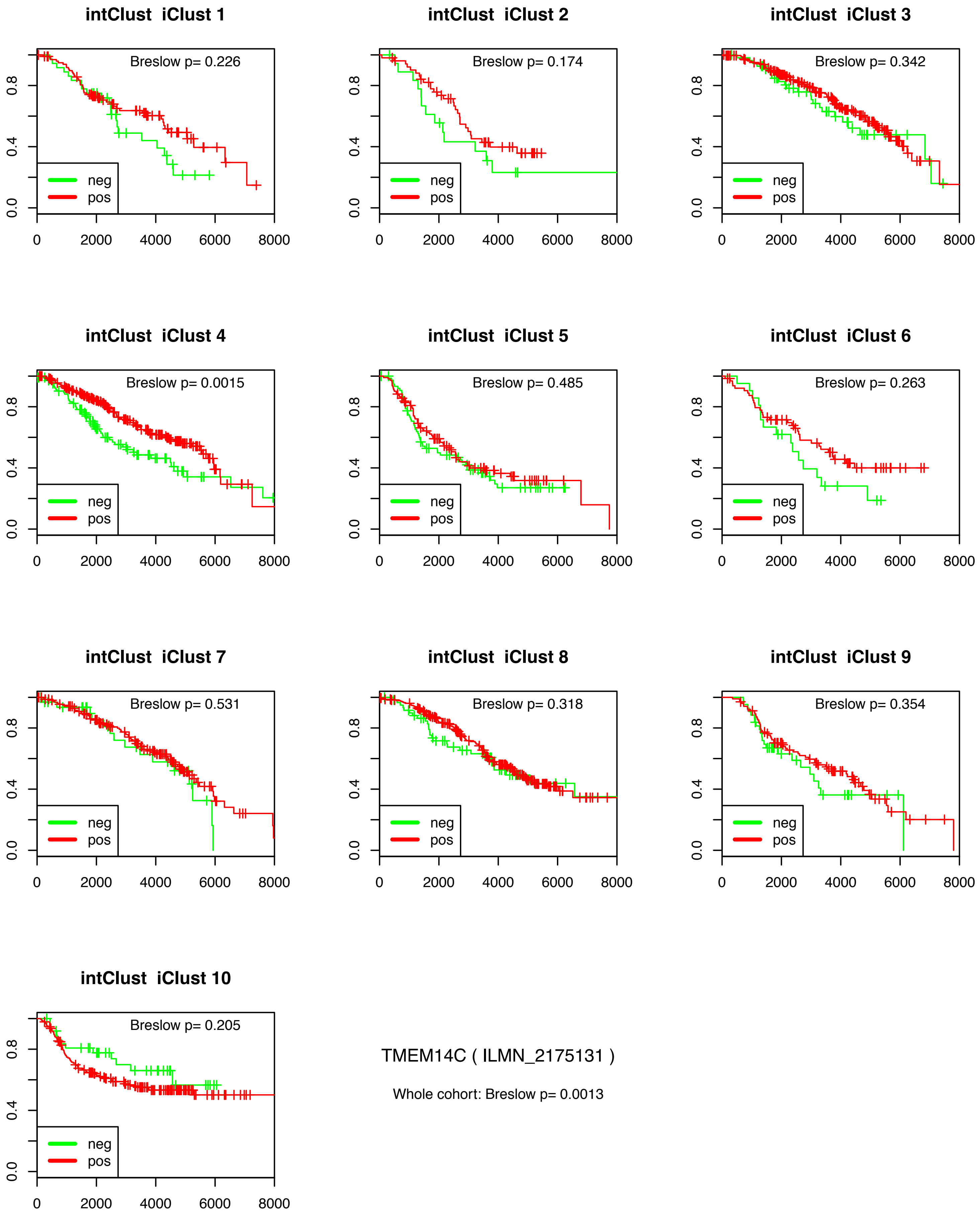
intClust iClust 1
Breslow p= 0.226
intClust iClust 2
Breslow p= 0.174
intClust iClust 3
Breslow p= 0.342
intClust iClust 4
Breslow p= 0.0015
intClust iClust 5
Breslow p= 0.485
intClust iClust 6
Breslow p= 0.263
intClust iClust 7
Breslow p= 0.531
intClust iClust 8
Breslow p= 0.318
intClust iClust 9
Breslow p= 0.354
intClust iClust 10
Breslow p= 0.205
neg
pos
0.0
0.4
0.8
0
2000
4000
6000
8000
TMEM14C (ILMN_2175131)
Whole cohort: Breslow p= 0.0013

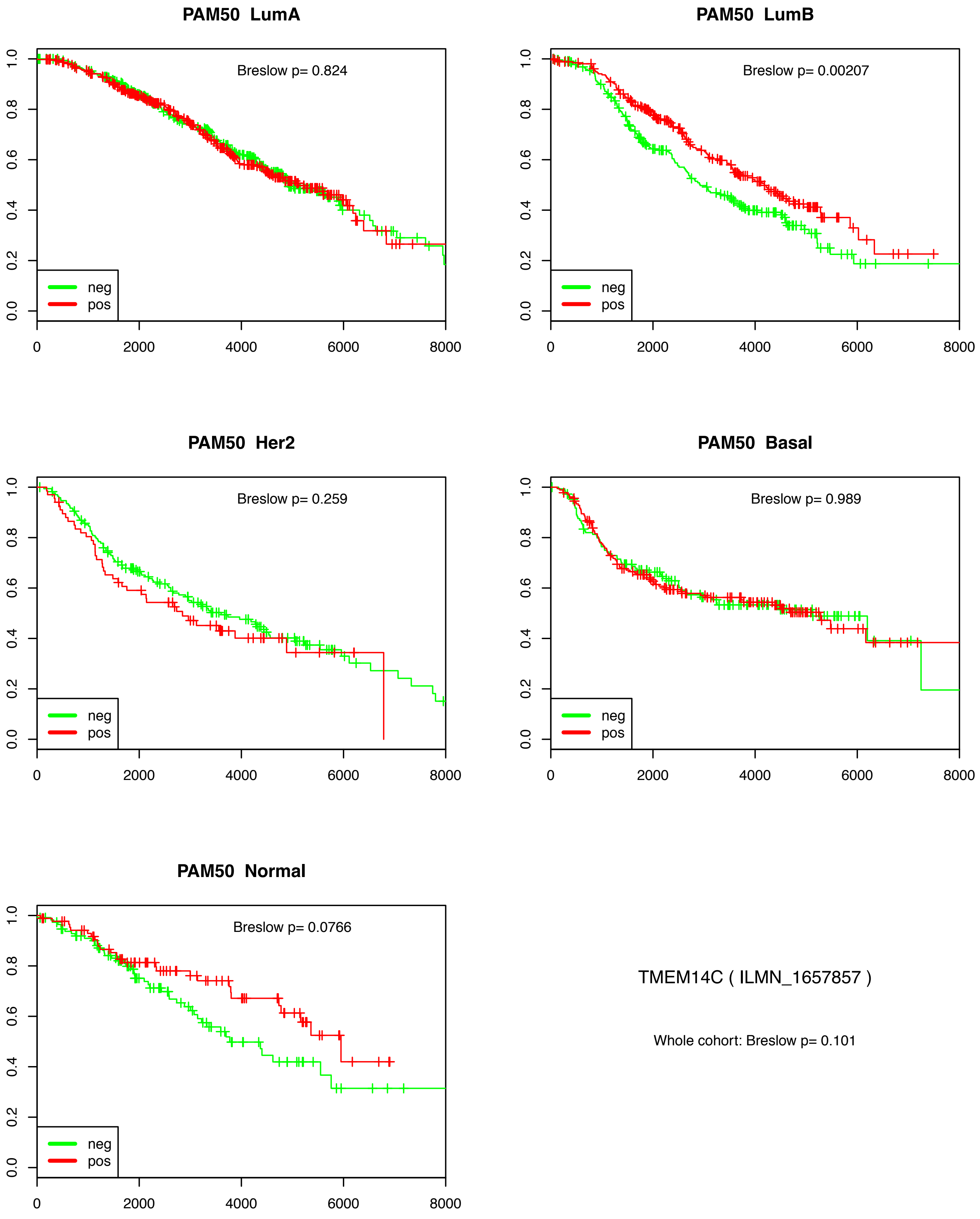
PAM50 LumA
Breslow p= 0.824
neg
pos
PAM50 LumB
Breslow p= 0.00207
neg
pos
PAM50 Her2
Breslow p= 0.259
neg
pos
PAM50 Basal
Breslow p= 0.989
neg
pos
PAM50 Normal
Breslow p= 0.0766
neg
pos
TMEM14C (ILMN_1657857)
Whole cohort: Breslow p= 0.101
